# Tracking the narrative: A data-driven analysis of media coverage of Russia and Ukraine 2013–2024

**DOI:** 10.1371/journal.pone.0351627

**Published:** 2026-06-25

**Authors:** Irene Vianini, Sopho Kharazi, Bonka Kotseva, Kristina Kovacikova, Nicolò Faggiani, Nikolaos Nikolaidis, Leonida Della Rocca, Kristina Potapova, Olena Snigyr, Jens P. Linge

**Affiliations:** 1 Piksel S.r.l., Ispra, Italy; 2 European Commission, Joint Research Centre, Unit T.5, Ispra, Italy; 3 Engineering S.p.A., Rome, Italy; 4 Athens University of Economics and Business, Athens, Greece; 5 European Parliament, Directorate-General for Communication, Informatics Unit, Brussels, Belgium; 6 University College London (UCL), School of Slavonic and East European Studies, London, United Kingdom; 7 European University Institute (EUI), Robert Schuman Centre for Advanced Studies, Florence, Italy; Universiti Pertahanan Nasional Malaysia, MALAYSIA

## Abstract

This study addresses the lack of global, long-term analyses of media narratives on Russia and Ukraine by examining their evolution from 2013 to 2024. Based on 22.2 million article titles from 14,622 sources operating in 194 countries and covering up to 74 languages, this study employs a hybrid human-AI approach combining multilingual clustering, cluster linking, manual annotation, entity analysis, and analysis of the language distribution of articles by Russian sources. We show that media coverage increased sharply around key geopolitical events, including the Euromaidan protests (2013–2014), the annexation of Crimea (2014), and Russia’s full-scale invasion of Ukraine (2022). Multilingual clustering, in combination with manual annotation, identifies recurring patterns in coverage, including geopolitical tensions between Russia and the West, energy security, and disinformation campaigns. Cluster linking reveals how these patterns evolved over time in response to major events: clusters in 2013–2014 focused on the Russia–Ukraine gas dispute, the Euromaidan protests, and Crimea, while those in 2021–2022 centred on the full-scale invasion and its global repercussions. Entity analysis shows that media attention consistently concentrated on a small set of political figures, including Vladimir Putin, Volodymyr Zelenskyy, and Donald Trump, whose prominence varied across different phases of the conflict and key political events. Analysis of Russian media further indicates sustained publication in foreign languages, with increases during key geopolitical moments and differences in how the conflict is contextualised across language groups. Despite uneven regional and linguistic coverage, this study provides a large-scale, long-term, and multilingual overview of media coverage. Future research could extend this work through cross-country comparisons, linking narrative shifts to external variables, such as public opinion data or electoral outcomes, and applying the methodology to other large-scale datasets.

## Introduction

In today’s information environment, the vast volume of content created and disseminated at an unprecedented speed compels researchers to employ more efficient data analysis methods that integrate human-machine interaction. Hybrid human-Artificial Intelligence (AI) approaches are especially significant in the context of Russia’s war against Ukraine — a conflict fought not only on the battlefield but also across informational and semantic domains. This study leverages such an approach to explore how recurring narrative patterns can be identified and traced across media coverage over time.

More broadly, contemporary conflicts unfold within a global contest of narratives that extends beyond any single case and contributes to shaping perceptions of the international order. Leading actors, such as Russia, China, and others, employ strategic narratives as instruments of influence and as mechanisms for maintaining agency and legitimacy within an evolving international system [1]. This aligns with the logic of ontological security, whereby states seek to reduce existential uncertainty by sustaining coherent self-narratives and stable patterns of interaction [2–4]. Strategic narratives in international relations are conceptualised as communicative tools through which political actors construct shared meanings about past, present, and future events to achieve political objectives [5]. This perspective underscores the role of narratives in shaping political communication and collective identity [6].

To analyse such processes in large-scale media corpora, this study integrates three complementary theoretical perspectives. Framing theory [7,8] helps identify which aspects of events become salient and how issues are described in media coverage. Mediatisation theory [9,10] draws attention to how media environments amplify, stabilise, and circulate these patterns of meaning. Strategic narrative theory [5] provides a bridge from individual frames to broader patterns through which events are linked, interpreted, and given temporal coherence. Taken together, these approaches enable the study of narratives as evolving structures of meaning shaped by repetition, circulation, and reinterpretation across time and media systems.

Given the scale of the dataset, detailed qualitative reconstruction of narratives is not feasible. The study therefore adopts an operational definition suited to large-scale media analysis: narratives are defined as recurring patterns in how issues are described and contextualised in media coverage over time. Whereas framing focuses on the selective emphasis of specific aspects, this approach captures broader and temporally evolving patterns that emerge across multiple related items. This definition directly links the theoretical framework to the analytical strategy employed below.

The existing literature has shown that computational approaches can effectively identify narrative patterns in media, although these possibilities have not been realised to the same extent across studies. Through sentiment analysis, topic modelling, and machine learning, researchers have demonstrated that media outlets differ not only in the topics they prioritise, but also in how they present events and which layers of meaning they reinforce. Ibrahim et al. [11], for example, compared the BBC and Al Jazeera and showed how geopolitical orientation shapes differences in coverage. Verbytska [12] analysed strategic framing in Euronews and the Kyiv Post using Latent Dirichlet Allocation and BERT over a one-year period. Hanley et al. [13] extended this line of research through a multi-platform quantitative analysis of Western, Russian, and Chinese media, while Oates et al. [14] combined computational analysis with human interpretation to assess narrative similarity between Russian propaganda and US concern about human rights abuses in media discourse on illegally occupied and annexed Crimea.

Other studies have examined adjacent dimensions of narrative characterisation using computational methods. Parmelee et al. [15] studied moral framing in English-language international broadcasting using bag-of-words approaches and the extended Moral Foundations Dictionary, while Ediz et al. [16] analysed topic distribution in British media. Parizek [17] advanced the field by mapping the visibility of international organisations (the EU, NATO, and the UN) in a dataset of 2.9 million articles from 2,247 media outlets across 202 countries, showing how institutional authority and member-state control shape narrative dynamics. Taken together, these studies demonstrate the capacity of computational methods to detect recurring textual patterns, while hybrid human-AI approaches enable the interpretation of these patterns as broader narrative structures.

At the same time, the existing literature remains limited in several respects that are central to this study. Much of it relies on relatively short time horizons, bounded corpora, single-country or single-platform cases, or a limited number of languages. A number of studies focus mainly on English-language material, while even comparative work is often restricted to a small number of outlets, countries, or media systems. As a result, there is still a lack of global, long-term, multilingual analyses of how narratives about Russia and Ukraine evolve across media ecosystems.

This empirical limitation also reflects a theoretical gap. While existing studies have generated important insights into framing differences, narrative similarity, and thematic variation, they remain only weakly connected to broader explanations of how narratives persist, travel, and evolve across time, languages, and media environments. In other words, a disconnect persists between three strands of research: studies of the sociopolitical, historical, and cultural impacts of narratives in media coverage [18]; theoretical accounts of narratives in international politics and national security [6,19,20]; and scalable empirical approaches capable of tracing narrative dynamics in very large multilingual corpora.

Addressing this gap requires integrating theoretical perspectives with scalable empirical methods. Framing theory [7,8], mediatisation theory [9,10], and strategic narrative theory [5] capture complementary dimensions of narrative dynamics — selective emphasis, cross-media circulation, and temporal coherence — yet are rarely integrated within large-scale empirical designs.

This study addresses this gap by proposing a hybrid human-AI analytical approach to the study of media narratives about Russia and Ukraine from 2013 to 2024, based on a large multilingual dataset. Methodologically, it combines multilingual clustering, cluster linking, and manual annotation. Conceptually, it shows how framing theory informs the analysis of recurring descriptions of events, mediatisation theory informs the analysis of their circulation and consolidation, and strategic narrative theory informs the identification of broader patterns of meaning over time. The study thus contributes both empirically, by providing a large-scale longitudinal analysis, and theoretically, by linking conceptual frameworks to computational methods.

To operationalise this approach, we first identify clusters of semantically similar articles based on textual similarity in article titles. These clusters capture recurring patterns in media coverage, reflecting similarities in topics and in how issues are described in headlines. We interpret clusters as stories, representing coherent and recurring ways in which issues are linked and described. Narratives are then understood as broader patterns emerging across multiple related stories over time.

Semantic similarity is computed automatically, and clusters provide a useful entry point for identifying recurring structures in media coverage. These patterns are subsequently interpreted and abstracted through expert analysis. The interpretation of clusters into stories and the identification of narratives both rely on this expert analysis, enabling us to identify dominant patterns and examine their evolution over time.

This process can be summarised as follows: articles (titles) → clusters (semantic similarity) → stories (interpreted clusters) → narratives (patterns across stories over time).

Building on the identified gaps in the global, long-term, and multilingual analyses of media narratives, this study addresses the following research questions. These questions structure the empirical analysis while operationalising the theoretical framework by jointly examining issue salience, narrative circulation, temporal evolution, the role of key actors, and linguistic differences.

### Research questions

What were the dominant media narratives concerning Russia and Ukraine between 2013 and 2024?

In particular:

1. How has media coverage related to Russia and Ukraine evolved from 2013 to 2024?
2. Which individuals (person-level named entities) were most frequently mentioned in the media coverage overall and at different points in time, and how did their prominence and contextualisation in coverage evolve throughout the analysed period?
3. How has the language distribution of articles from Russia-linked media evolved over time, and how does it relate to differences in coverage across language groups?

Addressing these questions allows us to demonstrate the value of hybrid human-AI approaches in tracing the media discourse on Russia and Ukraine across countries.

## Materials and methods

**Fig 1:**
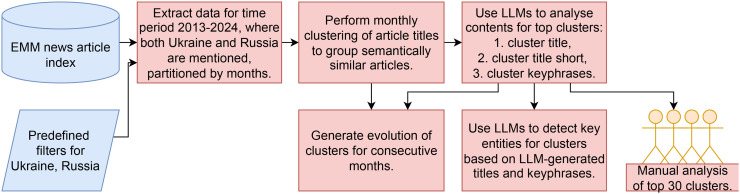
Human–AI analytical pipeline. From data extraction via the Europe Media Monitor (EMM) to human annotation.

Our hybrid human-AI analytical pipeline (see Fig 1) relied on available AI models and established Natural Language Processing techniques. Due to the volume of data and the complexity of the task, we used several models, ranging from those with fewer parameters (and thus more efficient) for the pre-processing tasks to those with more parameters (and thus more capable) for the later stages requiring more intense reasoning.

The pipeline began with a keyphrase-based filtering step to construct the dataset, which was then divided into monthly batches. For each batch, clustering was performed using an efficient AI model in combination with a series of graph-processing operations, resulting in a large set of clusters of stories per month. A larger, more capable model was then used to summarise the contents of the top clusters and generate keyphrases and short summaries. Finally, human analysts annotated the results, interpreting clusters and identifying the main narratives, which were subsequently synthesised into monthly and yearly summaries.

### Dataset

A dataset consisting of 22,197,751 article titles from 14,622 sources operating in 194 countries, covering the period from 1 January 2013 to 31 December 2024, was retrieved from the Europe Media Monitor (EMM) [21]. The articles span up to 74 languages across the dataset, with 74 languages observed in 2024. More details for each analysed year are provided in S1 Table of the supporting information.

As EMM collects news articles from a manually curated and continuously updated list of online media domains worldwide, developed and maintained by the Joint Research Centre (JRC) in collaboration with domain experts and media analysts, the number of monitored sources grew over the 12-year period, exceeding 25,000 in 2024. This expansion reflects the progressive broadening of geographic and linguistic coverage within the monitoring system.

For the analysis of language distribution in Russian media (RQ3), “Russian sources” were defined based on the country-of-origin classification provided by EMM. Each outlet in EMM is assigned a country label reflecting its origin, which is automatically associated with each article during data processing. Consequently, in the whole analysis by “Russian sources” we consider sources classified by EMM as Russian. Their number increased from 101 in January 2013 to 341 in December 2024, in line with the expansion of the monitoring system. The full list of sources classified as Russian is publicly available in the JRC Data Catalogue [22] entry associated with this study.

A multilingual query was applied to extract all article titles mentioning both Russia and Ukraine. In addition, EMM provides metadata, including geolocation identifiers for countries, regions, and cities. All matching articles were retained without any additional relevance-based filtering or exclusion, in order to capture the full spectrum of reporting in which both countries were mentioned. The resulting dataset contains article titles for the entire period and, for articles published after July 2019, also includes short snippets automatically extracted from the article text. S1 Table of the supporting information provides a yearly breakdown of dataset characteristics, including the number of articles, sources, languages, and countries.

### Clustering of articles

To group semantically similar articles and identify recurring patterns in media coverage, multilingual clustering was carried out based on article titles for each month using a combination of advanced techniques. Building on established methodologies [23], we provided extensions incorporating sentence embeddings from language models to capture semantic differences across languages.

The method was implemented in Python. The LaBSE model [24] was employed to create embedding vectors for each text, while PyNNDescent [25] was used to construct an approximate neighbourhood for the embedded texts. We set the parameter for the number of nearest neighbours to be searched to *k = 100*. The resulting neighbourhood matrix enabled the construction of a graph, where nodes representing similar texts were connected by edges.

Finally, the library LeidenAlg [26] was used to detect communities within this graph. We retained only communities with at least 10 articles to focus on the most widespread patterns in media coverage. The resolution parameter *β*, which determines the granularity of clusters, was set to *β =* 1.0, and “RBConfigurationVertexPartition” was chosen as a partition type. The resolution parameter was determined through manual analysis of a subset of the data (one month), resulting in the adoption of generic clusters due to the substantial data volume. Nonetheless, this parameter can be adjusted to a higher value to obtain more detailed clusters, depending on the analytical objective. The clustering method based on community detection in the neighbourhood graph was preferred because it does not require specifying the expected number of resulting clusters.

### Summarisation and keyphrases

After the articles were clustered, we limited our scope to the largest clusters in terms of article count to focus on the most widespread news each month. Given the nature of the clustering algorithm, articles within the same cluster were expected to share similar content. This approach optimised computational resources and reduced token usage for subsequent analysis.

For each cluster, a random sample of 100 articles was selected using the Pandas sampling method with default parameters. For articles published after July 2019, we used snippets consisting of the first 350 characters to capture the lead paragraph and core context while maintaining computational efficiency; for earlier data, we used article titles. Using GPT-4, GPT-4-turbo, and GPT-3.5-turbo (depending on availability of GPU resources) [27], we generated for each cluster a title, a shortened title (maximum two words) for visualisation purposes, and five keyphrases summarising the content of the cluster.

Although the input data were multilingual, all model outputs (including cluster titles and keyphrases) were generated in English to ensure consistency and facilitate subsequent analysis. Details on model settings, prompt structures, and generation parameters are provided in S3 Table of the supporting information.

Since employing a single model was impractical due to computational constraints, we manually reviewed and compared outputs from multiple models on a sample month of data. GPT-4 and GPT-4-turbo delivered consistent quality, while GPT-3.5-turbo occasionally deviated from the required structure; such cases were re-evaluated using GPT-4. Our assessment indicated that models earlier than GPT-3.5-turbo were unsuitable for this task. To ensure consistency, we processed the top 30 clusters using GPT-4 or GPT-4-turbo across all months.

### Output

To present the data to human analysts, the output of the analysis was formatted as a comma-separated values (CSV) file comprising, for each cluster, a title, a shortened title, five keyphrases, and the number of articles in the cluster. The full dataset, including the outputs of the cluster-linking procedure described below, has been made publicly available through the JRC Data Catalogue [22].

### Human annotation

Human annotation constituted a key step in the interpretation of the clustering results. The manual analysis was conducted in English, as all outputs generated by the language models were provided in English. All analysts involved in the annotation process were fluent in English. When uncertainty arose regarding the content described by the model, analysts consulted the original source texts in their original languages. Four analysts reviewed the clustered outputs, focusing on the top 30 most prominent clusters each month. A manual assessment showed that this threshold was sufficient to capture the main narratives and their relative prevalence, as clusters beyond this point represented less prominent patterns.

For each cluster, annotators examined the LLM-generated title and keyphrases, which summarised the content of the cluster, and assigned a descriptive label capturing the main pattern in how the issue was described. As part of this process, qualitative checks of cluster coherence were carried out by verifying that the generated titles and keyphrases were consistent with the underlying article titles and, where available, snippets within each cluster. To increase transparency, the supporting information includes illustrative examples of randomly selected clusters whose sampled headlines demonstrate internally coherent topical groupings across sources and languages.

Clusters assigned the same label were grouped together and interpreted as representing a common story, understood as a coherent and recurring way in which issues are described and linked in media coverage. Narratives were then identified as broader patterns emerging across multiple related stories, based on the most frequently recurring labels within each month. These were synthesised into monthly summaries, which were subsequently used to derive annual narrative patterns.

To ensure consistency, the analysts jointly developed and refined the annotation procedure at the outset and co-annotated a subset of the data to align their interpretation of the task and the application of the annotation rules. Following this calibration phase, annotation was conducted independently. During the annotation process, analysts recorded their labels and grouped clusters into preliminary narratives, and regularly discussed these in joint meetings to resolve ambiguities and maintain a consistent interpretation.

Each analyst drafted the monthly summary corresponding to the clusters they annotated. To ensure coherence and comparability across the full time series, all monthly summaries were subsequently reviewed and harmonised by a single analyst, who ensured consistent terminology, structure, and interpretation across months and synthesised the results into annual summaries.

### Cluster linking

We tracked the evolution of clusters across consecutive time periods to analyse how patterns in coverage develop over time. While clustering captures recurring patterns in coverage at a given point in time, cluster linking enables the analysis of how these patterns persist, transform, or recombine over time by identifying connections between related clusters across consecutive periods.

We achieved this by measuring the pairwise similarity of clusters between two consecutive time periods. The approach was implemented in Python and used the embedding vectors of article titles obtained from the previous clustering step as input. The position of each cluster in the embedding space was determined by the article locations. We focused on a subset consisting of five per cent of clusters per month.

Additionally, for each month, we applied the PaCMAP [28] reduction model to obtain a two-dimensional (2D) representation of the selected clusters. To evaluate the similarity between clusters C1 and C2 from two consecutive time periods, we performed Gaussian extrapolation for the location of C1 in the 2D reduced space, normalising the results to a [0, 1] scale. This method provided, for each point in the 2D space, the probability that cluster C1 was situated at that specific location. The function $F_{C\text{1}}(x,y)\;=\;p_{xy}$ was then used to take the coordinates of a point (x, y) in the 2D space and return the probability that (x, y) belongs to cluster C1. We then used this function to calculate the average score for the embeddings of articles from cluster C2, reduced into 2D space. The resulting value represented the similarity between clusters C1 and C2:


$$\begin{array}{c} sym_{C\text{1},C\text{2}}\;=\;\frac{\text{1}}{N_{\text{2}}}\sum t\in C\text{2}\;F_{C\text{1}}\left(t_{x},\;t_{y}\right),\;\;\;\;\;\;\;\;|C\text{2}|\;=\;N_{\text{2}} \end{array}$$

In the context of our analytical framework, cluster linking is used to examine the temporal evolution of narratives identified through the annotation process. Following the identification of the most prominent narratives in the monthly and yearly analyses, we manually selected the clusters associated with these narratives and tracked their evolution over time using the cluster-linking procedure. Rather than analysing all clusters, this approach focuses on those that contribute to the identified narratives, enabling us to trace how related clusters, interpreted as stories, persist, merge, or fragment across consecutive periods. In this way, cluster linking supports the analysis of narratives as temporally evolving patterns that emerge across multiple related stories.

### Analysis

The analysis consisted of five components:

(i) tracking media coverage over time;
(ii) interpreting clustered outputs through human annotation to identify stories and narratives;
(iii) analysing the temporal evolution of these narratives using cluster linking;
(iv) analysing the prominence and contextualisation of the most frequently mentioned entities in the dataset; and
(v) assessing the language distribution of articles published by Russian sources.

For component (i), we measured monthly article counts and normalised them by the number of active sources per month to account for changes in the monitored sources. Specifically, the average number of articles published per month per source was multiplied by the maximum number of active sources over the entire study period. Peaks in coverage were then cross-checked against major geopolitical events to assess their significance.

For component (ii), the top 30 clusters per month were annotated by four analysts, following the procedure described above. Drawing on these annotations, clusters were interpreted as stories and grouped into broader narratives, which were synthesised into monthly summaries (see supporting information). These monthly summaries then served as the basis for the annual summaries (see Results section).

For component (iii), we identified the most prominent narratives for each year based on the annual summaries derived from the annotation process. Clusters associated with these narratives were manually selected and tracked over time using the cluster-linking algorithm described above. This approach enabled us to trace how related clusters, interpreted as stories, persisted, merged, or fragmented over time, thereby capturing the temporal dynamics of narratives as patterns emerging across multiple related stories. Interactive visualisations of the complete evolution are available in the JRC Data Catalogue [22]. Two periods corresponding to key geopolitical events were selected for detailed analysis: Russia’s annexation of Crimea (2013–2014) and Russia’s full-scale invasion of Ukraine (2021–2022).

For component (iv), we analysed the prominence and contextualisation of the most frequently mentioned individuals in the dataset by examining the monthly top clusters. Names were extracted automatically from the original language using LLMs. The prompts required answers in English and in base form. The outputs were then post-processed and standardised using cosine similarity to account for spelling or form variations. The most salient individuals were then ranked according to their frequency of occurrence across these clusters, producing a list of political leaders and public figures who dominated the most widely covered clusters in the media over the analysed period. To ensure robustness, we compared our results with the EMM entity extraction pipeline (available for articles after July 2019), which provided a baseline for validation. The two approaches yielded highly overlapping lists of key figures, increasing confidence in the results.

To analyse the contextualisation of these individuals in coverage, we examined how mentions of these individuals evolved over time in relation to the monthly summaries derived from the clustered and annotated data (see supporting information). Periods of increased or sustained prominence were analysed in conjunction with the corresponding monthly summaries, and, where necessary, cross-checked against the annotated cluster outputs. This approach was applied consistently across all analysed key figures, allowing us to identify recurring patterns in how individuals were described and linked to specific developments.

For component (v), we assessed the language distribution of articles published by Russian sources. For each month, the number of articles was aggregated by publication language and expressed as a percentage of the total output. This allowed us to identify the languages most frequently used, track how their relative share evolved over time, and examine differences in coverage across language groups.

These components correspond directly to the research questions: component (i) addresses the evolution of media coverage over time (RQ1); components (ii) and (iii) identify and trace the development of media narratives (RQ1); component (iv) examines the prominence and contextualisation of key individuals (RQ2); and component (v) analyses the language distribution of Russia-linked media and its relation to differences in coverage across language groups (RQ3).

## Results

This section presents the findings of our analysis of media coverage related to Russia and Ukraine from 2013 to 2024, guided by the main research question: what were the dominant media narratives concerning Russia and Ukraine between 2013 and 2024?

The analysis addresses three research sub-questions: (1) how media coverage evolved over time; (2) which individuals were most frequently mentioned and how their prominence and contextualisation changed; and (3) how the language distribution of Russia-linked media evolved over time.

Throughout the study, we employ different terms to describe the evolving situation, reflecting both the historical context and prevailing terminology of the time. These terms are drawn from the AI-generated cluster titles, which in turn are based on the headlines of the underlying news articles; as such, they mirror the language used by media outlets at each point in time and serve a descriptive labelling function only, without influencing the underlying computational procedures. Following 24 February 2022, we consistently refer to the events as “Russia’s war of aggression against Ukraine” or “Russia’s full-scale invasion of Ukraine”. These formulations reflect terminology widely used in international political discourse and in the media coverage analysed, including references grounded in international legal characterisations of the conflict [29]. Prior to 24 February 2022, the terminology used in media coverage was less uniform. Terms such as “conflict,” particularly concerning Crimea and the Donbas region, as well as “tensions,” “crisis,” and “Russo-Ukrainian relations,” were commonly employed. Our use of these terms is not intended to downplay the severity of the situation but rather to reflect the language prevalent in media narratives during that period, acknowledging the absence of a unified and precise terminology before the escalation in 2022.

### Media coverage over time

The analysis of media coverage over time addresses Research Question 1, which examines how coverage related to Russia and Ukraine evolved from 2013 to 2024. Across the analysed period, three main patterns emerge: first, sharp peaks in coverage correspond to major geopolitical events; second, these peaks are often followed by periods of sustained elevated attention; and third, attention subsequently declines but remains responsive to new developments.

**Fig 2:**
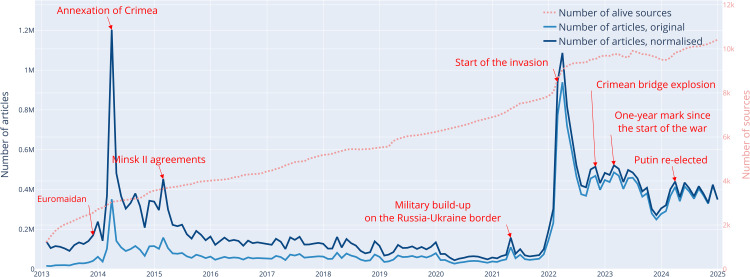
Distribution of articles from 2013 to 2024. The light blue line represents the total number of articles aggregated per month; the dark blue line illustrates the number of articles aggregated per month normalised by the number of active sources; and the pink dotted line shows the increase in monitored sources. The interactive HTML version of the figure is available in the dataset repository [22].

These patterns can be observed in the temporal distribution of article volumes (see Fig 2). The data show significant fluctuations in volume, with several notable peaks corresponding to specific newsworthy events. Normalised values were obtained by aggregating the overall volume of monitored sources per month; this approach accounts for the substantial increase in monitored sources, from 1,220 in January 2013 to 10,417 in December 2024.

Media coverage remained relatively low until November 2013, when the Ukrainian government’s suspension of the EU Association Agreement, a political and trade agreement between Ukraine and the EU, sparked mass protests in Ukraine. The Euromaidan protests — “Euro” refers to the protests’ focus on closer integration with Europe and “Maidan” is part of the name of the central square in Kyiv where the protests began — rapidly grew into a large-scale pro-European movement. Media outlets across the globe reported on the Euromaidan protests, portraying them as a struggle for democratic reforms, closer ties with Europe, and independence from Russian influence.

Media coverage increased from two hundred thousand monthly articles at the end of 2013 to around 1.2 million in February and March 2014 when Russia invaded the Crimean Peninsula, part of Ukraine, and then attempted to annex it through an illegal and illegitimate referendum [30], held under military occupation. The international community responded with widespread condemnation; the EU and the US imposed sanctions on Russian officials and companies in response to what was widely regarded as a violation of Ukrainian sovereignty.

Following the illegal annexation of Crimea, coverage decreased considerably until February 2015, when the Minsk II Agreement led to a significant peak, with five hundred thousand monthly articles describing negotiations between representatives of Russia, Ukraine, the Organisation for Security and Cooperation in Europe (OSCE), and the pro-Russian separatist regions of Donetsk and Luhansk. The accord, aimed at halting hostilities, included measures such as a ceasefire, withdrawal of heavy weaponry, and constitutional reforms in Ukraine. However, reports highlighted persistent violations almost immediately after the signing of the agreement.

Following the Minsk II Agreement, coverage of the Russo-Ukrainian conflict consistently decreased in the following years, falling below eighty thousand monthly articles in 2020. Volume rose again in April 2021, reaching two hundred thousand monthly articles, when Russia began amassing troops along the Russia–Ukraine border, raising concerns about a potential large-scale war.

At the start of 2022, as Russia continued to intensify its military presence near Ukraine, media coverage quickly increased, escalating dramatically when Russia launched its full-scale invasion of Ukraine on 24 February 2022. In March 2022, a peak of 1.1 million articles was registered, with most coverage focusing on the Russian military offensive, including the advance towards Kyiv and the assault on cities such as Mariupol.

Following the full-scale invasion of Ukraine, coverage fell to a relatively high level of approximately four hundred thousand monthly articles throughout the remainder of 2022 and the subsequent two years. Notable surges in coverage were observed in response to military and geopolitical events. For instance, the peak in October 2022 was associated with the explosion on the Kerch Strait Bridge in Crimea; the spike in February 2023 corresponded with the one-year mark since the start of Russia’s war against Ukraine; and the peak in March 2024 was associated with the re-election of President Vladimir Putin. Further events are detailed in S2 Table in the supporting information.

Overall, the temporal distribution of coverage shows that media attention was strongly event-driven, with major geopolitical developments triggering sharp increases in volume, followed by periods of sustained but gradually declining attention.

### Year-by-year analysis

The year-by-year analysis addresses the main research question by identifying the dominant media narrative patterns in each year from 2013 to 2024, based on the clustered and annotated data. Across the analysed period, several recurring narrative patterns emerge, including geopolitical tensions, energy politics, sanctions, and disinformation, whose prominence shifts over time in response to major geopolitical developments. While specific events shape short-term shifts in coverage, these broader patterns provide continuity in how issues are described and contextualised over time.

Table 1 provides a synthesis of the dominant media narratives and associated key developments for each year, presenting the four most prominent narratives identified through manual annotation and offering a comparative overview of how these patterns recur and evolve across the analysed period.

**Table 1:** Overview of dominant media narrative patterns and associated key developments by year (2013–2024). The four narrative patterns shown for each year correspond to the most prominent narratives identified through the manual annotation process described in the ‘Human annotation’ section. Narratives are colour-coded to reflect recurring narrative patterns across the analysed period (blue: geopolitical tensions and security concerns; orange: energy politics and sanctions; pink: political and societal dynamics; brown: disinformation and information dynamics).

Year	Key developments	Narrative 1	Narrative 2	Narrative 3	Narrative 4
2013	EU Association Agreement suspended; Euromaidan protests	Geopolitical tensions	Energy dependency and disputes	Domestic governance and political unrest	Economic instability
2014	Annexation of Crimea; MH17; conflict in Donbas	Annexation of Crimea and its implications	Armed conflict in eastern Ukraine	Geopolitical and economic consequences	Ukrainian national identity
2015	Minsk II Agreement; continued hostilities	Minsk Agreements and diplomatic stalemate	Economic sanctions and repercussions	Energy politics	Hybrid warfare and regional security dynamics
2016	Continued conflict; cyber incidents	Conflict and diplomatic stalemate	Energy politics	Cybersecurity and Foreign Information Manipulation and Interference (FIMI)	Human rights concerns
2017	Ongoing conflict; sanctions expansion	Ongoing conflict and Russian aggression	Sanctions and diplomatic tensions	Hybrid warfare (including cyber threats)	Cultural and political dimensions
2018	Kerch Strait incident; Skripal case	Escalation and regional instability	Energy politics and sanctions	Disinformation and FIMI	Religious dynamics
2019	Zelenskyy elected; ongoing conflict	Conflict and security dynamics	Energy politics	Political transition and leadership dynamics	Disinformation campaigns
2020	COVID-19; stalled diplomacy	Conflict and military tensions	Energy politics and sanctions	Disinformation and diplomatic challenges	Governance and corruption issues
2021	Russian troop build-up; diplomacy	Escalating military tensions	Energy politics	Diplomatic efforts and negotiations	Disinformation narratives
2022	Full-scale invasion of Ukraine	Full-scale invasion and war dynamics	Disinformation and FIMI	Geopolitical realignment	Energy security and crisis
2023	NATO expansion; Kakhovka dam	War dynamics and geopolitical realignment	Energy and economic security	Disinformation and platform dynamics	Governance challenges
2024	Continued war; NATO/EU developments	War dynamics and military developments	Geopolitical realignment	Diplomatic and economic measures	Disinformation and information warfare

The table highlights how these recurring narrative patterns manifest across years. Geopolitical tensions and security concerns consistently structure coverage, from early reporting on Ukraine’s positioning between competing spheres of influence to later emphases on military escalation and alliance dynamics. Energy politics and sanctions recur as prominent narratives, particularly during periods of heightened conflict, such as the annexation of Crimea in 2014 and the full-scale invasion in 2022, when economic measures and energy dependencies become central. Disinformation-related narratives, while present earlier, become more prominent from around 2016 onwards and especially after 2022, reflecting a growing focus on the informational dimension of the conflict and the role of foreign information manipulation and interference.

The following section elaborates on these patterns, illustrating how they are reflected in the clustered and annotated data and providing additional context on their evolution across the analysed period.

#### 2013.

Coverage consistently emphasised **geopolitical tensions**, with reporting portraying Ukraine as situated between competing spheres of influence. Articles repeatedly described Ukraine’s prospective EU Association Agreement as a decisive shift toward Europe, while presenting Russia’s response in terms of economic pressure, trade restrictions, and geopolitical competition. By November, the Ukrainian government’s abrupt decision to suspend the EU deal, reported as occurring under Russian pressure, was widely reported as a turning point, triggering large-scale protests. International outlets portrayed the “Euromaidan” protests as both a demand for European integration and a rejection of Russian interference, casting Ukraine as a central site of contestation between Western integration and Russian influence.

**Energy dependency** emerged as a persistent narrative strand, reflecting Ukraine’s vulnerability to Russian leverage through natural gas supplies. Reports detailed ongoing disputes between Kyiv and Moscow over pricing and debt, with Russia’s Gazprom repeatedly using its dominant position to influence Ukraine’s policies. Media also covered Ukraine’s efforts to diversify energy supplies, but these efforts were described as insufficient to break free from Russian control. Energy security became intertwined with broader geopolitical stakes, as Russia’s tactics were described as part of a broader strategy to maintain dominance over its neighbour.

**Domestic governance and public discontent** gained attention, particularly as the year progressed. International media highlighted widespread corruption, political repression, and the controversial imprisonment of opposition leader Yulia Tymoshenko, casting doubt on the state of Ukraine’s democracy. These articles often positioned Ukraine’s internal struggles within the broader narrative of its geopolitical alignment, portraying the Yanukovych administration as complicit in perpetuating Russian interests. The eruption of the Euromaidan protests in late 2013 shifted global focus toward the growing divide between Ukraine’s government and its people, with reports portraying the movement as a pivotal moment in Ukraine’s quest for independence and democracy.

Other noteworthy narratives included **concerns about Ukraine’s economic stability** amid deteriorating trade relations and mounting debt and **cultural and religious dynamics**, as Russia leveraged historical and spiritual ties to assert influence.

#### 2014.

The illegal **annexation of Crimea** in March 2014 was the defining event of the year, generating extensive media coverage. Reports highlighted Russia’s military presence in the peninsula, the controversial referendum organised under Russian control, and Ukraine’s diplomatic efforts to challenge Moscow’s actions. Coverage widely described the annexation as a move by the Kremlin to assert dominance over Ukraine and prevent its shift towards European integration, often presenting it as a violation of international law.

**Russia’s undeclared war against Ukraine** became a central focus of media coverage, with coverage describing the emergence of pro-Russian separatist movements in Donetsk and Luhansk, the Ukrainian government’s military response, and Russia’s support for the insurgents. Reports highlighted the human cost of the conflict, including civilian casualties and a growing humanitarian crisis. The downing of Malaysia Airlines Flight MH17 in July 2014 marked a turning point, with coverage widely attributing responsibility to Russian-backed separatist forces, while alternative explanations also circulated [31]. The incident intensified scrutiny on Russia’s role in the conflict and strengthened calls for international action against Moscow.

The **geopolitical and economic fallout of the crisis** was another dominant narrative strand, with media closely covering the imposition of Western sanctions on Russia and their impact on global diplomacy. Reports analysed the EU and US efforts to isolate Russia economically, as well as the Kremlin’s countermeasures including trade restrictions and energy supply threats. The conflict was frequently described as a key moment in the post-Cold War order, with Ukraine symbolising the broader struggle between Western democratic values and Russian authoritarianism. Energy politics remained central to the discourse, with Gazprom’s pricing disputes and Ukraine’s gas dependency reinforcing concerns over Europe’s energy security.

Emerging narratives included the **impact of the crisis on Ukrainian national identity**, with increasing media attention on the country’s efforts to solidify its independence from Russian influence. Reports also highlighted the role of **Foreign Information Manipulation and Interference (FIMI)**, with competing narratives shaping public perceptions in Ukraine, Russia, and the West [32].

#### 2015.

The **Minsk Agreements**, two ceasefire agreements aimed at resolving the conflict in eastern Ukraine signed on 5 September 2014 and 12 February 2015, respectively, were a recurring focus of coverage throughout the year. Across articles, the agreements were consistently described in terms of repeated ceasefire violations, diplomatic stalemates, and a worsening humanitarian crisis. This pattern of reporting linked ongoing diplomatic efforts to limited progress on the ground, with millions displaced and access to humanitarian aid restricted. The one-year mark of Russia’s illegal occupation and attempted illegal annexation of Crimea (hereafter referred to for brevity as “Russia’s annexation of Crimea”) and the investigation into the downing of MH17 kept international attention focused on Ukraine.

**Economic and geopolitical repercussions** were another dominant narrative strand, particularly the sanctions against Russia. Media coverage examined the economic impact of these measures on Russia and Moscow’s retaliatory actions, including food embargoes and energy supply threats. Energy politics were central to the discourse, with disputes over gas supplies between Russia and Ukraine affecting European energy security. The media also explored the implications of proposals to exclude Russia’s exclusion from the SWIFT payment system.

**Hybrid warfare and regional security tensions** featured prominently, with media reports on Russia’s use of propaganda and cyber tactics as part of its strategic approach. NATO’s responses, including military exercises and increased presence in Eastern Europe, were described as efforts to counter perceived Russian aggression. The intersection of the Ukraine crisis with Russia’s military involvement in Syria complicated the geopolitical landscape, prompting discussions on NATO’s strategic priorities and challenges in addressing multiple regional conflicts.

#### 2016.

Coverage throughout the year persistently described **conflict and diplomatic stalemates**, emphasising the Minsk Agreements and other diplomatic efforts, such as the Normandy summit. While the release of Ukrainian pilot Nadiya Savchenko was widely reported as a symbolic victory, the persistent ceasefire violations highlighted the deep mistrust.

**Energy politics** remained a central element of coverage, reflecting the intricate relationship between economic interests and geopolitical strategy. Ukraine’s efforts to secure energy independence and the contentious debates surrounding pipeline projects, such as Nord Stream 2 and TurkStream, underscored the strategic importance of energy transit routes. The construction of the Kerch Strait Bridge, connecting Russia to Crimea, consolidated Russia’s control over the illegally occupied and annexed peninsula, drawing international criticism. Meanwhile, Western sanctions against Russia were extended, and discussions about their impact and future persisted, revealing divisions within the EU regarding how to address Russian actions.

An increasingly prominent narrative was the focus on **cybersecurity and FIMI**. Reports of cyberattacks on Ukrainian infrastructure, coupled with allegations of Russian interference in the US presidential election, emphasised the growing role of hybrid warfare tactics. Media coverage highlighted the strategic use of cyber operations and disinformation campaigns, characterising them as critical components of Russia’s approach to asserting influence and destabilising opponents. This narrative underscored the broader security challenges facing Ukraine and Western nations in countering these threats.

Other narratives included the international community’s **concern about human rights abuses** in illegally occupied and annexed Crimea (hereafter referred to for brevity as “Crimea”) as well as the **cultural and historical dimensions of the conflict**, exemplified by Ukraine’s Eurovision entry, a song about Stalin-era deportations.

#### 2017.

The year was marked by **Russia’s persistent aggression** in the eastern and southern regions of Ukraine, with the Minsk Agreements remaining a focal point despite recurrent violations and diplomatic stalemates. Coverage consistently highlighted intense fighting around areas such as Avdiivka, the humanitarian crisis, and the deep-rooted mistrust between the conflicting parties. While discussions around a potential United Nations (UN) peacekeeping mission gained some traction, no tangible progress was made. Coverage also emphasised the withdrawal of Russian officers from the Joint Centre for Control and Coordination (JCCC) as further straining ceasefire efforts.

**Sanctions and diplomatic tensions** dominated the geopolitical landscape. The extension of Western sanctions against Russia reflected the international community’s response to Russia’s actions. The US sanctions package introduced in July, despite President Trump’s initial reluctance, underscored growing Western resolve. Meanwhile, Russia attempted to bypass these sanctions through intermediaries and alternative arrangements. Diplomatic interactions, such as Merkel’s firm stance during her 2017 visit to Sochi, where she discussed the ongoing conflict in eastern Ukraine and emphasised the need for progress on the Minsk Agreements, illustrated divisions within the EU and the broader international community.

A prominent narrative concerned **hybrid warfare**, particularly cybersecurity threats. High-profile cyberattacks, such as the WannaCry and Petya ransomware incidents, which had a severe impact on Ukraine, were described as part of Russia’s strategy to destabilise and exert influence. Allegations of Russian interference in electoral processes in the US and Europe, alongside NATO’s countermeasures, reflected growing recognition of the cybersecurity challenges facing Western nations and Ukraine alike.

Other noteworthy narratives included the **cultural and political dimensions of the conflict**, as seen in Ukraine’s barring of a Russian participant from the Eurovision song contest due to visits to Crimea.

#### 2018.

Coverage consistently emphasised **violent incidents**, such as the Kerch Strait naval clash in November, presenting them as indicators of escalating regional instability. Coverage also underscored the imposition of martial law in Ukraine as reflecting the gravity of the situation, while the assassination of Alexander Zakharchenko, the leader of the self-proclaimed Donetsk People’s Republic (DPR), and the Skripal poisoning case further complicated diplomatic relations. Reporting presented these events as emblematic of the challenges in achieving lasting peace and the broader geopolitical struggle around the regional conflict.

**Energy politics and economic sanctions** remained central to the narrative, reflecting the intricate relationship between geopolitical strategy and economic interests. The Nord Stream 2 gas pipeline project was a particularly contentious issue, with strong opposition from Ukraine, the US, and Poland, which warned of increased European dependence on Russian gas. Despite diverging views within the EU, sanctions against Russia were extended, and new measures were explored in response to Russia’s armed aggression against Ukraine. The construction and symbolic significance of infrastructure projects like the Crimean Bridge further illustrated Russia’s consolidation of control over annexed territories, drawing international criticism and reinforcing the sanctions narrative.

Media coverage in 2018 increasingly focused on **FIMI.** The MH17 tragedy and associated legal actions against Russia underscored the ongoing struggle for accountability amid disinformation campaigns. These developments were described as part of broader efforts to counter Russian hybrid threats and support Ukraine’s quest for sovereignty and justice.

Finally, significant attention was given to the **religious dimension of the conflict**. Following the schism between the Russian Orthodox Church and the Ecumenical Patriarchate over Ukrainian autocephaly in October 2018, media outlets described Ukraine’s religious independence as a significant step in asserting sovereignty and resisting Russian influence.

#### 2019.

Media coverage described the volatile dynamics of the **international armed conflict** (hereafter referred to for brevity as “conflict”), paying significant attention to the situation in eastern Ukraine and Crimea. The Kerch Strait incident from late 2018 continued to reverberate, with discussions on international legal proceedings and geopolitical implications. Military engagements in Donbas, along with the introduction of simplified Russian citizenship procedures for its residents, highlighted persistent instability and humanitarian concerns in the region. NATO’s increased presence in the Black Sea and Ukraine’s military modernisation efforts reflected the broader security concerns.

**Energy politics and economic issues** were pivotal in shaping coverage. The Nord Stream 2 pipeline project was a major point of contention. Moreover, the contaminated oil in the Druzhba pipeline and negotiations around gas transit agreements between Russia and Ukraine emphasised the critical role of energy security in the region. Ukraine’s efforts to reduce reliance on Russian gas, including the establishment of an independent gas transmission system operator, were presented as crucial steps towards energy independence.

2019 also saw significant developments in **political dynamics and disinformation campaigns**. Coverage consistently described Volodymyr Zelenskyy’s election as Ukraine’s President as a substantial political shift, with his administration’s approach to Russia and peace negotiations in eastern Ukraine being closely scrutinised. Meanwhile, Russian disinformation campaigns continued to target Ukraine, with narratives framing it as the aggressor. These efforts were described as part of a broader strategy to undermine Ukraine’s international reputation.

Finally, the **religious dimension** of the conflict persisted as a significant narrative. The autocephaly of the Ukrainian Orthodox Church, which began in 2018, continued to be a symbol of Ukraine’s assertion of sovereignty and resistance to Russian influence. This development was framed within the broader geopolitical context, highlighting the intersection of religion and national identity.

#### 2020.

The **conflict in eastern Ukraine and Russia’s annexation of Crimea** remained a central focus, with military engagements and ceasefire violations continuing to destabilise the region. Ukrainian efforts to modernise the military and enhance naval capabilities were described as necessary responses to Russian aggression, while Moscow described these actions as provocative. NATO’s presence in the Black Sea and joint military exercises with Ukraine underscored the international dimension of the conflict, drawing contrasting reactions from Ukrainian and Russian officials.

**Energy security and economic sanctions** formed central strands of coverage throughout the year. The Nord Stream 2 pipeline continued to spark debates on European reliance on Russian energy and Ukraine’s role as a transit country. Coverage reflected differing interpretations of sanctions, with some accounts emphasising their strategic importance in countering Russian actions, while others described them as politically motivated and ineffective.

**Disinformation campaigns and diplomatic challenges** featured as prominent narratives. Russian efforts to influence perceptions through disinformation campaigns aimed at undermining Ukraine’s sovereignty and Western support were widely reported. Diplomatic efforts, including the Normandy Format negotiations involving France, Germany, Ukraine, and Russia, as well as discussions within the Trilateral Contact Group, a diplomatic body facilitating dialogue between Ukraine, Russia, and the Organisation for Security and Cooperation in Europe (OSCE), achieved limited progress in resolving the Donbas conflict, with entrenched narratives and mutual accusations hindering meaningful advancement.

Emergent narratives identified in the 2020 dataset included **Ukraine’s struggles with corruption, governance, and the influence of oligarchs**. Media coverage examined various political controversies, including leaked audio recordings that suggested potential concessions to Russia and raised suspicions of foreign influence on Ukraine. These issues were compounded by the COVID-19 pandemic, which added complexity to governance and public health responses.

#### 2021.

Coverage throughout 2021 consistently described escalating **military tensions**, with repeated emphasis on Russian troop build-ups near Ukraine and the risk of a broader invasion. Across articles, military developments were linked to ongoing ceasefire violations in the Donbas region and presented as indicators of a deteriorating security environment. Reports frequently connected military activity to diplomatic efforts to de-escalate the situation, including high-level talks between US President Joe Biden and Russian President Vladimir Putin, highlighting the limited impact of negotiations in reducing tensions.

**Energy politics** remained central, with the Nord Stream 2 pipeline continuing to spark intense debate. The media emphasised the pipeline’s potential to weaken Ukraine’s role as a transit country and increase European dependency on Russian gas. The gas transit dispute added to the volatility in European energy markets, as Russia reduced gas flows through Ukrainian pipelines. This prompted discussions on energy diversification and the strategic implications of Europe’s reliance on Russian energy. The US-Germany agreement on Nord Stream 2 aimed to address some concerns, but Ukrainian officials remained wary of the long-term economic and security consequences.

**Diplomatic efforts** to resolve the conflict, such as the Normandy Format negotiations, were frequently described as yielding limited results. Espionage and cyberattacks linked to Russia targeted Ukrainian institutions, intensifying the hybrid warfare dimension of the conflict. The MH17 trial continued to unfold, with significant legal developments implicating Russian-backed separatists, further straining Russia’s relations with the Netherlands and other Western countries.

**Disinformation campaigns** persisted, with Russia framing NATO and Western actions as provocations, while Ukraine and Western countries emphasised the need for stronger security assurances for Ukraine and sanctions against Russia.

#### 2022.

In 2022, coverage shifted toward **Russia’s full-scale invasion of Ukraine**, focusing on the military advances and the severe humanitarian crisis that ensued, particularly in cities such as Kharkiv and Mariupol, while also emphasising the resilience of Ukrainian forces. Reporting repeatedly linked military operations to large-scale displacement, urban destruction, and risks to critical infrastructure, with fighting near the Zaporizhzhia nuclear power plant raising fears of a potential nuclear disaster. Across sources, Russia’s invasion of Ukraine was presented as both a military and humanitarian crisis, with continued emphasis on failed diplomatic efforts to de-escalate the situation.

**FIMI** emerged as a central pattern in coverage, with articles describing the use of disinformation and competing representations of the invasion. The term “special military operation” appeared in reporting on the war, alongside coverage that challenged such characterisations and emphasised the scale of the conflict. Reporting highlighted the presence of misleading claims and the efforts to contest them, underscoring the informational dimension of the war.

**Geopolitical repercussions** were significant, with Russia’s full-scale invasion of Ukraine prompting realignments and strategic considerations. Discussions in Sweden and Finland about NATO membership reflected growing security concerns in Europe. The strengthening of alliances between Russia and countries such as Iran and China was frequently described as a counterbalance to Western influence.

Finally, **energy politics** remained central, as Europe grappled with its dependency on Russian gas amid the ongoing war. This situation prompted discussions on energy diversification and the strategic implications for European security and economic stability.

#### 2023.

2023 saw significant **military and geopolitical developments**. Russia’s actions, such as declaring Melitopol the capital of the occupied Zaporizhzhia region and deploying tactical nuclear weapons to Belarus, were described as efforts to consolidate control and escalate tensions. The expansion of NATO, with Finland and Sweden’s formal membership (in April 2023 and March 2024, respectively), underscored shifting alliances and heightened security concerns across Europe. Coverage of these developments varied across sources, with some emphasising the security threats arising from Russia’s aggression, while others presented them as defensive measures.

Coverage consistently focused on **energy and economic security**, particularly Europe’s dependency on Russian energy. Reporting linked Europe’s energy diversification efforts and sanctions against Russia to broader geopolitical dynamics, presenting these measures in contrasting ways: some accounts emphasised their strategic importance, while others questioned their effectiveness. The destruction of the Kakhovka Dam in June was widely described as having significant environmental and humanitarian consequences.

**Disinformation** continued to play a central role, with Russian information manipulation and interference efforts aiming to influence global narratives about the war. Platforms such as Twitter and YouTube were scrutinised for their roles in spreading misleading information. Media coverage emphasised the need for stronger countermeasures, highlighting initiatives like Reporters Without Borders’ “Freedom” news package aimed at providing independent news and information to Russian-speaking audiences. Russia, in turn, sought to undermine Western solidarity with Ukraine and portray the war as a regional issue.

Other relevant narratives concerned **Ukraine’s governance challenges** and **discussions about Ukraine’s potential EU membership**.

#### 2024.

**Military developments** remained a focal point. Ukraine’s use of Western-supplied weapons, including F-16 fighter jets and long-range missiles, shaped the dynamics of the war. These military advancements were described as essential for Ukraine’s defence, while Russia condemned them as provocations. The deployment of North Korean forces and the provision of equipment from Iran illustrated how the war broadened.

**Geopolitical shifts** were reflected in NATO’s expansion and the EU’s accession talks with Ukraine and Moldova. The NATO summit and subsequent discussions on Ukraine’s potential membership underscored the West’s commitment to supporting Ukraine against Russian aggression. Meanwhile, Russia’s strategic alliances with countries such as North Korea and Iran were viewed with increasing alarm, exacerbating regional tensions and complicating diplomatic efforts.

The **diplomatic and economic dimensions** of Russia’s full-scale invasion of Ukraine also featured prominently. Efforts to mediate peace, such as Hungary’s Viktor Orbán’s attempts to broker dialogue, were met with scepticism. Sanctions against Russia intensified, with Western nations targeting military supply chains and political figures. Coverage presented these measures in contrasting ways, with some accounts emphasising their importance, while others questioned their effectiveness. Energy tensions, particularly around the Russo-Ukrainian gas transit agreement, highlighted the broader economic implications of the war.

Finally, **disinformation** continued to be a critical narrative, with Russian information manipulation and interference efforts aiming to shape global perceptions of the war. Platforms such as Twitter and YouTube faced scrutiny for spreading misleading information. Media coverage emphasised the need for stronger countermeasures, while Russia sought to undermine Western solidarity with Ukraine.

Taken together, the year-by-year analysis shows that, although the specific focus of coverage changes from year to year, media narratives remain structured around a set of recurring patterns observable across the analysed period. Major geopolitical developments, such as Russia’s annexation of Crimea in 2014 and the full-scale invasion of Ukraine in 2022, are associated with shifts in the prominence of different narratives, while recurring patterns such as geopolitical tensions, energy politics, sanctions, and disinformation remain consistently present. This illustrates how media narratives evolve through the interaction between short-term developments and longer-term patterns in how issues are described and contextualised over time. These findings complement the temporal analysis of coverage volume by showing how not only the intensity but also the content, of media attention changes over time.

### Tracking cluster chains of articles over time

This component addresses Research Question 1 by examining how narratives evolve through the linking of clusters interpreted as stories. Based on the main narratives outlined in the annual summaries, we identified the most prominent clusters for each year and tracked their evolution using cluster linking. This approach allows us to trace how related stories persist, merge, or fragment, thereby capturing narrative dynamics as patterns emerging across related stories. It complements the year-by-year overview by showing not only which narratives were prominent, but also how they developed through interactions between related stories.

Across the analysed periods, three main dynamics emerge: (i) the persistence of certain stories over extended periods, (ii) the rapid emergence and dominance of event-driven clusters during major geopolitical developments, and (iii) the branching and recombination of clusters into multiple related stories that reflect different aspects of the same event. These dynamics are illustrated through two key periods: Russia’s annexation of Crimea (2013–2014) and Russia’s full-scale invasion of Ukraine (2021–2022).

We selected two periods corresponding to key geopolitical events and extended the analysis to include the year preceding each event, namely 2013–2014 for the annexation of Crimea and 2021–2022 for the full-scale invasion of Ukraine. This enabled us to capture both the lead-up to and the unfolding of these events in media coverage. Full-period visualisations are available through the JRC Data Catalogue [22].

**Fig 3:**
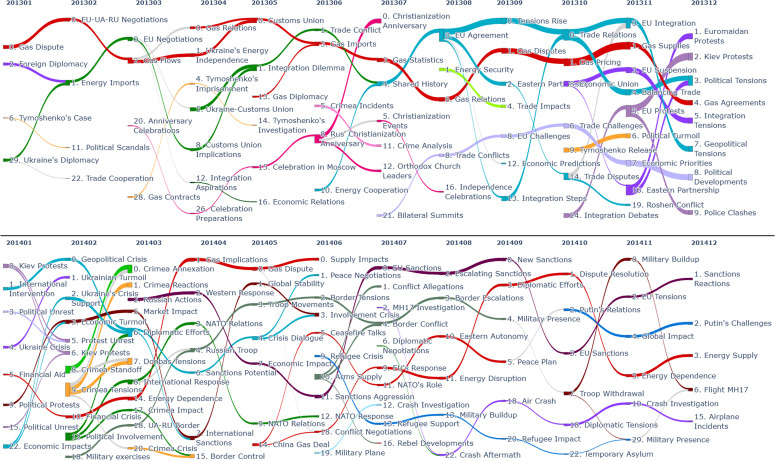
Evolution of the most prominent clusters characterising media coverage in 2013 and 2014. Each coloured band represents a distinct cluster chain, i.e., a sequence of clusters that are sufficiently similar to be linked over time. Colours are assigned automatically from a 30-colour Glasbey palette and repeat cyclically after 30 iterations. Band thickness depends on the number of articles in a cluster and the similarity between connected clusters. Visual scaling is adjusted automatically to fit the figure within the page layout for each year. The interactive HTML version of the figure is available in the dataset repository [22].

For the period 2013–2014, as shown in Fig 3, the analysis focused on five key clusters, as follows.

First, clusters related to the **Russia–Ukraine gas dispute** appear throughout 2013 and 2014. The first cluster of articles, represented by the red line starting as cluster zero in the 2013 visualisation of Fig 3, spans from January 2013 to June 2014, initially with around 600 articles per month. A significant increase occurred in September 2013, with the number of monthly articles exceeding 1,300. It continued to grow, reaching nearly 2,800 by December 2013 and surpassing 5,510 articles by June 2014. This surge indicates heightened media interest and public concern during this period.

As the cluster evolved, it increasingly linked reporting on the gas dispute and its implications, including Ukraine’s efforts to diversify its energy sources and, later, reporting on contractual agreements involving reduced gas prices that maintained Ukraine’s reliance on Russia. A second cluster, also represented by a red line starting as cluster 14 in May 2014, captured the global implications of the gas dispute, particularly its impact on the EU. The increasing thickness of the red line over time signifies the growing prominence of this cluster, with monthly article numbers rising from 2,000 in May to more than 5,000 by December.

Second, clusters related to the **Euromaidan protests** appeared from October 2013 to February 2014. Although the protests began on 21 November 2013, four clusters related to the demonstrations dominated coverage in December (see clusters one, two, five, and nine in December 2013 in Fig 3). Tracing the clusters back to October shows that coverage already framed the protests in relation to Ukraine’s pivotal choice between strengthening ties with the EU through the signing of the Association Agreement and aligning with Russia by joining the Eurasian Economic Union (EEU).

As these clusters evolved into 2014, they became more prominent, with five out of the eight main clusters in January reporting on the anti-government demonstrations and the ensuing political crisis. However, coverage of the protests diminished in February as media attention shifted towards developments in Crimea.

Third, clusters related to **Crimea** dominated media coverage in February and March 2014. In February, three of the main clusters (see clusters eight, nine, and 12), each containing over 8,000 articles, focused on the “Crimea crisis,” reporting on the pro-Russian demonstrations in a region with a large ethnic Russian population, and highlighting Russia’s involvement and the ensuing international concern.

In March, nine out of the 11 main clusters identified (all except cluster 14, which pertained to energy, and cluster 18, which focused on border tensions between Russia and Ukraine) centred on Russia’s annexation of Crimea, amounting to over 100,000 articles. While all these clusters pertained to the annexation, each approached the issue from a distinct angle: cluster zero detailed the events, cluster one examined the international reactions and implications, cluster five explored the impact on global markets and economies, and others highlighted additional dimensions of the crisis.

Fourth, clusters related to **sanctions against Russia** became a defining feature of news coverage from March 2014 onward. The purple line, starting as cluster two in March and comprising over 19,000 articles, can be traced through to the end of the year. After emerging as a dominant pattern in coverage in the immediate aftermath of the annexation, the cluster temporarily declined in May and June, before re-emerging as the leading cluster in July, August, and September. Following a brief decline, it again became the most prominent cluster in December.

As detailed in the monthly summaries (see supporting information), media coverage during this period was largely dominated by the EU’s expansion of sanctions against Russian officials and entities, aimed at economically and politically isolating Russia. Although this analysis focuses only on the period immediately following the annexation of Crimea, it is evident from the annual summaries that sanctions remained a recurring feature of media coverage throughout the analysed period.

Fifth, clusters related to the **Malaysia Airlines Flight MH17** appeared after the aircraft was downed in July 2014, and continued throughout the remainder of the year. In July, three major clusters centred on the plane crash. Interestingly, one of these clusters was linked to an earlier cluster from June concerning a Ukrainian military aircraft shot down by pro-Russian separatists. The other two clusters subsequently merged into a single cluster in August, focusing on the air crash investigation.

In conclusion, the analysis of cluster evolution from 2013 to 2014 illustrates how media coverage is structured through the interaction of persistent and event-driven stories. While clusters related to the Russia–Ukraine gas dispute persisted throughout the period, others emerged rapidly and came to dominate coverage at specific moments, such as the Euromaidan protests, the annexation of Crimea, sanctions against Russia, and the MH17 plane crash. The branching and merging of these clusters further demonstrate how different aspects of the same developments were reported across multiple related stories. Taken together, these dynamics illustrate how narratives evolve through the interaction and transformation of related stories over time.

**Fig 4:**
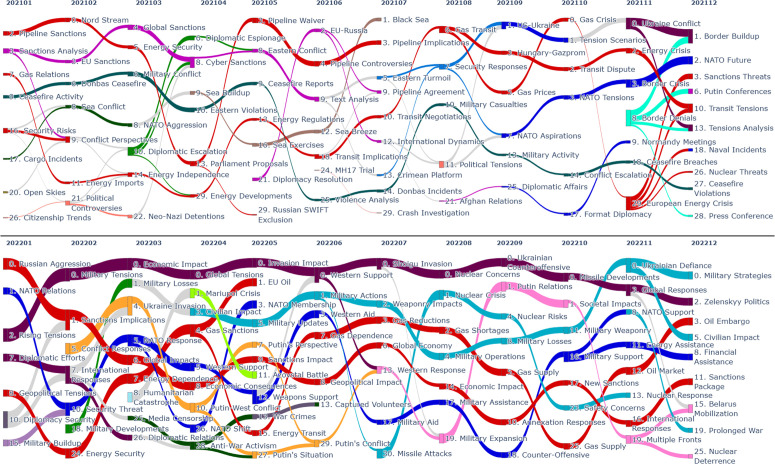
Evolution of the most prominent clusters characterising media coverage in 2021 and 2022. Each coloured band represents a distinct cluster chain, i.e., a sequence of clusters that are sufficiently similar to be linked over time. Colours are assigned automatically from a 30-colour Glasbey palette and repeat cyclically after 30 iterations. Band thickness depends on the number of articles in a cluster and the similarity between connected clusters. Visual scaling is adjusted automatically to fit the figure within the page layout for each year. The interactive HTML version of the figure is available in the dataset repository [22].

In the second key period, 2021–2022, coverage increasingly centred on **Russia’s full-scale invasion of Ukraine**. As shown in Fig 4, in February 2022, all but one of the major clusters (cluster 24, related to the gas crisis) focused on the invasion, together accounting for approximately 157,000 articles.

Further examination of specific clusters related to the invasion revealed distinct patterns in how events were described and linked over time, allowing us to trace the evolution of narratives. Cluster zero in February, documenting the unfolding invasion and comprising over 60,000 articles, was linked to clusters zero and eight in November 2021. Cluster eight in November captured reporting on the Kremlin’s denial of invasion preparations.

This November cluster, in turn, branched into four clusters in December: cluster one, addressing rising border tensions and international responses; cluster 13, examining the implications of these developments; cluster 28, covering Vladimir Putin’s annual press conference, where he expressed concerns over Ukraine’s NATO ties and criticised Western support for Ukraine; and cluster six, containing media analyses and commentary on the press conference captured in cluster 28.

Tracing the evolution of these clusters over time allowed us to identify the main patterns in coverage preceding the invasion: Putin’s denial of any invasion intentions, coupled with accusations that Ukraine was aligning too closely with NATO, and the international community’s apprehension over escalating tensions.

In conclusion, the cluster-linking analysis for 2021–2022 shows how narratives surrounding the full-scale invasion emerged from pre-existing stories and rapidly consolidated into dominant patterns of media coverage. The evolution of clusters highlights how early reporting on rising tensions, troop build-ups, and diplomatic exchanges gave way to a dominant focus on the invasion itself, while remaining linked to earlier reporting on NATO expansion, Russia’s security concerns, and international responses.

Overall, the analysis of these two periods highlights how narrative dynamics operate across different phases of the conflict. In both cases, periods of escalating tensions were characterised by the coexistence of multiple related stories, which were subsequently consolidated into a smaller number of dominant patterns of coverage as events intensified. At the same time, earlier stories did not disappear entirely but continued to shape the contextualisation of later developments. This comparison illustrates how media narratives evolve through the interplay between continuity and change, with new developments both reshaping and building on pre-existing patterns of coverage.

### Summary of key findings on the evolution of media narratives

Taken together, the first three components of the analysis provide a comprehensive answer to Research Question 1. Media coverage of Russia and Ukraine between 2013 and 2024 is characterised by strong event-driven dynamics, with major geopolitical developments triggering sharp increases in attention. At the same time, the analysis of clustered and annotated data shows that coverage is structured around a set of recurring narrative patterns, including geopolitical tensions, energy politics, sanctions, and disinformation, which persist across the analysed period, as reflected in the dominant narratives identified in the annual summaries and their cross-year synthesis.

The cluster-linking analysis further demonstrates that these narratives evolve over time through the emergence, interaction, and transformation of related stories, with periods of escalation leading to the consolidation of dominant patterns of coverage. Overall, these findings indicate that media narratives evolve through the interplay between short-term developments and longer-term patterns in how issues are described and contextualised over time.

### Main entities

**Fig 5:**
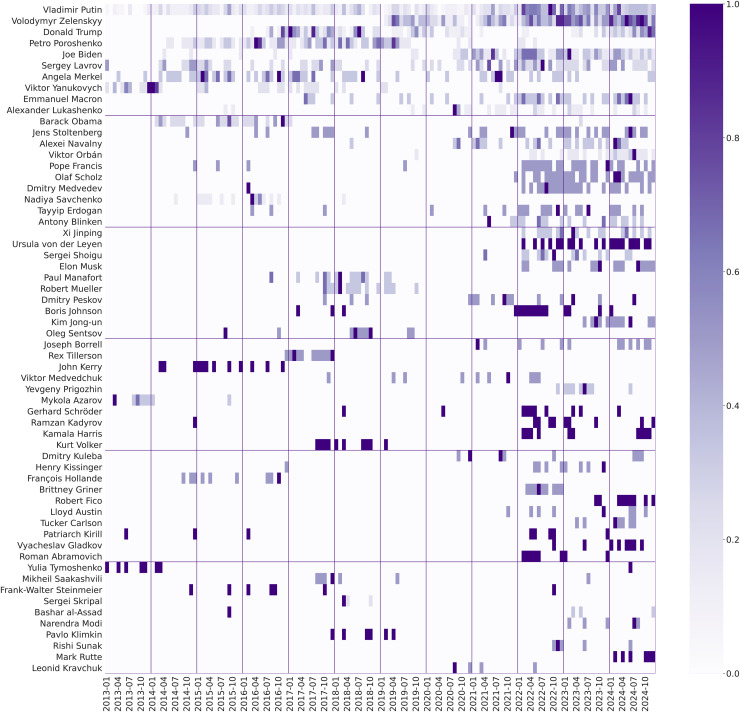
Most frequently mentioned entities across the top clusters by month. The heat map shows the most frequently mentioned entities across the top clusters of each month. Entities are ordered according to their total number of mentions over the analysed period.

The analysis of the most frequently mentioned entities across each month’s top clusters provides important contextual insights, showing how media attention repeatedly coalesced around a relatively small set of political leaders who became emblematic of different phases of the conflict. These observations are based on the association of named entities with the monthly summaries derived from the annotated data. As shown in Fig 5, the top ten most frequently mentioned entities were:

1. Vladimir Putin, President of Russia throughout the analysed period;
2. Volodymyr Zelenskyy, President of Ukraine since 2019;
3. Donald Trump, President of the United States from 2017 to 2021 (and from 2025);
4. Petro Poroshenko, President of Ukraine from 2014 to 2019;
5. Joe Biden, Vice President of the United States from the start of the analysed period to 2017 and President from 2021 to the end of the monitored period;
6. Sergey Lavrov, Foreign Minister of Russia throughout the analysed period;
7. Angela Merkel, Chancellor of Germany from the beginning of the monitored period to 2021;
8. Viktor Yanukovych, President of Ukraine from the start of the analysed time frame to 2014;
9. Emmanuel Macron, President of France since 2017; and
10. Alexander Lukashenko, President of Belarus throughout the analysed period.

All of these are political figures who held public office either throughout or at some point during the monitored period. Vladimir Putin is consistently among the most frequently mentioned figures, with a marked peak in 2022 corresponding to Russia’s full-scale invasion of Ukraine. After the annexation of Crimea in 2014, mentions of Putin were frequently associated with clusters concerning military escalation, sanctions, international condemnation, and reported violations of international norms, while less prominent clusters portrayed him as a defender of national interests. Following the full-scale invasion of Ukraine in 2022, reporting consistently characterised him as a belligerent aggressor responsible for an unprovoked war, alongside less prevalent clusters linking his actions to the protection of Russia from perceived external threats.

Volodymyr Zelenskyy emerged in 2019 upon becoming President of Ukraine; as shown in Fig 5*,* he was mentioned more often than his Russian counterpart in 2023 and 2024 across the monitored dataset. Before the full-scale invasion, coverage frequently described Zelenskyy as pursuing a conciliatory approach towards Russia, with articles highlighting criticism of his handling of tensions. Following the full-scale invasion, this pattern shifted markedly, with reporting increasingly associating Zelenskyy with leadership, resilience, and efforts to mobilise international support for Ukraine.

Overall, Donald Trump was mentioned more frequently than Joe Biden. Mentions of Trump rose in 2016, coinciding with his first presidential campaign, and remained high throughout his first term in office. They increased again in 2023 and during his second presidential campaign in 2024. These data indicate that the Russo-Ukrainian conflict was a significant topic in US discourse during both of Trump’s presidential campaigns.

Before and during Trump’s first presidency, the articles analysed often reflected concerns about potential shifts in US foreign policy, with coverage associating Trump with a conciliatory stance towards Moscow. His interactions with Putin and mixed messages on sanctions and US–Russia relations were linked to debates about his commitment to addressing Russian aggression.

Speculation persisted ahead of his potential second presidency, with coverage frequently raising questions about whether his leadership would weaken or halt US support for Ukraine and favour Russian interests.

Mentions of Petro Poroshenko aligned with his presidential mandate. Following his election in 2014, coverage frequently described him as seeking to end the conflict in eastern Ukraine, restore stability, and strengthen ties with Western partners.

Joe Biden was mentioned less frequently than Donald Trump, even though he served as Vice President and then President for nine out of the 12 years covered by the analysis. His mentions were sporadic during his vice presidency, nearly absent in the two years that followed, and resurged during the 2020–2021 presidential campaign before intensifying during his presidency. This pattern confirms that Russia’s war against Ukraine was a significant topic in US discourse during the 2020–2021 campaign.

During his vice presidency, coverage frequently associated Biden with support for sanctions and stronger measures against Russia. During his presidency, reporting increasingly described him as resolute, warning of severe economic consequences if Russia escalated military actions in Ukraine. His diplomatic efforts were presented as attempts to prevent further escalation, focusing on security assurances for Ukraine, though coverage also highlighted the challenges and limited progress in de-escalating tensions.

Mentions of Sergey Lavrov were persistent throughout the analysed period, with peaks during and after Russia’s annexation of Crimea in 2014 and the full-scale invasion of Ukraine in 2022. Coverage consistently described Lavrov as a key defender of the Kremlin’s foreign policies, with reporting associating him with narratives describing Crimea’s annexation in terms of self-determination, as well as with critiques of NATO and Western actors.

Mentions of Angela Merkel remained relatively constant throughout her time in office and during 2022, before declining as attention shifted to Germany’s new leadership. Mentions of Olaf Scholz emerged as he took office and remained sustained through to the end of the analysed period. Coverage consistently described Merkel as a central figure in European diplomacy and a steadfast advocate for maintaining a firm stance on Russia, especially in relation to the Ukraine crisis. Reporting also associated her with efforts to reinforce the EU’s commitment to Ukraine’s territorial integrity and to implement sanctions against Russia. Her interactions with leaders such as Vladimir Putin were frequently covered, underscoring her role in attempts to address geopolitical tensions and mediate peace.

Mentions of Viktor Yanukovych were mainly concentrated at the end of 2013 and the beginning of 2014, when his decision to suspend the EU Association Agreement sparked the Euromaidan protests. Coverage frequently described Yanukovych as closely aligned with Russian interests, with reporting highlighting allegations of corruption and political repression. His ousting in 2014 amidst violent clashes was widely described as a rejection of his pro-Russian stance and style of governance. Subsequent legal challenges, including treason charges, further underscored his controversial legacy, as Ukraine sought to distance itself from Russian influence and pursue democratic reforms.

Mentions of Emmanuel Macron coincided with the beginning of his term and intensified from 2022, reflecting his efforts to position France as a key player in the international response to the crisis. Coverage frequently associated him with diplomatic initiatives and international mediation efforts.

Mentions of Alexander Lukashenko were sporadic but intensified after the full-scale invasion, with coverage increasingly associating him with closer alignment with Moscow. This alignment was particularly evident after Belarus was sanctioned alongside Russia following the invasion.

Finally, the analysis of the most frequently mentioned entities reveals two additional noteworthy findings. First, Elon Musk, the wealthiest person in the world and a senior adviser to US President Donald Trump during the early months of his second presidency in 2025, ranks as the 24th most mentioned entity overall. Mentions of Musk increased in parallel with his growing involvement in discussions related to Russia’s war against Ukraine and, more broadly, in international politics.

Second, Patriarch Kirill, head of the Russian Orthodox Church throughout the analysed period, ranks as the 48th most mentioned entity overall. His mentions were sporadic but showed distinct peaks coinciding with key events, such as Ukraine’s push for autocephaly and broader religious tensions reflecting Ukraine’s struggle for independence and identity. Kirill’s involvement underscores the intersection of religion and politics in the Russo-Ukrainian conflict.

### Summary of key findings on the evolution of entities (RQ2)

Overall, the analysis of the most frequently mentioned entities shows that media attention consistently centred on a relatively small group of political leaders, whose prominence fluctuated in line with major developments in the conflict. Rather than appearing in isolation, these individuals were repeatedly linked to specific events and developments through clustered media coverage, indicating that their salience is closely tied to how key issues are reported and contextualised over time. This highlights the extent to which individual actors function as focal points within broader patterns of coverage.

### Language distribution of articles by Russian sources

This component of the analysis addresses Research Question 3 by examining the language distribution of articles published by Russian sources and how it evolves over time. In addition to identifying the main languages used, the analysis explores how shifts in language distribution relate to patterns in the content of coverage, providing insights into how reporting is structured across language groups.

**Fig 6:**
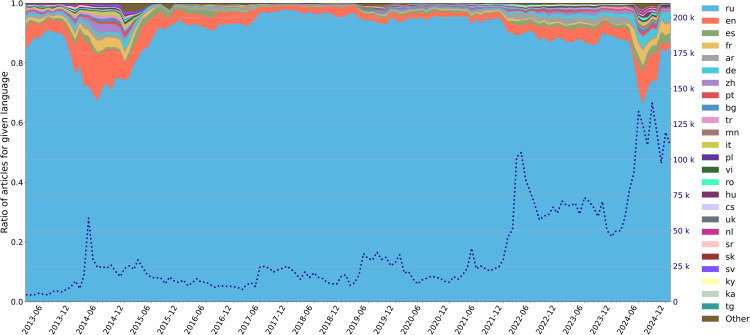
Language distribution of articles published by Russian sources. The dark blue dotted line represents the monthly total number of articles published by Russian sources.

As illustrated in Fig 6, the dominant language used by Russian sources is Russian, representing over 86.5 per cent of articles. Following Russian, the most frequently used languages by these outlets were English (4.6 per cent), Spanish (1.4 per cent), French (1.1 per cent), Arabic (1.1 per cent), and German (1.1 per cent). The substantial number of articles in these foreign languages indicates an extension of coverage beyond Russian-speaking contexts.

Shifts in the relative share of foreign-language publications were particularly visible at two key moments. The first coincided with Russia’s annexation of Crimea in early 2014, with the proportion of foreign-language articles rising at the end of 2013 and reaching a peak in the months following the annexation. The second aligned with Russia’s full-scale invasion of Ukraine. The increase in the share in foreign-language publications began at the end of 2021, continued throughout 2022 and most of 2023, and culminated in a peak in mid-2024, associating the trends with major geopolitical developments.

**Fig 7:**
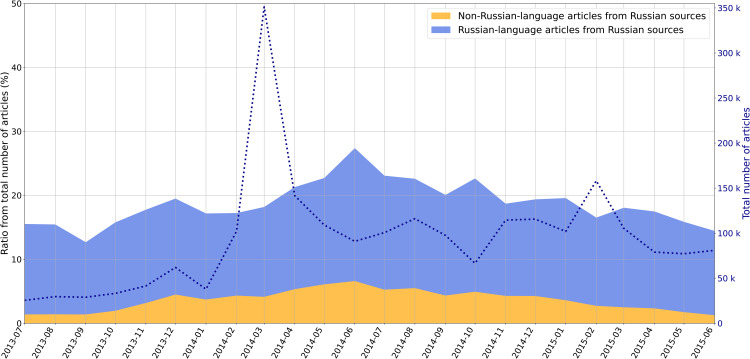
Evolution of Russian- and non-Russian-language articles published by Russian sources during the period surrounding the annexation of Crimea (mid-2013 to mid-2015). The stacked areas represent the share of these articles relative to the total number of articles, while the dotted line indicates the total number of articles collected during the period.

**Fig 8:**
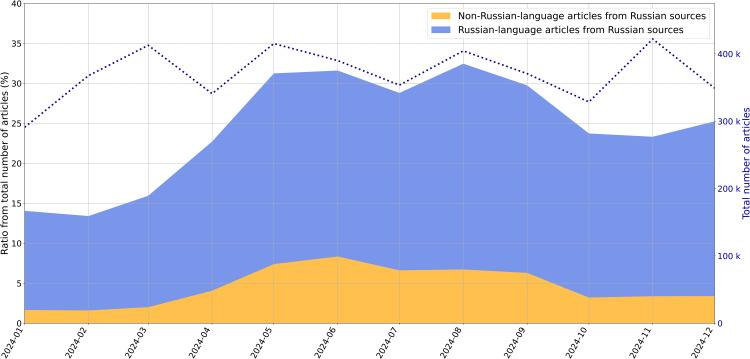
Evolution of Russian- and non-Russian-language articles published by Russian sources in 2024. The stacked areas represent the share of each language group relative to the total number of articles, while the dotted line indicates the total number of articles collected during the period.

To further examine these patterns, we conducted a focused analysis of these two periods, combining quantitative and qualitative perspectives. In addition to tracking changes in the ratio of Russian sources’ articles across languages (Fig 6), we examined the content of articles in both Russian and non-Russian languages during peak months using cluster-based methods. Figs 7 and 8 illustrate the time evolution of overall proportion of the two language groups for periods from mid-2013 to mid-2015 and the full year 2024, respectively. For each period, we selected the month with the highest proportion of non-Russian-language articles (June 2014 and June 2024) and analysed the two language groups separately to identify differences in how coverage was structured across linguistic contexts.

In June 2014, Russian-language coverage was structured around the ongoing conflict, diplomatic efforts, humanitarian developments such as refugee flows into Russian border regions, and energy-related issues, particularly gas disputes between Russia and Ukraine. In contrast, non-Russian-language coverage, while also addressing the conflict and diplomatic processes, placed more consistent emphasis on geopolitical tensions and international responses. In particular, it focused on relations between Russia, Ukraine, and Western actors, including NATO, the United States, and the European Union, often in connection with sanctions and broader security dynamics. Humanitarian and economic themes were present but less central within this language group.

This comparison indicates that, in June 2014, the foreign-language group emphasised geopolitical and international dimensions of the conflict, whereas the Russian-language group encompassed a broader range of issues, including humanitarian and economic aspects.

Unlike the 2014 peak, which followed Russia’s annexation of Crimea, the 2024 increase occurred during a period of stable overall monthly reporting on the war and was not associated with a single dominant event. This trend suggests that the rise in foreign-language publications reflects a shift within an already elevated level of attention, rather than a discrete, event-driven surge.

For June 2024, the Russian-language group emphasised military developments on the ground. The most prominent clusters focused on advances in eastern Ukraine, ongoing battles in regions such as Kharkiv and Donetsk, and the use of drones, air defence systems, and missile strikes. Substantial attention was also devoted to cross-border attacks and Ukrainian strikes on Russian territory, including in regions such as Belgorod and Sevastopol.

Alongside these developments, coverage incorporated an international dimension, with references to Western military support, NATO involvement, and diplomatic processes. Taken together, Russian-language coverage was centred on military operations, while also emphasising Western involvement and diplomatic initiatives. At the same time, Russian-language sources highlighted a “peace proposal” (terminology used by Russian sources) put forward by President Putin.

In contrast, the non-Russian-language group focused on Ukraine’s offensive into Russian territory, the Ukraine Peace Summit held in Switzerland on 15 and 16 June (preceding President Putin’s “peace proposal”) and NATO’s deepening involvement in the war, including US announcements of substantial new military aid packages featuring advanced weaponry.

The foreign-language group prominently featured narratives portraying Ukraine as escalating the conflict by attacking Russian territory, and Western actors, particularly NATO and the United States, as complicit through their continued provision of arms. Conversely, Russia was often presented as pursuing de-escalation and peace through diplomacy.

### Summary of key findings on the language distribution of articles by Russian sources (RQ3)

Overall, the analysis shows that the language distribution of articles published by Russian sources is highly concentrated in Russian, but includes a consistent share of foreign-language output available to a range of international audiences. Shifts in the proportion of foreign-language articles are not uniform over time, but become particularly visible during periods of heightened geopolitical tensions.

The focused analyses of these periods indicate that increases in foreign-language output are associated with changes not only in volume, but also in the structure of coverage. While Russian-language reporting incorporates a broader range of themes, foreign-language coverage places greater emphasis on international responses, geopolitical dynamics, and diplomatic initiatives. Differences in how military developments are described across language groups further indicate that shifts in language distribution are associated with variations in how the conflict is contextualised across audiences. These findings indicate that language distribution is not only a descriptive feature of media output, but also relates to differences in how key developments are prioritised and presented across linguistic contexts.

## Discussion and conclusion

This study identifies a set of recurring media narratives concerning Russia and Ukraine that persist over time while shifting in prominence in response to major geopolitical events. Drawing on the analysis of 22.2 million article titles from 14,622 sources across 194 countries between 2013 and 2024, the findings provide a large-scale, longitudinal perspective on how these narratives are structured and evolve. This section discusses these findings in relation to the research questions.

Regarding the dominant media narratives identified in the study (main research question), our analysis highlights recurring patterns in coverage, with significant fluctuations during key geopolitical events such as the Euromaidan protests, the annexation of Crimea, the Minsk Agreements, the military build-up at the Russia–Ukraine border, and the full-scale invasion of Ukraine. These findings corroborate previous research on narratives related to Russia–Ukraine relations since 2013, particularly those focusing on the Euromaidan events and Crimea [33–38].

With respect to the evolution of media coverage over time (RQ1), we used clustering techniques to produce monthly and yearly summaries of media narratives, identifying persistent patterns in how key issues were described and contextualised over time. These recurring narrative patterns include geopolitical tensions, energy politics, and disinformation campaigns.

Coverage consistently highlighted the ideological clash between Western democratic values and Russian authoritarianism, positioning Ukraine as a central battleground. Energy-related reporting frequently emphasised Ukraine’s dependence on Russian gas and the broader strategic implications for European security, particularly in relation to projects such as Nord Stream 2. Disinformation-related coverage linked Russian actions to efforts to shape global perceptions, legitimise its positions, and undermine Western support for Ukraine.

These findings are consistent with previous research on narratives depicting Russia as threatened by NATO [35,37,39–44], as well as studies on the role of disinformation narratives in shaping media discourse [12,45–47].

By employing cluster-linking techniques, we successfully tracked the evolution of article clusters, revealing how these narratives shifted in response to major events. For the 2013–2014 period, narratives related to the Russia–Ukraine gas dispute, the Euromaidan protests, and the annexation of Crimea were prominent. For the 2021–2022 period, Russia’s full-scale invasion of Ukraine and its global repercussions dominated media coverage.

Manual inspection by analysts of the clusters underlying the monthly and yearly summaries corroborated the results of the automated cluster-linking procedure. This convergence demonstrates the robustness and value of our approach.

Regarding the role of key actors in media coverage (RQ2), the analysis of the most frequently mentioned entities in media narratives identified Vladimir Putin, Volodymyr Zelenskyy, and Donald Trump as central figures in the discourse on Russia’s aggression against Ukraine. Mentions of Putin were frequently associated with clusters concerning military escalation, sanctions, international condemnation, and reported violations of international law.

Mentions of Zelenskyy increased significantly after 2019, with coverage increasingly associating him with Ukrainian resistance following the full-scale invasion. Trump’s prominence during his campaigns indicates that Russia’s war against Ukraine was a significant topic in US discourse, with coverage frequently questioning whether he would decrease or halt US support for Ukraine.

Other notable figures included Joe Biden, Sergey Lavrov, Petro Poroshenko, and Angela Merkel, each associated with distinct patterns in coverage at different times.

Concerning the language distribution of Russia-linked media (RQ3), the multilingual output of Russian sources was examined to assess how content is distributed across audiences. While the vast majority of articles were in Russian, content was also published in English, Spanish, French, Arabic, and German, extending coverage beyond Russian-speaking contexts. This finding is consistent with previous research documenting the presence of Russian narratives across multiple languages and regions [42–45,48–53].

Notably, the share of foreign-language articles increased during key geopolitical moments, including the annexation of Crimea in 2014 and the period following the full-scale invasion of Ukraine. Our analysis further shows that these shifts are associated with differences in how the conflict is structured and contextualised across language groups, with foreign-language coverage placing relatively greater emphasis on geopolitical dynamics and international responses.

Taken together, these findings show how recurring narrative patterns, their evolution over time, the prominence of key actors, and variations across language contexts jointly structure media coverage.

From a methodological perspective, this study distinguishes itself through its extensive dataset of over 22 million articles from 14,622 sources across 194 countries covering the period from 2013 to 2024, which is, to our knowledge, unparalleled in the scientific literature.

Our approach detects clusters of semantically similar articles, traces their evolution over time, and identifies recurring patterns in how issues are represented in media coverage. The scale and complexity of the dataset required a hybrid analytical pipeline that combined smaller, efficient models for pre-processing and clustering with larger models for post-processing tasks such as keyword extraction, summarisation, and narrative interpretation, where deeper understanding was required.

This pipeline ensured computational efficiency while preserving analytical depth and accuracy. We integrated checkpoints that allowed human analysts to intervene, adjust parameters, and optimise model performance. This synergy between machine processing and human insight proved invaluable, as automated outputs enabled analysts to identify key features in the dataset, while analyst-generated summaries corroborated the cluster-linking results.

Beyond its methodological contribution, the relevance of our findings lies in their connection to broader theoretical discussions on how media narratives about Russia and Ukraine reflect competing interpretations of geopolitical events and contribute to shaping public discourse in international affairs [7,9,11].

Recurring patterns in coverage, such as references to “NATO as a threat to Russia” or “Ukraine as the aggressor”, illustrate how competing interpretations of events coexist and circulate in the global media space. Similarly, recurring references to the illegality of the annexation of Crimea or to Russia’s full-scale invasion of Ukraine point to more stable patterns in how events are described.

Taken together, these patterns highlight the coexistence of competing and reinforcing interpretations in a rapidly evolving information environment.

### Limitations

The findings of this study should be interpreted in light of several methodological limitations. First, the results are inherently shaped by the selection and evolution of sources included in the dataset. The number of monitored sources increased substantially over time, from 1,220 in January 2013 to 10,417 in December 2024, influencing the volume of collected articles across the analysed period. In addition, EMM source country-of-origin labels do not always correspond to headquarters, ownership, or legal registration of the source domain. When a source has multiple sub-domains operating across different countries, the country of origin for each of them is assigned based on the primary operational context.

In addition, source coverage is geographically uneven, with a concentration in Europe, the United States, and Russia, and more limited representation from Latin America, Africa, and Asia. As a result, the identified narrative patterns may reflect the perspectives and reporting priorities of more heavily represented regions, which may limit the generalisability of the findings and the ability to draw robust cross-regional comparisons.

Second, while four analysts were involved in the annotation process, each monthly output was reviewed by a single analyst. Due to time and resource constraints, we did not conduct systematic double annotation or formal inter-annotator agreement testing. Although the annotation procedure was jointly developed, refined through a calibration phase, and supported by regular discussions, some degree of subjectivity in the interpretation of clusters and the identification of narratives cannot be excluded.

This may have influenced the interpretation of clusters into stories and the identification of narratives, particularly in cases where multiple plausible interpretations of the underlying patterns were possible.

Third, the clustering is dependent on the choice of the resolution parameter, which affects the granularity of resulting clusters. The value of the parameter was defined after manual inspection of a sample month. Subsequently, the process relied on AI-based algorithms applied to article titles rather than full texts, due to legal constraints [54,55]. While this approach enabled the analysis of a large multilingual dataset, it may have limited the depth and nuance of the identified patterns, as titles do not fully capture the complexity of the underlying articles.

In addition, the outputs are shaped by the assumptions and limitations of the underlying models, which may have influenced how certain narrative elements were captured. Consequently, some narrative patterns may be simplified or underrepresented, particularly those requiring deeper contextual interpretation.

Finally, the analysis is based on observed patterns in media content and does not allow for direct inference about the intentions underlying the production and dissemination of this content. Accordingly, findings related to language use should be interpreted as descriptive of observed content patterns, rather than as evidence of deliberate communication strategies.

### Future research

Recognising these limitations, future work will focus on expanding the geographical coverage of sources monitored by Europe Media Monitor (EMM). Although the number of monitored outlets grew substantially over the 12-year period, reaching more than 25,000 by the end of the timeframe, our analysis revealed persistent gaps, particularly in Eastern Europe and the Global South.

Addressing these imbalances would make it possible to compare narrative prevalence across regions and languages, thereby offering a more nuanced understanding of how information dynamics unfold across different audiences and media ecosystems.

To further address limitations related to language and source distribution, a promising line of research is to examine the role of non-Russian outlets publishing in Russian, including the extent to which such media reach Russian-speaking audiences within and outside Russia. Preliminary evidence suggests that the share of Russian-language publications by non-Russian sources has declined markedly since 2021, coinciding with Russia’s full-scale invasion of Ukraine and the tightening of its information environment.

A systematic analysis of these trends could clarify whether and how Russian-language content produced outside Russia contributes to the circulation of alternative narratives or provides counterpoints to Kremlin-aligned messaging.

Future work could also address limitations related to the interpretation of narrative patterns by incorporating more systematic validation procedures, such as double annotation or inter-annotator agreement measures, to assess the robustness of the annotation process and, in turn, the identification of narratives.

In addition, expanding the analysis to full-text data, where legally feasible, could provide a richer and more nuanced understanding of how narratives are constructed and conveyed.

Building on the entity analysis presented here, future studies could also compare the prominence of specific actors across linguistic or regional media. Another avenue is to link narrative shifts to external variables, such as public opinion data, policy debates, or electoral outcomes, to better assess the societal significance of these patterns.

Finally, future work could test the transferability of this methodology to other domains such as climate change, migration, and public health, enabling the analysis of datasets that are too large for manual inspection and facilitating further automation, particularly in time-critical contexts.

Taken together, these directions highlight the broader importance of continuing research on large-scale media narratives using hybrid human-AI approaches. As information environments become increasingly complex and fast-moving, methods capable of systematically identifying patterns across vast datasets are essential not only for understanding how public discourse evolves over time, but also for supporting timely situational awareness in rapidly changing information environments.

Expanding this line of work through improved data coverage, more robust validation procedures, and richer analytical inputs, such as full-text data and applications of this approach to other domains, would enhance both the depth and reliability of insights. At the same time, integrating these approaches with external data sources, including public opinion and policy developments, offers the potential to better assess the societal relevance of these patterns.

Together, these advances would contribute to a more comprehensive and nuanced understanding of how narratives emerge, interact, and shape perceptions in contemporary information ecosystems.

## Supporting information

S1 Table Yearly breakdown of dataset characteristics. The table provides a yearly breakdown of dataset characteristics, including the number of articles, sources, languages, and countries.
(DOCX)

S1 Text Cluster validation examples for January 2013. Examples of randomly selected clusters and sampled headlines from January 2013 demonstrating cluster coherence across sources and languages.
(DOCX)

S2 Table Major news events corresponding to the peaks in media coverage shown in Fig 2. The table associates the peaks visible in Fig 2 with the major news events to which they are related. (DOCX)

S3 Table LLM settings. The table provides details on parameter settings and prompt structures for large language models.
(DOCX)

S2 Text Monthly summaries. The monthly summaries, compiled by analysts using the clustering output and manual annotations of the top 30 clusters, present the dominant media narratives for each month of the analysed period (from January 2013 to December 2024).
(DOCX)
