# Supplementary material for: Tracking the narrative: A data-driven analysis of media coverage of Russia and Ukraine 2013–2024
Source: PLoS One. 2026 Jun 25;21(6):e0351627. doi: 10.1371/journal.pone.0351627 (PMC13298780; doi:10.1371/journal.pone.0351627)
Supplement: S1 Table — The table provides a yearly breakdown of dataset characteristics, including the number of articles, sources, languages, and countries. (DOCX) [file pone.0351627.s001.docx]

# $F_{C1}\left( x,y \right) = p_{xy}$

$$sym_{C1,C2} = \frac{1}{N_{2}}\sum_{t\in C2} F_{C1}\left( t_{x}, t_{y} \right), \left| C2 \right| = N_{2}$$

**S1 Table. Yearly breakdown of dataset characteristics.** The table provides a yearly breakdown of dataset characteristics, including the number of articles, sources, languages, and countries.

| **Year** | **Articles** | **Sources** | **Languages** | **Countries** |
| --- | --- | --- | --- | --- |
| 2013 | 329593 | 2809 | 55 | 153 |
| 2014 | 1443613 | 3613 | 64 | 172 |
| 2015 | 979120 | 3914 | 71 | 174 |
| 2016 | 657451 | 4076 | 61 | 171 |
| 2017 | 747477 | 4634 | 58 | 170 |
| 2018 | 716339 | 4783 | 61 | 172 |
| 2019 | 707298 | 4963 | 55 | 170 |
| 2020 | 449732 | 5415 | 59 | 167 |
| 2021 | 783181 | 6383 | 64 | 179 |
| 2022 | 6243753 | 9991 | 71 | 192 |
| 2023 | 4696724 | 10471 | 74 | 194 |
| 2024 | 4459402 | 10437 | 74 | 193 |
