# Supplementary material for: Tracking the narrative: A data-driven analysis of media coverage of Russia and Ukraine 2013–2024
Source: PLoS One. 2026 Jun 25;21(6):e0351627. doi: 10.1371/journal.pone.0351627 (PMC13298780; doi:10.1371/journal.pone.0351627)
Supplement: S1 Text — Examples of randomly selected clusters and sampled headlines from January 2013 demonstrating cluster coherence across sources and languages. (DOCX) [file pone.0351627.s002.docx]

# $F_{C1}\left( x,y \right) = p_{xy}$

$$sym_{C1,C2} = \frac{1}{N_{2}}\sum_{t\in C2} F_{C1}\left( t_{x}, t_{y} \right), \left| C2 \right| = N_{2}$$

**S1 Text. Cluster validation examples for January 2013.** Examples of randomly selected clusters and sampled headlines from January 2013 demonstrating cluster coherence across sources and languages.

Example 1: [Cluster title: "Russia–Ukraine Gas Dispute and Its Implications"]

Sample headlines:

“Украина за 2012г. уменьшила транзит газа в Западную Европу на 19,7%.”

“Tok ruského plynu cez Slovensko výrazne oslabol”

Hódít a norvég gáz - lehagyhatják Oroszországot”

“Gazprom pierde monopolul asupra gazelor în Europa”

“Expert ucrainean: "În următoarele zile Gazprom ar putea sista livrările de gaze”

“Czechs open new pipeline importing Russian gas”

“Moscow Kiev negotiate new formats for gas supplies to Ukraine transit – Russia’s Foreign Minister”

“Brussels not commenting on new gas row between Kyiv, Moscow”

“Financial Times: Gazprom bill puts strain on Ukraine finances”

“Ukraine buys 33 billion cubic meters of gas in 2012”

“Russia Eyes New Formats Of Cooperation With Ukraine In Gas Supplies And Transit To European Consumers”

Interpretation: this cluster groups articles addressing issues related to Russian gas and its transit to European consumers. The content is consistently characterised by recurring terms such as “gas supplies,” “Ukraine,” “Russia,” and “Gazprom.” The sampled headlines portray the issue as gas disputes and their economic and political implications for Ukraine–Russia relations. This illustrates internal cluster coherence across diverse sources and languages.

Example 2: [Cluster title: “Diplomatic Relations and Meetings between Ukraine and Russia”]

Sample headlines:

“Виктор Янукович не планира да посещава Русия преди срещата Украйна-ЕС през февруари”

“Les chefs de diplomaties ukrainienne et russe se sont trouvés une «chimie»”

“Министърът на външните работи на Русия ще посети Украйна”

“Russischer und ukrainischer Außenminister zusammengetroffen: Chemie stimmt”

“Ministr obrany ČLR provedl tajemnou návštěvu Ukrajiny”

“Una misteriosa visita in Ucraina del ministro della Difesa della Repubblica Popolare Cinese”

“Russian FM to discuss Customs Union during Ukraine visit”

“Kozhara, Lavrov to chair meeting of subcommittee on international cooperation in Chernivtsi on January 14”

“Lavrov to Discuss Ukraine’s Participation in Post-Soviet Customs Bloc”

“Ukrainian, Russian foreign ministers to discuss border-related issues in Chernivtsi”

“Yanukovych plans to meet Putin in February”

Interpretation: this cluster encompasses articles focused on diplomatic relations and meetings between Ukraine and Russia, consistently highlighted by keyphrases such as "Minister of Foreign Affairs of Russia, Sergey Lavrov," "Minister of Foreign Affairs of Ukraine, Leonid Kozhara," "Visit to Ukraine," "President of Ukraine Viktor Yanukovych," and "Meeting." The sampled headlines reflect various aspects of diplomatic engagement, including planned visits, discussions on chemistry between diplomats, and specific meetings concerning international cooperation. This demonstrates a coherent narrative across multiple sources and languages, centred on the diplomatic interactions between the two nations.
