# Supplementary material for: Tracking the narrative: A data-driven analysis of media coverage of Russia and Ukraine 2013–2024
Source: PLoS One. 2026 Jun 25;21(6):e0351627. doi: 10.1371/journal.pone.0351627 (PMC13298780; doi:10.1371/journal.pone.0351627)
Supplement: S2 Table — (DOCX) [file pone.0351627.s003.docx]

# $F_{C1}\left( x,y \right) = p_{xy}$

$$sym_{C1,C2} = \frac{1}{N_{2}}\sum_{t\in C2} F_{C1}\left( t_{x}, t_{y} \right), \left| C2 \right| = N_{2}$$

**S2 Table**. **Major news events corresponding to the peaks in media coverage shown in Figure 2.** The table associates the peaks visible in Figure 2 with the major news events to which they are related.

| **Month and year** | **News event(s) as identified in the dataset** |
| --- | --- |
| November 2013 | The Ukrainian government suspended preparations for signing the EU Association Agreement. |
| December 2013 | The Euromaidan protests, erupted on 21 November 2013, gained intensity. |
| March 2014 | Russia illegally attempted to annex Crimea following its occupation of the region and a referendum that was widely condemned as illegitimate by the international community. |
| July 2014 | The Malaysia Airlines Flight 17 was shot down. |
| February 2015 | The Minsk summit resulted in the adoption of the second Minsk Agreement. |
| June 2015 | The EU extended its sanctions against Russia. |
| March 2016 | The trial of Ukrainian pilot Nadiya Savchenko drew widespread international attention and sparked protests. |
| May 2017 | French President Emmanuel Macron met with Russian President Vladimir Putin; German Chancellor Angela Merkel held talks with Putin in Sochi; the WannaCry ransomware attack caused a major global disruption. |
| July 2017 | The G20 summit in Hamburg marked the occasion of the first meeting between US President Donald Trump and Russian President Vladimir Putin. |
| March 2018 | Vladimir Putin was re-elected as President of Russia; former Russian spy Sergei Skripal and his daughter Yulia were poisoned in Salisbury, UK. |
| November 2018 | Russia seized three Ukrainian naval vessels and their crews in the Kerch Strait. |
| April 2019 | Volodymyr Zelenskyy won the Ukrainian presidential elections. |
| December 2019 | The Normandy summit brought together leaders from Ukraine, Russia, Germany, and France to discuss the ongoing conflict in Ukraine; a new Russia–Ukraine gas transit agreement was reached. |
| April 2021 | Russia significantly increased its military presence along the Russia–Ukraine border and in the Black Sea. |
| June 2021 | The Geneva summit took place, where US President Joe Biden and Russian President Vladimir Putin met. |
| February 2022 | Russia launched a full-scale invasion of Ukraine. |
| September 2022 | Russia conducted annexation referenda in Russian-occupied territories of Ukraine, widely condemned as illegitimate by the international community; the Nord Stream pipelines were sabotaged in an attack. |
| October 2022 | An explosion severely damaged the Crimean Bridge. |
| February 2023 | The one-year mark of Russia’s full-scale invasion of Ukraine was reached. |
| May 2023 | A drone attack targeted the Kremlin. |
| June 2023 | The Kakhovka Dam was destroyed; the Wagner Group staged a rebellion against the Russian government. |
| September 2023 | The G20 summit addressed global concerns over the ongoing war in Ukraine; negotiations surrounding the Russia–Ukraine Grain Deal, expired in July 2023, took place. |
| February 2024 | Russian opposition leader Alexei Navalny was murdered. |
| March 2024 | Vladimir Putin was re-elected; a terrorist attack struck a concert hall in Moscow. |
| May 2024 | Ukraine launched strikes on Russian territory; Russian forces initiated a renewed offensive in the Kharkiv region. |
| August 2024 | Ukraine launched an offensive in the Kursk region. |
| November 2024 | Donald Trump was re-elected as US President. |
