# Supplementary material for: Tracking the narrative: A data-driven analysis of media coverage of Russia and Ukraine 2013–2024
Source: PLoS One. 2026 Jun 25;21(6):e0351627. doi: 10.1371/journal.pone.0351627 (PMC13298780; doi:10.1371/journal.pone.0351627)
Supplement: S3 Table — The table provides details on parameter settings and prompt structures for large language models. (DOCX) [file pone.0351627.s004.docx]

# $F_{C1}\left( x,y \right) = p_{xy}$

$$sym_{C1,C2} = \frac{1}{N_{2}}\sum_{t\in C2} F_{C1}\left( t_{x}, t_{y} \right), \left| C2 \right| = N_{2}$$

**S3 Table. LLMs settings.** The table provides details on parameters setting and prompts structure for large language models.

| **Max token** | 1000 |
| --- | --- |
| **Temperature** | 0 |
| **System instruction** | You are an assistant who analyses a list of texts and looks for similarities between them. Always respond in English. |
| **User prompt,**  **cluster title** | What would be the best title for the following texts that were clustered together by an AI model? Answer only with the title in English. |
| **User prompt,**  **key phrases** | Give me top 5 key phrases that the following texts have in common. Answer only with the key phrases in English and enumerate them. |
