# Supplementary material for: Tracking the narrative: A data-driven analysis of media coverage of Russia and Ukraine 2013–2024
Source: PLoS One. 2026 Jun 25;21(6):e0351627. doi: 10.1371/journal.pone.0351627 (PMC13298780; doi:10.1371/journal.pone.0351627)
Supplement: S2 Text — The monthly summaries, compiled by analysts using the clustering output and manual annotations of the top 30 clusters, present the dominant media narratives for each month of the analysed period (from January 2013 to December 2024). (DOCX) [file pone.0351627.s005.docx]

# $F_{C1}\left( x,y \right) = p_{xy}$

$$sym_{C1,C2} = \frac{1}{N_{2}}\sum_{t\in C2} F_{C1}\left( t_{x}, t_{y} \right), \left| C2 \right| = N_{2}$$

**S2 Text. Monthly summaries.** The monthly summaries, compiled by analysts using the clustering output and manual annotations of the top 30 clusters, present the dominant media narratives for each month of the analysed period (from January 2013 to December 2024).

**2013**

**January**

The news coverage highlighted Ukraine’s growing energy dependence on Russia, as ongoing disputes over gas pricing and supply prompted Kyiv to seek alternatives to Gazprom. Reports emphasised how energy policy symbolised Ukraine’s vulnerability to Russian influence and its efforts to assert greater independence.

Coverage also explored Ukraine’s geopolitical crossroads between European integration and alignment with Russia. Media outlets detailed the hesitations of Ukrainian President Viktor Yanukovych over the EU Association Agreement, a political and trade agreement between Ukraine and the EU, amid fears of Russian economic retaliation.

Corruption and political persecution featured heavily in the news, particularly in coverage of the murder investigation of Ukrainian businessman Yevhen Shcherban. Allegations against political figures, including former Prime Minister Yulia Tymoshenko, were framed as emblematic of broader governance issues under Yanukovych.

Finally, the announcement that French actor Gérard Depardieu had been granted Russian citizenship drew considerable attention, reflecting broader anxieties over Russian cultural influence and Ukraine’s quest for a distinct identity.

**February**

News coverage focused on the Russia–Ukraine gas dispute, with Ukraine seeking alternatives to Russian gas supplies, driven by Russia’s use of energy as a tool of political leverage. Reports pointed to the delicate balance Ukraine had to maintain between reducing its reliance on Russia and navigating its economic struggles.

The geopolitical tug-of-war between Ukraine, Russia, and the EU also dominated discussions. As Ukraine deliberated over the EU Association Agreement, media outlets noted that President Yanukovych’s reluctance to sign the agreement was tied to the fear of economic retaliation from Russia.

Coverage of two domestic events in Ukraine, the An-24 plane crash in Donetsk and the roof collapse at the Chornobyl nuclear plant due to heavy snow, pointed to ongoing concerns about safety and governance in Ukraine.

**March**

The Russia–Ukraine gas dispute remained a central focus. President Yanukovych met with Russian officials to discuss potential concessions, but these talks were mired in Russia’s insistence on Ukraine joining the Moscow-led Customs Union.

Ukraine’s geopolitical orientation continued to generate heated debates. Ukrainian efforts to strengthen ties with the EU were met with sharp criticism from Russian nationalist figures like nationalist politician Vladimir Zhirinovsky, who accused Yanukovych of betraying the Slavic world.

Cultural tensions surfaced in coverage of the 1025th anniversary of the Baptism of Kyivan Rus with Russian media framing Ukraine’s European ambitions as a departure from shared historical and religious traditions.

**April**

The Russia–Ukraine gas dispute remained a key issue, as Kyiv sought alternatives to Russian gas supplies, turning to Europe and exploring domestic energy solutions. Ukraine’s exploration of new energy routes along with Poland’s decision to challenge the EU exemption granted to the OPAL pipeline due to fears that the exemption would undermine the country’s energy security further highlighted the geopolitical relevance of Europe’s energy dependence on Russia.

The media marked the 30th anniversary of the Chornobyl disaster, revisiting the long-lasting impacts of the tragedy on both Ukraine and its relationship with Russia. Coverage highlighted the environmental and health repercussions, as well as the ongoing memory struggles in the region.

There was increased attention on nationalist protests within Ukraine, with reports focusing on cultural conflicts and the push for a distinct national identity.

Cultural tensions were also visible in incidents involving Russian military personnel in Sevastopol, highlighting the increasingly fraught dynamics of Russo-Ukrainian relations in Crimea.

**May**

The Russia–Ukraine gas dispute remained central, with both countries engaged in transit negotiations. As Kyiv sought to secure energy independence through alternative routes, Russia pushed for greater control over energy supplies.

Ukraine’s efforts to strengthen ties with the EU faced increasing scrutiny from Russia, which countered with offers to join its Customs Union, seeking to maintain influence over Ukraine.

Cultural and diplomatic tensions were stoked by a controversial remark from a Russian consul in Simferopol about Crimean Tatars. This incident added to the growing sense of tension over Russia’s influence in Crimea and threats over Ukraine’s territorial integrity.

Debates over LGBT rights and homophobia intensified in Ukraine, with growing protests highlighting social divisions and adding complexity to the country’s relations with both Russia and the West. These domestic tensions coincided with ongoing developments in the case of imprisoned former Prime Minister Yulia Tymoshenko, whose health and legal status continued to provoke international attention and criticism.

**June**

Ukraine’s geopolitical trajectory dominated news coverage as the government made strides toward signing the EU Association Agreement. While this move was hailed by proponents of European integration, it drew sharp criticism from Russia, which increased pressure on Ukraine to join the Moscow-led Customs Union.

The geopolitical tug-of-war extended to cultural and religious narratives, particularly during celebrations of the 1025th anniversary of the Christianization of Rus, a historic event marking the adoption of Orthodox Christianity by Kyivan Rus. Both Russia and Ukraine claimed this legacy.

The Russia–Ukraine gas dispute remained unresolved, with discussions over pipelines reflecting deeper tensions about economic and energy independence.

Protests over LGBTQ+ rights in Ukraine, spurred in part by Russia’s passage of its controversial anti-gay propaganda law, highlighted the social divide between European democratic values and conservative, Russian-influenced norms. These demonstrations symbolised a broader cultural shift as Ukraine grappled with aligning itself closer to Europe.

Yulia Tymoshenko’s imprisonment continued to draw international scrutiny, with mounting pressure on Kyiv to address human rights concerns as part of its EU integration process.

**July**

Media coverage highlighted Ukraine’s preparations for the EU Association Agreement, while concerns over domestic reforms, judicial politicisation, and Yulia Tymoshenko’s imprisonment posed barriers to integration. The EU pressed for tangible improvements, as Moscow intensified efforts to dissuade Ukraine, pushing for Customs Union membership instead.

Disputes over natural gas between Russia and Ukraine persisted, reflecting broader tensions over energy independence and economic sovereignty. This was part of a larger narrative surrounding Ukraine’s economic alignment with either Europe or Russia.

Protests continued in Ukraine concerning LGBTQ+ rights. These were partly influenced by Russia’s controversial anti-gay propaganda law, highlighting a growing cultural divide between European democratic norms and conservative, Russia-aligned ideologies.

In Russia, the sentencing of opposition leader Alexei Navalny in a corruption case drew international condemnation. The case was widely viewed as a political move by President Vladimir Putin to weaken Navalny ahead of the Moscow mayoral race, further fuelling criticism of Russia’s human rights record and the suppression of political dissent.

**August**

Media coverage focused on Ukraine’s negotiations for the EU Association Agreement. As Ukraine pushed forward with its European integration plans, Russia reacted sharply by imposing trade restrictions on Ukrainian exports, particularly targeting the agricultural sector. These measures were framed as part of Moscow’s broader strategy to coerce Ukraine into joining its Customs Union.

Energy negotiations continued, with disputes over gas imports and pricing unresolved. Talks between Kyiv and Moscow on gas supplies underscored Ukraine’s efforts to reduce dependency on Russian energy, even as economic pressures mounted.

Sociological studies on Russian public opinion revealed growing scepticism towards Ukraine and its leadership. This shift in public sentiment was amplified by the media, especially during Russia and Ukraine’s respective Independence Day celebrations and commemorations like the 70th anniversary of the Battle of Kursk and Kharkiv’s liberation from Nazi occupation during World War II.

**September**

Media coverage centred on the intensifying geopolitical tensions over the EU Association Agreement. Ukraine faced mounting pressure from the EU to implement judicial reforms and release Yulia Tymoshenko as progress on the EU deal was conditioned on such measures. Russia, on the other hand, intensified efforts to prevent Kyiv from aligning with Europe, with warnings of severe economic consequences and tighter trade restrictions if the agreement proceeded​.

Energy security remained a focal point, with the Russia–Ukraine gas dispute underlining Ukraine’s push for independence from Russian energy supplies. Reports also highlighted the implications of Greenpeace protests against Arctic oil drilling, which included Ukrainian activists, broadening the conversation on energy and environmental concerns.

Cross-border tensions flared as swine fever outbreaks prompted border closures, disrupting trade and mobility. In Moldova, an embargo on wine exports to Russia stoked fears of broader economic retaliation, especially given Moldova’s aspirations for closer EU ties.

The return of Vladislav Surkov — known for crafting Russia’s "sovereign democracy" concept, a model of governance that emphasises national sovereignty over democratic processes — to the Kremlin as an advisor signalled a potential shift in Moscow’s strategy towards Ukraine. Surkov’s influence in shaping Russia’s geopolitical manoeuvres suggested Moscow’s renewed emphasis on influencing Ukraine’s future.

**October**

Media coverage centred on Ukraine’s critical decision between deepening ties with the EU and aligning with Russia’s Eurasian Economic Union (EEU). Reports highlighted the growing pressure from Russia, which sought to persuade Ukraine to abandon the EU Association Agreement by offering a $15 billion financial package and discounted gas supplies. This move was framed as a strategic attempt by Russia to maintain its influence over Ukraine.

Another key story was the release of former Prime Minister Yulia Tymoshenko. The media framed her release, which had been a key EU demand, as a crucial step for Ukraine in its path toward European integration. However, her release also amplified internal political divisions. Pro-European media outlets celebrated it as a sign of Ukraine’s commitment to democratic reforms, while pro-Russian commentators viewed it as an example of Western interference in Ukraine’s internal politics.

Media outlets also focused on rising nationalist sentiments in Ukraine, particularly around the 69th anniversary of the country’s victory over Nazi occupation. Nationalist rallies were framed as part of a broader push to assert Ukraine’s sovereignty and independence from Russian influence. Finally, the media covered widespread public protests against government corruption and police inaction.

**November**

Media coverage centred on Ukraine’s decision to suspend the EU Association Agreement just days before it was to be signed at the Eastern Partnership summit in Vilnius. This reversal, which was widely attributed to significant pressure from Russia, sparked mass protests in Ukraine, particularly in the capital, Kyiv. The Euromaidan protests - initially small demonstrations calling for the signing of the agreement - rapidly grew into a massive pro-European movement. Media outlets across the globe reported on the pro-EU protests, framing them as a struggle for Ukraine’s future and its desire for democratic reforms, closer ties with Europe, and independence from Russian influence.

Media coverage also highlighted the political fallout surrounding President Viktor Yanukovych’s decision. The suspension of the EU deal led to calls for sanctions against the Ukrainian leadership, with Western critics accusing the government of undermining the will of the Ukrainian people. As protests spread across the country, the narrative increasingly framed the issue as one of democracy versus authoritarianism, with many Ukrainians viewing the government’s decision as an undemocratic capitulation to Russia’s political influence.

Coverage also highlighted the geopolitical divide within Ukraine. While pro-European protests erupted in the west, pro-Russian sentiment remained strong in regions like Crimea, where anti-EU marches reflected a deepening divide between Ukrainians who sought European integration and those who favoured closer ties with Moscow.

**December**

Media coverage focused on the escalating Euromaidan protests and their significance for Ukraine’s political future. The protests grew into a mass movement calling for closer ties with Europe and President Yanukovych’s resignation. Coverage framed the protests as a battle for Ukraine’s democratic future, with Western leaders condemning the government’s violent crackdown on demonstrators, while Russian officials accused the West of meddling in Ukraine’s internal affairs.

A key theme was Russia’s influence over Ukraine. Russia offered Ukraine a $15 billion financial package and gas price cuts to steer the country away from the EU and toward the Eurasian Economic Union. This was widely reported as part of Russia’s strategy to maintain its political and economic leverage over Ukraine, preventing its shift toward European integration.

The leadership of Vitali Klitschko and the Ukrainian opposition were also central in media coverage. As the protests intensified, Klitschko emerged as a prominent figure, symbolising the pro-European movement and democratic reforms. His leadership was presented as vital to shaping the future of Ukraine, with the media emphasising the importance of a unified opposition to counter Yanukovych’s government and its authoritarian tendencies.

International reactions to the crisis were a major theme. Western governments, including the EU and the US, condemned the Ukrainian government’s violent response to the protests, while Russia’s stance remained supportive of Yanukovych. This division was framed as a proxy conflict between Western ideals of democracy and Russia’s desire for regional dominance, with Ukraine caught in the middle.

**2014**

**January**

Media coverage was dominated by the escalating Euromaidan protests in Kyiv. The demonstrations, met with increasingly harsh crackdowns, underscored a growing divide within Ukraine, while Russia’s support for President Yanukovych drew widespread attention.

The Russia–Ukraine gas dispute saw a temporary resolution, with Russia reducing gas prices significantly to maintain Ukraine’s reliance on Moscow amidst its economic struggles.

Beyond Ukraine, Moldova’s Transnistria region remained a point of contention, with the media drawing parallels between its situation and the unrest in Ukraine.

The Munich Security Conference further highlighted these issues, with discussions focusing on the security implications of Russia’s assertive regional policy.

**February**

Media coverage was dominated by the escalating crisis in Ukraine, where violent clashes between protesters and security forces in Kyiv led to the ousting of President Yanukovych. Amid the political upheaval, international intervention increased, with Western powers condemning the violent crackdown and Russia framing the events as a Western-backed coup. The EU responded by imposing sanctions on Ukrainian officials, and the international community focused on supporting the post-Yanukovych government, which sought to stabilise the country amidst a volatile political and economic situation. Yulia Tymoshenko’s release from prison was widely covered, symbolising the opposition’s victory and the promise of democratic reform.

Russia’s military presence in Crimea began to escalate, with the occupation of key airports and strategic locations in Sevastopol. Protests in Crimea intensified, with ethnic Russians mobilising on one side and Crimean Tatars and Ukrainians on the other, while fears of further Russian territorial expansion continued to grow. The media highlighted the increasing likelihood of Russia’s annexation of Crimea, with some outlets even discussing the threat of nuclear escalation in response to perceived Western intervention. The growing unrest in Crimea was framed as part of Russia’s broader geopolitical strategy to assert dominance in the region and protect its naval interests in the Black Sea.

The Sochi Winter Olympics, taking place against this tense backdrop, brought additional attention to Russo-Ukrainian conflict. Ukrainian athletes were thrust into the spotlight, with doping cases overshadowing their performances. Meanwhile, media outlets reported on an incident where a Ukrainian man allegedly attempted to hijack a flight to Sochi during the Games. In a more symbolic gesture, Patriarch Kirill blessed and prayed for the Olympic teams from Russia, Ukraine, Belarus, and Moldova, underlying the complex religious and political ties between these countries.

**March**

Russo-Ukrainian relations were irrevocably altered by Russia’s annexation of Crimea. Following weeks of mounting tension and the occupation of key sites in Crimea by Russian military forces, a disputed referendum was held on 16 March. Conducted under conditions widely criticised as illegitimate, the referendum was declared to have produced results favouring accession to the Russian Federation, where most voters in Crimea and Sevastopol allegedly chose to join the Russian Federation. This move sparked widespread protests within Ukraine, particularly in the eastern regions, while the international community responded with widespread condemnation. The EU and the US imposed sanctions on Russian officials and companies in response to what was widely regarded as a violation of Ukrainian sovereignty and territorial integrity. Russia, however, framed the annexation as a restoration of historical justice and a reflection of the will of the Crimean people. The media framed the crisis as part of a broader pattern of Russian territorial ambitions.

The economic fallout from Crimea’s annexation also dominated media coverage. With Crimea now under Russian control, Ukraine lost access to key resources, particularly the Black Sea port of Sevastopol. Economic tensions between the two nations escalated, with Russia threatening to cut off natural gas supplies to Ukraine, heightening fears of an energy crisis across Europe. Meanwhile, Western nations took diplomatic measures to isolate Russia, offering economic and political support to Ukraine’s new government. Global markets fluctuated, particularly in the energy sector, amid rising fears of a wider conflict.

**April**

The conflict between Russia and Ukraine escalated dramatically, with Russian-backed forces, supported, trained, and equipped by Russia, seized government buildings in eastern cities such as Donetsk, Luhansk and Sloviansk (Delegation of the European Union to the OSCE, 2021). These actions, widely perceived as orchestrated by Moscow despite official denials, marked the start of Russia’s undeclared war against Ukraine. Ukraine’s government launched “anti-terrorism operations” to regain control, but the clashes deepened fears of a full-scale war.

The international response remained firm, with Western powers imposing additional sanctions on Russia. The EU and the US expanded punitive measures targeting Russian officials, businesses, and strategic sectors like energy and finance. NATO responded by increasing its presence in Eastern Europe, conducting military exercises, and reinforcing defences in Member States bordering Ukraine. Russia condemned these moves, accusing the West of provoking instability and framing NATO’s actions as a threat to its security.

Energy issues took centre stage during the crisis. Russia and Ukraine’s ongoing gas dispute raised concerns about European energy security, with particular attention on Germany’s heavy dependence on Russian gas.

Beyond Ukraine, the conflict had ripple effects on neighbouring regions. In Moldova, Transnistria’s renewed push for international recognition added another layer of tension, with analysts warning of parallels between the situations in Transnistria and eastern Ukraine.

**May**

The crisis in Ukraine intensified as violence and political instability gripped the nation. Russian-backed forces held referenda in Donetsk and Luhansk on 11 May, which were condemned by Kyiv and the international community as illegitimate. The Ukrainian government intensified its military operations against the Russian-backed forces, leading to deadly clashes in several cities.

The media also focused on Ukraine’s presidential election, held on 25 May, which aimed to restore political stability. Petro Poroshenko emerged as the victor, pledging to end the conflict in the east and strengthen Ukraine’s ties with the West. However, the election took place under the shadow of violence, with regions under Russian occupation refusing to participate and accusations of Russian interference looming large.

The EU and the US expanded sanctions on Russia, while NATO increased military readiness in Eastern Europe. Russia, meanwhile, rejected the legitimacy of Ukraine’s military actions in the east, accusing Kyiv of waging war on its own people.

Energy politics continued to dominate discussions, particularly as the gas dispute between Russia and Ukraine raised concerns across Europe. The European Commission’s winter economic forecast pointed to increased external risks to the EU’s economic stability, driven in part by the escalating conflict.

**June**

Media coverage centred on Ukraine’s military efforts to reclaim territories under Russian control, with the recapture of Mariupol widely reported as a pivotal moment in Kyiv’s campaign. The conflict in Donetsk and Luhansk was framed by allegations of Russian arms supplies to separatists, with Ukrainian and Western officials accusing Moscow of covert support.

The humanitarian crisis drew significant attention, with coverage detailing the displacement of thousands of Ukrainians, many of whom sought refuge in Russia. Human rights organisations raised concerns about violations committed by both sides, including indiscriminate shelling and attacks on civilian infrastructure. Investigations into mass killings during the Maidan protests also made headlines, linking former Ukrainian President Yanukovych and officials to alleged criminal schemes.

International sanctions against Russia remained a key focus, as the EU and the US introduced new measures targeting strategic sectors and prominent individuals. The media framed these sanctions as a key component of a broader Western strategy to isolate Moscow diplomatically and economically.

Energy politics dominated headlines, with particular emphasis on the gas dispute between Russia and Ukraine. Concerns about European energy security were amplified by discussions surrounding the South Stream Gas Pipeline, which faced construction suspension due to EU objections. The European Commission urged Member States to diversify energy sources.

**July**

Media coverage was dominated by the downing of Malaysia Airlines Flight MH17 over eastern Ukraine, which killed all 298 people on board. International investigations quickly pointed to a surface-to-air missile launched from separatist-held territory, with US intelligence implicating pro-Russian forces. The incident intensified global condemnation of Russia’s role in the conflict, leading to calls for accountability and heightening geopolitical tensions.

Western nations expanded sanctions against Russia, with the EU widening its sanctions list to include more officials and entities.

The conflict in eastern Ukraine remained a focal point, as Ukrainian forces sought to regain control of separatist strongholds. Reports of human rights violations, including torture and kidnapping, by armed groups in eastern Ukraine drew attention from organisations like Amnesty International.

Energy politics continued to feature prominently, with the gas dispute between Russia and Ukraine exacerbating European concerns about energy security.

**August**

Media coverage focused on the worsening humanitarian crisis in eastern Ukraine, particularly in the conflict zones in eastern Ukraine, with increasing reports of civilian casualties, displacement, and widespread destruction. International organisations, including the UN, highlighted the mounting human rights violations, including reports of torture and killings, perpetrated by both Ukrainian forces and Russian-backed forces.

A major focus in the media was Russia’s controversial “humanitarian aid” convoy sent into eastern Ukraine. While Russian authorities presented the convoy as a measure to alleviate civilian suffering, the international media was divided, with Western outlets treating it as a political provocation. There was widespread suspicion that the convoy could be a pretext for military operations, with allegations suggesting that weapons were hidden within the supplies.

The economic fallout from the conflict also dominated discussions, particularly in Europe. The EU’s reliance on Russian natural gas remained a critical issue as the threat of disruptions in supply continued to rise. Russian import bans on food products and the potential for further disruptions in gas supplies contributed to fears of economic instability across the continent.

At the same time, Western governments continued to expand sanctions against Russia. These measures were aimed at isolating Russia economically and politically, but the impact on both Russian and European economies was increasingly evident.

**September**

It was widely reported that a ceasefire agreement, known as the *Minsk Protocol*, between Ukraine and the pro-Russian rebels had been reached on 5 September. However, the media emphasised the fragile nature of this truce, with frequent violations and little progress towards lasting peace.

The NATO summit held in Newport, Wales, from 4 to 5 September also attracted significant media attention. Leaders at the summit agreed to implement additional sanctions on Russia, accusing Moscow of deploying several thousand troops inside Ukraine. Media coverage focused on the EU’s sanctions against Russia, highlighting how the sanctions impacted both Russian and European economies.

The investigation into the downing of MH17 continued to generate media attention, with reports indicating that Russian-backed forces were blocking access to the crash site and Moscow’s role in supporting separatists under scrutiny.

The gas dispute between Russia and Ukraine was also a prominent issue, with attention on Europe’s dependence on Russian natural gas. As winter approached, European efforts to diversify their energy sources became more urgent. Media outlets reported on Qatar’s role in supplying gas to Europe, attempting to reduce reliance on Russian energy.

**October**

Media coverage centred on the gas dispute between Russia and Ukraine, with EU officials working to mediate the energy supply crisis. Reports highlighted concerns about European energy security, especially as the risk of a winter blackout loomed.

The Minsk Agreement continued to be a focal point in diplomatic discussions, but media coverage showed that peace efforts remained stalled, with military clashes ongoing in eastern Ukraine. While the EU and other international actors pushed for a resolution, reports indicated limited progress in the implementation of ceasefire terms, and Russia’s indirect role in supporting separatist forces remained a contentious issue.

Ukraine’s parliamentary elections took place in late October. Media outlets reported on international concerns regarding the legitimacy of elections held in Russian-controlled regions, such as Donbas and Crimea.

The humanitarian impact of Russia’s aggression was another significant focus in the media, particularly the refugee crisis (Follis, 2022). Thousands of Ukrainians sought temporary relocation in Russia, and reports detailed the challenges faced by both the displaced population and the host countries, highlighting the strain on resources and infrastructure.

Finally, reports on cyberattacks attributed to Russia were widely covered, with media outlets discussing how Russian hackers exploited vulnerabilities in Windows systems to target NATO, European governments, and energy companies. These incidents raised concerns about the broader implications for cybersecurity and the potential for escalation in the ongoing conflict.

**November**

Media coverage focused on the war in eastern Ukraine, where the separatist crisis remained unresolved despite international diplomatic efforts. Media outlets discussed the growing military confrontation and the challenges in implementing the Minsk Agreement, with Russian-backed separatists continuing to hold territory in the Donbas region.

As the war dragged on, the sanctions imposed on Russia by the EU and other Western countries remained a central narrative. Media reports analysed the economic impact of these measures, noting the combined effects of the sanctions and falling oil prices, which contributed to a currency crisis in Russia and economic uncertainties across the Eurozone.

Humanitarian concerns were also widely covered, particularly the refugee crisis sparked by the war. Reports focused on the human rights issues and the “humanitarian convoy” sent by Russia to eastern Ukraine, with Western outlets accusing Moscow of using the cargo as a cover for military support to separatist forces.

**December**

Media coverage analysed Ukraine’s decision to abandon its non-aligned status and pursue NATO membership. The growing security tensions between Russia and the West were frequently mentioned, with media outlets noting the deteriorating relations between Russia and the EU.

Sanctions remained a key narrative, with media coverage focusing on the economic impact of Western sanctions on Russia. The media explored Putin’s shifting foreign policy, particularly his increased interest in Asia, as Russia looked to diversify its economic partnerships, with LNG exports to India cited as a potential new avenue for growth.

The humanitarian impact of the conflict continued to dominate the media narrative. Reports highlighted the refugee crisis, with thousands of Ukrainians seeking safety in other regions of Ukraine and abroad.

Media also discussed proposals for Ukraine to create a Ministry of Information Policy, aiming to manage information and counter propaganda in the context of Russia’s aggression against Ukraine. The issue of FIMI was further explored with a focus on online trolls and disinformation campaigns aimed at influencing public opinion in countries like Finland.

Finally, there was some attention on Ukrainian nuclear power plant incidents, which were framed as further evidence of the conflict’s destabilising impact on infrastructure and safety in Ukraine.

**2015**

**January**

The media extensively covered the intensifying conflict in eastern Ukraine, where Russian-backed forces ramped up offensives against Ukrainian forces. Russian President Vladimir Putin exacerbated tensions by accusing the Ukrainian army of functioning as a "NATO legion," framing the conflict as a direct threat to Russian sovereignty.

Diplomatic efforts dominated headlines, particularly around the Minsk Agreement negotiations and international mediation. Talks in Berlin involving Ukraine, Russia, Germany, and France sought to revitalise the Minsk Protocol, but disagreements over ceasefire violations stalled progress. Ukrainian Prime Minister Arseniy Yatsenyuk’s visit to Berlin was widely reported as a call for Western unity and pressure on Russia to uphold the agreements.

Economic narratives focused on the impact of Western sanctions on Russia, including calls from US Vice President Joe Biden for increased measures. The media explored Russia’s exclusion from the SWIFT international payment system as a potential sanction, sparking debate on its implications. Expert analyses debated whether these measures would deter Moscow’s actions in Ukraine.

Reports also highlighted Ukraine’s energy crisis, with coal supply disruptions and concerns over Russian gas exports affecting domestic stability and European energy security.

International responses to Russian propaganda also featured prominently. European governments implemented countermeasures to combat disinformation, framing it as a tool of hybrid warfare.

**February**

Media coverage focused on intensified diplomatic efforts to address the conflict in eastern Ukraine. The Minsk II Agreement, brokered by Germany, France, Russia, and Ukraine, was a central narrative. The accord, aimed at halting hostilities, included measures such as a ceasefire, withdrawal of heavy weaponry, and constitutional reforms in Ukraine. However, reports highlighted persistent violations almost immediately after the agreement’s signing.

The humanitarian crisis remained a significant focus, with the UN estimating over 1.5 million internally displaced persons (IDPs). The media detailed the severe conditions faced by civilians in war zones, compounded by shelling and disrupted access to humanitarian aid. Controversy over Russian “humanitarian convoys” into separatist-held territories continued, with Western governments questioning their purpose and transparency.

Reports on Western sanctions against Russia explored their impact, particularly considering the continued fighting despite Minsk II. Discussions about excluding Russia from the SWIFT international payment system gained traction but were met with caution due to potential global economic repercussions.

International responses to Russian propaganda remained a focal point. European governments intensified counter-disinformation measures, labelling Russian media campaigns as part of a broader hybrid warfare strategy.

**March**

Media coverage highlighted the continued violation of the Minsk II Agreement, with skirmishes in areas like Shyrokyne and Donetsk airport contributing to a death toll exceeding 6,000. International organisations warned of the worsening humanitarian crisis, with millions displaced and limited access to aid.

The one-year mark of Russia’s annexation of Crimea featured prominently in coverage. Analysts explored the political, economic, and military implications of the annexation, with Western governments reaffirming their condemnation of the move as illegal.

The assassination of Russian opposition leader Boris Nemtsov sparked widespread protests and mourning, with thousands gathering in Moscow for a memorial march. International media framed the murder as emblematic of the risks faced by Kremlin critics and raised questions about Russia’s political climate under President Vladimir Putin. The investigation into Nemtsov’s death attracted global scrutiny amid claims of limited transparency.

The downing of MH17 resurfaced in news coverage. Investigations linked separatist communications and the deployment of a Russian-made Buk missile system to the tragedy, prompting renewed calls for accountability.

NATO’s role in addressing Russia’s aggression against Ukraine remained a focal point, with the alliance increasing troop deployments and military exercises in Eastern Europe. These actions were framed as both deterrence and reassurance for Member States concerned about Russian aggression.

**April**

Media coverage highlighted the conflict’s international dimensions. The EU escalated its stance against Russia by accusing Gazprom of abusing its market dominance in Central and Eastern Europe, signalling energy security as a key battleground in the geopolitical standoff. At the same time, Ukraine sought to reduce its reliance on Russian gas, increasing imports from global energy companies.

NATO’s presence in Eastern Europe, including US military training missions in Ukraine and the Baltics, was another focal point. Coverage contrasted Russia’s portrayal of these actions as provocations with Eastern European nations’ framing of them as essential deterrents against potential aggression.

The assassination of pro-Russian journalists and political figures in Ukraine, notably Oles Buzyna, drew significant attention. The media framed these events as indicative of rising internal tensions and highlighted concerns over freedom of expression and rule of law in the war-stricken country.

The investigation into the downing of MH17 resurfaced, with media continuing to explore the involvement of separatists and potential Russian culpability.

**May**

Media coverage focused on the Eastern Partnership summit, which underscored the EU’s commitment to fostering relations with post-Soviet states, despite Russian opposition.

NATO and Nordic air forces conducted Arctic exercises, which Moscow criticised as provocative, while Eastern European nations continued to advocate for robust Western support amid growing concerns over Russian aggression.

Sanctions remained a contentious topic, with Russia banning entry to 89 European politicians in a retaliatory move. The economic impact of sanctions and disputes involving Gazprom featured prominently, reflecting the geopolitical and financial stakes of the conflict.

Energy disputes further shaped the media narrative, with the revival of the South Stream pipeline project generally portrayed as a strategic move by Russia to bypass Ukraine.

The investigation into the MH17 tragedy continued to draw attention, with a Russian report questioning the involvement of pro-Russian separatists and suggesting that the aircraft was downed by a Ukrainian Buk missile system. This report contradicted the findings of international investigators, leading to controversy and accusations of disinformation.

**June**

Media coverage focused on the EU’s decision to extend sanctions against Russia, reflecting sustained international pressure over Moscow’s involvement in the conflict. Reports emphasised the economic strain on Russia, yet also noted President Vladimir Putin’s enduring domestic popularity.

Energy disputes between Russia and Ukraine remained prominent, with the media highlighting ongoing negotiations over gas supplies. Coverage detailed the broader implications for European energy markets.

The escalating military posturing between NATO and Russia was another key focus. Media outlets reported on Putin’s announcement of an expanded Russian nuclear arsenal, which prompted strong responses from NATO, including military exercises in Eastern Europe.

The investigation into the MH17 crash reemerged in the media. Reports scrutinised Russia’s role, with allegations of evidence manipulation and the Dutch government engaging in talks with Russian officials about the investigation.

Finally, media attention turned to the worsening migrant crisis, with narratives linking displacement from the conflict in Ukraine to broader European migration challenges.

**July**

Media coverage reflected on the one-year mark of the MH17 crash, with the investigation and international calls for justice taking centre stage. Reports focused on the push to establish a UN-backed international tribunal, which was vetoed by Russia in the Security Council. Media outlets scrutinised Moscow’s stance, highlighting international criticism and the broader implications for justice and accountability in the conflict in Ukraine.

The escalating violence in eastern Ukraine was another key narrative, with reports on intensified clashes between Ukrainian forces and separatist groups in the Donbas region. The humanitarian fallout of the conflict also featured prominently, particularly the urgent need for HIV medication in war-torn areas, where shortages threatened the lives of thousands of patients.

Energy security remained a topic of concern as Russo-Ukrainian gas disputes resurfaced, with media discussing the potential impact on European markets.

Economic relations between the EU, Russia and Ukraine also received attention, including the effects of sanctions and the Russian import ban on European agriculture.

**August**

Media coverage highlighted the escalating violence in eastern Ukraine, with reports detailing ongoing clashes, ceasefire violations and rising casualties in the Donbas region. Diplomatic efforts to implement the Minsk Agreements were extensively reported, but progress remained limited.

The energy dispute between Russia and Ukraine continued to feature prominently, with media outlets analysing Gazprom’s Turkish Stream pipeline project. Discussions focused on the financial and geopolitical implications of the proposed construction, particularly its potential to bypass Ukraine as a transit country for Russian gas.

The MH17 investigation also returned to the forefront, with renewed international calls for establishing a tribunal to prosecute those responsible for the crash.

Western sanctions against Russia remained a central narrative. Reports explored the economic repercussions of sanctions and Russia’s retaliatory food embargo, including the destruction of banned Western imports. Analysts debated the impact of these measures on Russia’s economy and the resilience of President Vladimir Putin’s administration.

**September**

Media coverage highlighted the extension of EU sanctions on Russia and Ukraine’s own measures, which targeted Russian individuals and journalists tied to the annexation of Crimea and Russia’s aggression in Donbas.

The Donbas conflict remained in focus, with ceasefire violations and media restrictions in separatist-controlled areas underscoring the ongoing tensions. Meanwhile, discussions on implementing the Minsk Agreements pointed to slow progress on disarmament and conflict resolution.

Energy disputes between Russia and Ukraine surfaced in discussions around securing winter gas supplies. Media coverage detailed the tripartite agreement involving Russia, Ukraine, and the EU to ensure energy stability during the colder months. The Nord Stream 2 pipeline project prompted debates over Europe’s dependence on Russian energy.

The humanitarian crisis linked to the conflict was also a focal point. Reports analysed the refugee situation in Eastern Europe and Ukraine, framing it within the broader European refugee crisis.

Ukraine’s adoption of a new military doctrine, which identified Russia as its primary threat, was seen as a step towards closer NATO alignment.

**October**

Media coverage highlighted Russia’s growing military involvement in Syria, with analysts drawing parallels between Russia’s intervention strategies in Syria and Ukraine.

Reports underscored the ceasefire under the Minsk Agreements, with journalists noting limited progress against the backdrop of a low-intensity conflict in eastern Ukraine. Former Ukrainian President Leonid Kuchma’s warnings about the Donbas conflict becoming a “frozen conflict” like Transnistria were widely cited as emblematic of the geopolitical deadlock.

The investigation into the MH17 crash dominated headlines, with Dutch authorities confirming that a Russian-made missile launched from an area of eastern Ukraine controlled by Russian and separatist forces was responsible. Media outlets focused on the implications for Russia’s international standing and growing calls for accountability.

Economic sanctions also received significant attention, with outlets analysing their deepening impact on Russia’s struggling economy. Ukraine’s decision to impose its own restrictions, including bans on Russian airlines, further escalated tensions. Coverage of gas supply negotiations between the two countries revisited concerns about energy security as winter approached.

Local elections in Ukraine drew international scrutiny, with reports focusing on separatist-held territories’ decision to postpone voting, a move seen as undermining the peace process.

**November**

Media attention focused on the escalating energy crisis, primarily stemming from the gas dispute between Russia and Ukraine. Gazprom’s decision to halt gas supplies to Ukraine over unpaid pre-payments was a significant development, heightening tensions in the region. The crisis impacted not only Ukraine but also neighbouring Bulgaria, which enacted emergency measures in response. The energy shortage in Crimea, compounded by electricity supply disruptions and protests, also drew significant coverage.

The conflict in eastern Ukraine remained a key point of discussion, with reports on border incidents and Ukraine’s temporary ban on goods transport to Crimea highlighting the ongoing hybrid warfare. Meanwhile, Ukraine’s food embargo against Russia and its ban on Russian aircraft transit emerged as significant points of tension.

The international response to Russia’s involvement in both Ukraine and Syria also received significant attention. NATO’s reaction to the tensions between Ukraine, Russia, and Turkey, following Turkey’s downing of a Russian warplane near the Turkish-Syrian border, highlighted the growing geopolitical stakes. Turkey claimed the aircraft violated its airspace, while Russia denied the accusation, further deepening the rift between NATO allies and Russia amid their competing interests in the Syrian conflict. These tensions sparked broader discussions about the global implications of the Russia-Turkey standoff, with fears mounting over the potential for wider conflict.

The Minsk Agreements and their implementation remained under scrutiny, with the Normandy Format meetings — consisting of France, Germany, Ukraine, and Russia — seen as a critical avenue for potential resolution.

**December**

Reports highlighted mutual accusations between Russia and Ukraine: Russia blamed Ukraine for sabotaging power lines to Crimea, while Ukraine accused Russia of orchestrating a cyberattack on its power grid, heightening concerns over energy security and hybrid warfare tactics.

Trade and sanction disputes received significant attention, with the EU and US expanding sanctions against Russian entities and individuals. Media coverage centred on Ukraine’s implementation of a free trade agreement with the EU, which prompted Russia to retaliate with an embargo on Ukrainian goods.

The Minsk Agreements remained a focal point, with international coverage stressing escalating ceasefire violations in eastern Ukraine. These violations fuelled concerns about Russia’s involvement in the conflict and the need for compliance with the agreements, as highlighted during diplomatic efforts under the Normandy Format.

Meanwhile, the interplay between the conflict in eastern Ukraine and the broader Syrian crisis continued to shape international responses. Reports highlighted NATO’s challenges in balancing a firm stance against Russia’s actions in Ukraine with addressing the Kremlin’s growing influence in Syria. This dual focus complicated NATO’s messaging, as it sought to reassure Ukraine of its unwavering support while navigating geopolitical tensions stemming from Russia’s military operations in the Middle East. Analysts underscored how these overlapping crises tested NATO’s cohesion and strategic priorities.

**2016**

**January**

News coverage focused on Ukraine’s imposition of a trade embargo on Russian goods in response to Russian restrictions on Ukrainian exports. This move was framed as a sign of Kyiv’s determination to realign its economic ties away from Moscow and toward the EU.

Energy independence emerged as another dominant theme, with Ukraine continuing its efforts to reduce reliance on Russian gas. This discussion was closely tied to broader European debates about energy security, including the Nord Stream 2 pipeline and its implications for Ukraine’s role as a key transit country for Russian gas to Europe.

Crimea remained at the centre of media reporting, with coverage highlighting energy shortages in the region and the continued international refusal to recognise Russia’s annexation. Controversies, such as Coca-Cola’s depiction of Crimea as part of Russia on a map, further revealed the sensitivity surrounding the peninsula’s status.

The implementation of the Minsk Agreements also remained a key focus, with reports highlighting the lack of substantial progress in the diplomatic talks between Ukraine, Russia, and international mediators. The persistence of ceasefire violations and mutual accusations underscored the deep mistrust between the parties and the difficulty of achieving lasting peace.

Reports of cyberattacks targeting Ukraine’s infrastructure, including power grid disruptions, pointed to Russia’s use of hybrid warfare tactics.

**February**

The conflict in eastern Ukraine remained a critical focus, with reports on prisoner exchanges between Ukraine and separatist forces. These exchanges were framed as small but significant steps toward addressing the human toll of the conflict, although broader ceasefire violations and the lack of meaningful progress in implementing the Minsk Agreements dampened hopes for resolution.

International reactions to Russia’s aggression against Ukraine were featured at the Munich Security Conference, where world leaders discussed the broader implications of Russia’s actions in Ukraine, Crimea, and Syria, emphasising the interconnected nature of these crises. Diplomatic efforts, including phone discussions between Russian President Vladimir Putin and US President Barack Obama, highlighted attempts to address both the Ukrainian and Syrian conflicts.

Cultural and historical dimensions of the conflict also garnered attention. Ukraine’s Eurovision entry, a song by a Crimean Tatar singer about Stalin-era deportations, became a symbolic act of resistance against Russia’s annexation of Crimea. The song’s focus on historical trauma resonated with Ukraine’s contemporary struggles for sovereignty and justice. This narrative was further amplified by ongoing reports of human rights abuses in Crimea, particularly the repression of Crimean Tatars, which drew condemnation from international human rights organisations like Amnesty International.

Sanctions against Russia remained a contentious topic, with mixed messages emerging from Europe. While some groups, particularly in Germany, advocated for easing sanctions to promote economic recovery, others supported maintaining pressure on Moscow. These debates underscored the complexity of balancing economic interests with geopolitical principles in addressing the conflict.

**March**

A major theme in the news was the conflict in eastern Ukraine, where ceasefire violations continued to undermine the Minsk Agreements. Diplomatic efforts to find a sustainable resolution and stabilise the region, including through local elections in Donbas, showed little progress.

The Russian sentence of 22 years in prison for Ukrainian pilot Nadiya Savchenko, accused of complicity in the deaths of two Russian journalists in the war zone, also gathered significant media attention. News outlets reported that her trial was politically motivated, prompting protests from Ukraine and its allies, with demonstrations outside Russian embassies and consulates.

Debates over sanctions against Russia persisted, with some European officials emphasising the importance of maintaining a unified stance on sanctions, despite voices advocating for their easing to improve economic ties with Russia.

The construction of the Kerch Strait Bridge, connecting Russia to Crimea, was presented as a symbol of Russia’s consolidation of control over the illegally annexed peninsula. Kyiv and its allies criticised the Crimean bridge project as a violation of Ukraine’s sovereignty.

**April**

A significant portion of media reporting focused on the results of the Dutch referendum on the EU-Ukraine Association Agreement. The Dutch public’s rejection of the agreement was framed as a sign of scepticism about deeper integration with Ukraine. Analysts linked the outcome to broader concerns about EU expansion and the role of Russian propaganda in shaping public opinion.

Nadiya Savchenko’s case continued to dominate headlines. Following her controversial conviction in March, discussions in April shifted towards her potential extradition to Ukraine. Media reports described ongoing diplomatic and legal manoeuvres surrounding her case, while activists used her situation to highlight broader human rights abuses under Russian authority.

The debate over the Nord Stream 2 pipeline underscored the intersection of energy policy, economics, and geopolitics. Critics of the project, including Ukraine, argued that it would undermine European energy security by increasing dependence on Russian gas while bypassing Ukrainian transit routes. The issue fuelled tensions among EU Member States with divergent energy priorities and heightened the geopolitical conflict between Russia and Ukraine.

Diplomatic efforts to address the conflict persisted, with the NATO-Russia Council meeting for the first time since 2014 to address Ukraine’s security situation and broader tensions in Eastern Europe. Reports highlighted NATO’s concerns about Russian military activities and the Alliance’s steps to reinforce its Eastern flank. Meanwhile, the G7 foreign ministers’ meeting in Hiroshima reaffirmed international commitments to supporting Ukraine.

The 30th anniversary of the Chornobyl disaster provided a moment for reflection on the historical and ongoing impact of the event. Ukrainian officials used the commemorations to draw parallels between the disaster’s enduring legacy and contemporary threats posed by Russia’s actions in Crimea and eastern Ukraine. These comparisons emphasised the vulnerability of Ukraine’s infrastructure and the potential risks of military conflict in a region with significant nuclear facilities.

**May**

The release of Ukrainian pilot Nadiya Savchenko from a Russian prison in a prisoner swap, after nearly two years in detention on charges of involvement in the killing of two Russian journalists, was widely reported as a symbolic victory for Ukraine. Analysts speculated on the potential implications of her release for the broader conflict in eastern Ukraine.

Media reports highlighted the challenges of holding local elections in Donbas and the ongoing economic blockade of the war-affected region. In a further deterioration of bilateral ties, Ukraine banned all Russian airlines from flying to its territory, mirroring Russia’s earlier ban on Ukrainian carriers. Meanwhile, investigations into Ukrainian citizens allegedly financing terrorist organisations in Donbas also attracted media attention.

International responses to the conflict featured prominently in news coverage, particularly the extension of EU sanctions against Russia. Reports also examined Russia’s countermeasures, including its extended food embargo, and their impact on EU farmers.

The Eurovision song contest, won by Ukraine, became a significant political and cultural moment. Media coverage highlighted the political controversy surrounding Ukraine’s winning entry and speculated on its broader implications.

**June**

News coverage focused on the conflict in eastern Ukraine, with reports highlighting an escalation of violence and continued concerns over Russia’s aggression. The EU’s response, particularly the extension of sanctions against Russia, was widely covered, with some outlets noting divisions within the bloc.

NATO-Russia relations also featured prominently in media reports. Coverage focused on NATO’s military preparedness in the Baltic region and its role in the broader conflict, while Ukraine and Georgia’s push for NATO membership was discussed as a countermeasure to Russian aggression.

Meanwhile, the St Petersburg Economic Forum attracted attention, as European leaders such as Jean-Claude Juncker and Matteo Renzi met with Vladimir Putin to discuss economic cooperation and potential ways to ease tensions despite ongoing sanctions.

Religious tensions surfaced in the news, with coverage of conflicts within the Orthodox Church related to Ukraine’s request for autocephaly, underscoring broader geopolitical and cultural divides between Kyiv and Moscow.

**July**

The NATO summit in Warsaw was a major focus of news media, with discussions centred on the alliance’s response to Russian actions in Ukraine and the reinforcement of its eastern flank. Coverage analysed the summit’s implications for Ukraine-NATO cooperation, European security, and prospects for conflict resolution in the region.

The conflict in eastern Ukraine remained a key topic, with media highlighting escalating violence, civilian casualties, and reported abuses by all parties involved. International responses, including joint reports by Amnesty International and Human Rights Watch, were widely covered.

US-Russia relations featured prominently in news coverage, with discussions on Donald Trump’s stance on NATO and Russia, diplomatic tensions, and reciprocal expulsions between Washington and Moscow. Media reports also speculated on the potential impact of the US presidential election on global security and US-Russia relations.

The aftermath of the Brexit referendum was analysed in the context of European political shifts, with some commentators suggesting that Russia could benefit from the resulting instability.

**August**

Tensions between Russia and Ukraine escalated, particularly over Crimea. Russia accused Ukraine of planning acts of sabotage and terrorism to destabilise the peninsula, prompting Moscow to heighten military alerts along the Crimean border and tighten security measures. These developments triggered strong international reactions and diplomatic efforts to defuse the crisis.

At the G20 summit in Hangzhou, China, world leaders, including Putin, Merkel, and Hollande, debated the conflict in eastern Ukraine alongside broader global security concerns.

Meanwhile, the gas dispute between Russia and Ukraine intensified, with officials negotiating energy relations, gas supply agreements, and transit conflicts. The proposed TurkStream pipeline added another layer of complexity, raising fresh concerns over Europe’s energy security.

In the US, the presidential election campaign fuelled controversy, as critics accused Russia of attempting to influence the race. Scrutiny mounted over Trump’s ties to Ukraine’s pro-Russian political circles, particularly in relation to alleged corruption and connections to former Ukrainian President Yanukovych.

**September**

Intensified military activity in eastern Ukraine, with direct Russian involvement, dominated news coverage. European diplomats continued efforts to resolve the conflict, with German and French foreign ministers visiting Russia to push for progress on implementing the Minsk Agreements. German Vice Chancellor Sigmar Gabriel met with Russian President Vladimir Putin, discussing trade, sanctions, and conflict resolution — talks that analysts viewed as a significant development.

Sanctions remained a key theme, as the EU extended its measures against Russia and the US imposed new ones on Russian companies involved in building the Crimean Bridge. Many reports framed these moves as an escalation of Western pressure on Moscow.

The international investigation into the MH17 crash gained renewed attention, with findings identifying a Russian-made Buk missile as the cause. Russia rejected the conclusions, denying any involvement.

The upcoming elections in the US, Russia, Belarus, and Ukraine also generated significant discussion. Russia pushed for elections in occupied Donbas, sparking debate over Ukraine’s stance on the issue. Coverage of Russia’s Duma elections focused on political repression and the country’s tense international standing. In the US, Trump’s praise for Putin and the potential implications for foreign policy fuelled further controversy.

**October**

Media coverage centred on the Normandy summit, where the leaders of Russia, Ukraine, France, and Germany met to discuss the conflict in eastern Ukraine and the Syrian conflict. The summit focused on implementing the Minsk Agreements.

Tensions over the gas dispute remained high, with reports on energy supply issues, European energy security, and controversies surrounding the Russia-Germany gas pipeline, as well as the Turk Stream and South Stream projects, underscoring the complex energy dynamics between Russia, Ukraine, and Europe.

The MH17 investigation gained renewed attention with reports identifying suspects and further implicating Russia. The debate over Russian responsibility was highlighted by a British resident’s call on LBC Radio urging an end to "anti-Russian propaganda" during a discussion on the case.

The impact of sanctions on Russia remained a focal point, particularly Serbia’s controversial appointment of Putin’s sanctioned ally Gennady Timchenko as honorary consul in St Petersburg. Ukraine also expanded its own sanctions list against Russian individuals and entities.

**November**

Media coverage focused on the conflict in eastern Ukraine, with reports highlighting sabotage allegations, clashes between Ukrainian and Russian-backed forces, and concerns over missile tests near Crimea. The criminal investigation into threats against the Zaporizhzhia nuclear power plant underscored fears of broader security risks. Diplomatic efforts to resolve the conflict were discussed in the context of the incoming Trump administration, with speculation on potential shifts in US policy.

The energy dispute remained prominent, with coverage of gas transit issues, European energy cooperation, and Gazprom’s continued investment in the Turkish Stream pipeline.

Sanctions against Russia were extended, with Western leaders weighing their effectiveness and the possibility of policy changes under the Trump presidency.

The victory of pro-Moscow candidate Igor Dodon in Moldova’s presidential election was framed within broader East-West tensions, highlighting challenges in EU-Russia relations.

Growing fears of Russian interference in European affairs were underscored by reports on cyberattacks against the European Commission, discussions at the EU-Ukraine summit, and efforts to counter disinformation and propaganda.

**December**

Media coverage continued to highlight rising tensions, including military exercises near Crimea and espionage cases. A Russian court’s formal recognition of the 2014 Ukrainian revolution as a coup, implicating former President Viktor Yanukovych and Russian officials, reinforced Moscow’s narrative.

The extension of EU sanctions against Russia for another six months was widely reported, with discussions focusing on its implications for the Minsk Agreements and the ongoing gas dispute.

US-Russia relations remained under scrutiny in the aftermath of the US presidential election, with the media assessing sanctions, cyber tensions, and the broader geopolitical impact of a Trump presidency. Ukraine’s European integration was a key topic, with news reports on the EU-Ukraine Association Agreement and the Dutch ratification process, which helped solidify the treaty despite earlier concerns. The Russian election interference narrative remained prominent, with Trump’s denial of Russian meddling in the US election contrasted against CIA reports and congressional investigations pointing to the contrary. Concerns about Russian interference extended to Europe, with fears of information manipulation in Germany’s 2017 Bundestag campaign reinforcing anxieties over FIMI.

**2017**

**January**

Media coverage centred around the escalation of the conflict in eastern Ukraine, particularly around the town of Avdiivka. Media reports highlighted the intensity of the clashes between Ukrainian and Russian-backed forces, which resulted in casualties, injuries, and a deepening humanitarian crisis. More than 200 miners were trapped underground, drawing further attention to the dire conditions on the ground.

Sanctions against Russia continued to dominate international discourse. The outgoing Obama administration imposed new sanctions targeting Russian officials, accusing them of human rights abuses and involvement in the murder of former Russian spy Alexander Litvinenko. Vice President Joe Biden’s final visit to Ukraine was widely covered, with Biden emphasising the importance of continued Western support for Ukraine and maintaining sanctions on Russia. However, within Europe, the sanctions issue sparked controversy, particularly in Hungary, where the government criticised the EU’s approach as economically damaging and politically ineffective.

As President Donald Trump took office in Washington DC, speculation about his administration’s stance on Russia dominated media coverage. A phone conversation between Trump and Russian President Vladimir Putin raised concerns in Europe and among US lawmakers, with many questioning whether Trump would seek to ease sanctions on Russia. This uncertainty was juxtaposed with NATO’s ongoing military presence in Eastern Europe, exemplified by the deployment of US troops to Poland, a move seen as reinforcing NATO’s commitment to regional security in the face of continued Russian aggression.

Ukraine’s legal and political responses to Russia’s actions remained in the spotlight. The Ukrainian government initiated proceedings against Russia at the International Court of Justice, accusing Moscow of violating international conventions with its temporary occupation and attempted annexation of Ukrainian territories. The trial of former Ukrainian President Viktor Yanukovych, who was charged with treason for his role in the 2014 crisis, also captured significant media attention, illustrating the legal and political challenges Ukraine faced in its post-Maidan transformation.

**February**

The conflict in eastern Ukraine remained at the forefront. Intense fighting continued, particularly in Avdiivka. The international community responded with calls for renewed commitment to the Minsk Agreements, though the conflict persisted with no clear resolution in sight.

NATO’s role in Eastern Europe and its relationship with the Trump administration became a key theme. The uncertainty surrounding US foreign policy under President Trump was widely discussed, particularly regarding sanctions and relations with Russia. Trump’s softer stance towards Russia raised concerns in Europe, prompting questions about NATO’s future direction and the strength of transatlantic relations. The strategic importance of NATO’s presence in Eastern Europe and the Black Sea region was underscored in media coverage, as commentators and analysts highlighted NATO’s role in deterring Russian aggression.

Sanctions against Russia remained a contentious issue, with reports emerging of Moscow seeking to circumvent EU sanctions, particularly regarding gas turbines in Crimea. The Kremlin’s efforts to bypass sanctions, including a deal with Iran, were widely covered and added complexity to the geopolitical landscape. At the same time, discussions surrounding the future of sanctions and their effectiveness continued, especially with the Trump administration’s uncertain approach.

The Munich Security Conference was another focal point. Among the key issues were the impact of US foreign policy under President Trump, NATO’s future role, and Russia’s aggression against Ukraine. The conference underscored the global uncertainties caused by shifting US policies, while reaffirming the international community’s commitment to addressing the conflict in eastern Ukraine and Russia’s annexation of Crimea.

Russia’s role in European elections and its influence in global politics garnered significant attention. Reports on cyberattacks, media propaganda, and alleged election interference highlighted the Kremlin’s growing involvement in shaping political outcomes beyond its borders, particularly in France and the US. This continued pattern of interference underscored broader concerns about Russia’s ambitions on the global stage and its impact on democratic processes.

**March**

The Eurovision song contest became a flashpoint in Russia–Ukraine tensions when Ukraine barred Russian participant Yulia Samoilova from entering the country due to her visit to Crimea. This decision drew sharp criticism from Russia and sparked debates about the politicisation of cultural events.

The assassination of former Russian deputy Dmitry Voronenkov in Kyiv captured global attention. Ukrainian authorities attributed the killing to Moscow, framing it as a political assassination linked to Voronenkov’s criticism of the Kremlin and his cooperation with Ukraine’s investigation into Russian aggression. The incident was framed within broader narratives of Russian interference and the dangers faced by Kremlin opponents abroad.

Sanctions against Russia remained a central theme, with protests targeting Russian banks in Ukraine. German Chancellor Angela Merkel’s meeting with Russian President Vladimir Putin underscored the enduring rift over sanctions and Russia’s aggression against Ukraine, while public opinion surveys in Russia suggested that most Russians remained opposed to seeking sanctions relief. The evolving US stance under the Trump administration added uncertainty, as Trump’s initial reluctance to criticise Russia fuelled speculation about potential shifts in policy.

Media coverage brought renewed attention to Russia’s influence abroad, spotlighting Marine Le Pen’s visit to Moscow during the French presidential campaign and allegations of Russian interference in Western politics. These events underscored the Kremlin’s ongoing attempts to expand its reach and undermine democratic processes, prompting sharp criticism from European leaders and strengthening calls for vigilance against disinformation and hybrid threats.

**April**

The death of an OSCE monitor in a mine explosion in eastern Ukraine brought renewed attention to the conflict in eastern Ukraine. The incident underscored the dangers faced by international observers and intensified calls for a ceasefire and the withdrawal of heavy weaponry.

US Secretary of State Rex Tillerson’s visit to Moscow emerged as a key diplomatic moment. Discussions with Russian officials addressed critical issues, including Russia’s aggression against Ukraine, the Syrian crisis, and broader US-Russia relations. Tillerson’s inquiry about the relevance of Ukraine to American interests raised concerns among European allies and policymakers. The comment, interpreted by some as questioning the strategic importance of Ukraine to the US, reflected broader uncertainties surrounding the Trump administration’s foreign policy approach.

Sanctions against Russia remained a focal point. The G7 foreign ministers’ meeting reiterated international pressure on Moscow to comply with the Minsk Agreements and it underscored the G7’s commitment to maintaining sanctions as a critical tool for countering Russian aggression in Ukraine.

Russia’s growing isolation in international fora was further underscored by its withdrawal from the Eurovision song contest following Ukraine’s refusal to allow a Russian participant to enter due to her visit to Crimea. This decision highlighted the ongoing tensions between the two countries and the broader politicisation of cultural events.

**May**

Media coverage of high-profile diplomatic meetings highlighted the ongoing tensions in Russo-Ukrainian relations and Russia’s strained ties with Western powers. German Chancellor Angela Merkel’s visit to Sochi was widely framed as a critical moment in the stagnating peace process in Ukraine. News outlets emphasised Merkel’s firm stance on Russia’s responsibility in the conflict, with particular focus on her condemnation of disinformation campaigns and election interference.

The meeting between Russian Foreign Minister Sergey Lavrov and US President Donald Trump in Washington also received significant attention. Reports centred on the optics of the meeting amidst ongoing investigations into Russian election interference.

The global WannaCry ransomware attack was another major story, with Ukraine singled out as a vulnerable target due to its history of cyber aggression from Russian actors. Commentators in international media pointed to the broader implications of the attack for global cybersecurity, framing it as a consequence of inadequate safeguards and intelligence leaks.

**June**

Media coverage highlighted escalating military confrontations between Russia and Ukraine, notably the conflict in eastern Ukraine and the broader geopolitical implications of these events.

The global cyberattack linked to the Petya ransomware was another major story, particularly in Ukraine. Media outlets framed the attack as a significant threat to international cybersecurity, with Ukraine bearing the brunt of the damage. International coverage focused on the broader implications for global infrastructure, while Ukrainian officials raised concerns over the potential for Russian involvement, with some analysts suggesting that Ukraine was targeted for political disruption rather than financial gain.

The EU extended sanctions on Russia over its annexation of Crimea. Media outlets across Europe highlighted the ongoing diplomatic tensions, with particular emphasis on the EU’s efforts to maintain pressure on Russia, despite some opposition to the Nord Stream 2 pipeline deal, which divided European opinion.

**July**

The passage of a new US sanctions package targeting Russia dominated headlines, with reports highlighting the bipartisan consensus in Congress as a sign of growing unease over Russia’s geopolitical actions, including election interference and aggression in Ukraine.

The G20 summit in Hamburg provided a platform for discussions on eastern Ukraine and international relations. The first face-to-face meeting between US President Donald Trump and Russian President Vladimir Putin was heavily scrutinised, with commentators questioning Trump’s stance on Russian election interference and his commitment to Ukrainian sovereignty. Reports noted limited progress in resolving the Ukraine conflict.

Cyberattacks attributed to Russian-linked actors continued to make waves. Analysts linked recent attacks on Ukraine’s power grid to broader concerns about Russian cyber capabilities, with media framing these incidents as part of hybrid warfare tactics.

**August**

Ukraine intensified efforts to address the conflict in eastern Ukraine, passing a reintegration bill that formally designated Russia as an aggressor state. Media coverage highlighted Ukrainian attempts to counter Russian propaganda in the region by jamming Russian television channels and exposing alleged enrichment schemes by separatist leaders. Discussions also centred on revising the Minsk Agreements to develop a new road map for conflict resolution.

The question of military aid to Ukraine gained prominence, with reports on US deliberations over supplying lethal weapons to counter Russian aggression. This coincided with allegations of Ukraine’s involvement in supplying missile engines to North Korea, claims that Kyiv vehemently denied, citing strict export controls and international compliance.

Sanctions against Russia remained a focal point as the US enacted a new sanctions package targeting Moscow, despite reservations from President Trump. European leaders, including Germany’s Angela Merkel, reiterated that lifting sanctions depended on progress in resolving the conflict in eastern Ukraine. Reports also scrutinised gaps in enforcement, noting Western companies’ roles in aiding Russian oil projects despite restrictions.

Construction of the Kerch Strait Bridge linking Russia to Crimea continued to spark controversy. Ukrainian officials criticised the project for restricting navigation in the Black Sea and undermining Ukraine’s sovereignty.

**September**

The possibility of a UN peacekeeping mission in Donbas attracted significant international attention. The UN General Assembly held meetings on the conflict in eastern Ukraine, with discussions involving potential peacekeeping efforts, but no consensus emerged.

Sanctions against Russia were extended as the EU launched an interactive sanctions map aimed at improving transparency for businesses and officials. This came amid continued debates on the effectiveness of sanctions and Russia’s responses.

Cyber threats linked to Russian actors remained a significant issue, with the EU conducting a cyber war game to test responses to simulated hacker attacks on military missions. International reports highlighted Russian interference in Europe and North America, with cyberattacks and disinformation campaigns recognised as part of a wider hybrid warfare strategy.

US-Russia relations were marked by continued tensions, with the US strongly condemning Russia’s imposition of citizenship on Ukrainian citizens in Crimea and its human rights abuses in the region. These actions, along with reports of arbitrary arrests and torture of Ukrainians in Crimea, further escalated the conflict between the two countries.

**October**

Sanctions against Russia remained a central issue, with increased scrutiny on how Russian companies were bypassing these restrictions. Media outlets reported that Russia was using intermediaries, such as third-party countries and shell companies, to circumvent sanctions, a move that was framed as a challenge to international efforts to limit Russia’s economic reach. These revelations sparked discussions on the effectiveness of sanctions and the need for tighter enforcement.

NATO and Russia’s relationship grew more strained. NATO responded to increasing Russian military activity and perceived threats by establishing a counter-espionage hub in Poland and a joint centre in Finland aimed at countering hybrid warfare. NATO’s actions were seen as an effort to counter the growing threat of Russian expansionism and to reassure Member States in the region.

Cybersecurity emerged as a significant concern, with the Bad Rabbit ransomware attack gaining international attention. Linked to Russian cyber actors, the attack targeted Ukraine and other countries, disrupting systems and drawing attention to the growing threats of cyber warfare. Media outlets framed the attack as part of Russia’s hybrid warfare strategy to destabilise Western countries.

The issue of a UN peacekeeping mission in Donbas continued to garner attention, although without significant breakthroughs. Media coverage highlighted diplomatic efforts to resolve the conflict in eastern Ukraine, including Ukraine’s call for a UN peacekeeping mission to help stabilise the region. The lack of consensus on the mission underscored the complex geopolitical dynamics at play.

**November**

The Asia-Pacific Economic Cooperation (APEC) summit drew attention for its Putin-Trump meeting, where discussions reportedly touched on US-Russia relations, the conflict in Ukraine, and cyber interference.

The Eastern Partnership summit became a focal point for examining EU relations with Ukraine and other former Soviet republics. Media outlets highlighted Ukraine’s aspirations for closer EU integration amidst discussions on resistance to Russian influence in the region. Reports noted the challenges of balancing Ukraine’s European ambitions with its ongoing war and internal reforms.

In the conflict in eastern Ukraine, prisoner exchange negotiations made headlines, with Ukrainian and Russian representatives engaging in talks that underscored the humanitarian toll of the conflict. Meanwhile, efforts to introduce UN peacekeepers in the region remained stalled, with international media portraying the impasse as emblematic of broader geopolitical tensions between the West and Russia.

Human rights abuses in Crimea under Russian occupation were widely reported, with issues such as political repression, arbitrary arrests, and deteriorating living conditions in the spotlight.

**December**

Media coverage focused on the withdrawal of Russian officers from the Joint Centre for Control and Coordination (JCCC), a mechanism established to oversee ceasefires in Donbas. This development was seen as a blow to international monitoring mechanisms, and Ukrainian officials criticised the move as a deliberate attempt to escalate tensions in Donbas.

The issue of arms supply to Ukraine also dominated headlines, with the US approving the sale of lethal weapons to support Ukraine’s defence against Russian aggression.

Humanitarian narratives focused on prisoner exchanges between Ukraine and separatist forces. This significant development, facilitated by religious leaders from the Ukrainian Orthodox Church and the Moscow Patriarchate, was widely reported as a rare moment of cooperation amidst the protracted conflict. Media coverage highlighted the emotional reunions of released captives and the role of faith-based diplomacy in fostering dialogue.

Sanctions against Russia remained a recurring theme, with the EU formally extending economic measures for six months in response to Moscow’s actions in Ukraine. Media outlets noted that the decision reflected ongoing European unity on the issue, despite diverging opinions among Member States regarding relations with Russia.

**2018**

**January**

Media highlighted the sanctions against Russia, examining their impact on Russia’s economy. While supporters argued that sanctions pressured Moscow over Ukraine, critics questioned their effectiveness, noting Russia’s continued defiance. Diverging views within the EU, particularly from states reliant on Russian energy, underscored challenges in maintaining a united Western stance.

The gas dispute between Russia and Ukraine resurfaced, alongside controversies over Nord Stream 2, with the US and Poland opposing the project over European energy security concerns and its geopolitical ramifications.

Russian Foreign Minister Sergei Lavrov reignited debates on Ukraine’s territorial integrity. Lavrov criticised Western nations for alleged double standards, framing the annexation of Crimea as consistent with the region’s right to self-determination. These statements drew significant international attention.

Media also reported on a proposal from factions within the US government to reduce the military presence in Eastern Europe as a potential concession to Russia, aimed at easing tensions and advancing arms control discussions. This raised concerns among NATO allies, particularly in Eastern Europe, about the security implications for the alliance.

**February**

Media coverage highlighted continued diplomatic efforts focused on potential peacekeeping missions and prisoner exchanges. Yet tensions remained high along the border, particularly in Donbas, where heavy fighting persisted despite ceasefire agreements. The humanitarian situation in these areas grew more dire, with mounting calls for international intervention to prevent further escalation.

The Nord Stream 2 gas pipeline project continued to dominate discussions on European energy security, with Russia facing strong opposition from the US, Poland and Ukraine. Critics argued that the pipeline would increase Europe’s dependency on Russian gas, further strengthening Russia’s geopolitical influence. Meanwhile, some EU countries, including Germany, pushed for its completion, seeing it as a crucial element in securing their own energy needs.

Ukraine’s military salute reform attracted attention, as the country moved to replace the old Soviet-era salute with a more nationalistic expression, “Слава Україні!” (“Glory to Ukraine!”). This shift was seen as part of a broader effort to solidify Ukraine’s national identity, distancing itself from Russia and emphasising its sovereignty amid the ongoing war.

The Munich Security Conference highlighted global tensions, with the conflict in eastern Ukraine prominently featured in discussions on European security and Russia’s role in destabilising the region. As Western leaders continued to grapple with the challenge of addressing Russia’s aggressive actions, the conference underscored the growing concerns about the broader implications of Russia’s aggression against Ukraine for international relations.

**March**

The Skripal poisoning case, which involved the poisoning of former Russian spy Sergei Skripal and his daughter Yulia in Salisbury, UK, dominated headlines. The UK government accused Russia of being behind the nerve agent attack. The EU, alongside several Western nations, responded by expelling Russian diplomats in solidarity with the UK.

The Russian presidential elections reasserted Vladimir Putin’s dominance in Russian politics. However, the elections were marked by accusations of electoral manipulation and restricted access for international observers, particularly in Crimea, where Ukrainian citizens faced significant limitations. The elections sparked a range of reactions globally, with criticism from the West over the absence of genuine competition and accusations of interference.

The gas dispute between Russia and Ukraine, exacerbated by the Nord Stream 2 pipeline project, remained a key issue. While the pipeline project promised to reduce Europe’s reliance on Ukrainian gas transit, it was met with fierce opposition from Ukraine, the US and some EU countries, who warned of the geopolitical risks and energy dependence it would impose.

In US-Russia relations, a significant moment occurred with a phone call between US President Donald Trump and Vladimir Putin. The call, which followed Putin’s electoral victory, raised concerns in the West about Trump’s conciliatory stance toward the Russian leader. The lack of clear pushback on issues such as Russia’s actions in Ukraine, the Skripal poisoning and allegations of election interference raised further questions about the direction of US foreign policy under Trump and its impact on transatlantic relations.

**April**

The aftermath of the Skripal poisoning continued to dominate international headlines. As Sergei and Yulia Skripal began their recovery in a UK hospital, analysis of the attack intensified, with Western governments maintaining their accusation of Russian involvement. This led to coordinated diplomatic expulsions, with over 150 Russian diplomats expelled globally in solidarity with the UK. Russia’s retaliatory measures further deepened the diplomatic rift.

Sanctions against Russia remained a focal point of global discussions, particularly during the G7 meeting. Western leaders debated the effectiveness and potential escalation of economic measures to pressure Russia over its actions in Ukraine and the Skripal case. These deliberations underscored the challenges of maintaining a unified stance within the international community, as some nations expressed concerns over the broader economic impact of sanctions.

The seizure of a Ukrainian fishing vessel by Russian forces in the Sea of Azov also captured media attention. Meanwhile, the inauguration of the Crimean Bridge connecting mainland Russia to Crimea was depicted as a symbolic assertion of Russian control over the annexed territory, further inflaming Ukrainian protests and international condemnation.

In Ukraine, the Orthodox Church’s push for autocephaly — independence from the Russian Orthodox Church — emerged as a significant narrative. The move was framed as a step toward consolidating national identity and resisting Russian influence.

**May**

The staged assassination of Russian journalist Arkady Babchenko in Ukraine shocked international audiences. Initially reported as a murder, it was later revealed that Ukrainian authorities had orchestrated the event to foil an alleged assassination plot against the journalist. This controversial tactic sparked debates over its ethical implications and potential damage to Ukraine’s credibility, with critics questioning the broader impact on journalism and trust in the media.

The investigation into the MH17 tragedy advanced significantly, with the Netherlands and Australia formally accusing Russia of responsibility. Evidence linked the missile system used in the downing of the flight to the Russian military. Moscow denied the accusations, framing them as politically motivated, while the international community intensified demands for accountability.

The inauguration of Vladimir Putin’s fourth presidential term underscored the challenges of his leadership, both domestically and internationally. Protests erupted across Russia ahead of the ceremony, resulting in several arrests. Internationally, Western leaders scrutinised Putin’s role in Russia’s aggression against Ukraine, the MH17 case, and strained global relations, marking a tense start to his new term.

The construction of the Kerch Strait Bridge connecting Russia to Crimea was another significant development. While Russia celebrated the bridge as a symbol of unity and engineering prowess, Ukraine and its allies condemned it as a violation of Ukraine’s sovereignty and territorial integrity.

**June**

The G7 summit brought to the forefront divisions among Western leaders, particularly regarding Russia’s exclusion from the group. While some voiced support for Russia’s potential return to the G8, citing the need for dialogue and cooperation, others strongly opposed this notion, highlighting Russia’s ongoing aggression in Ukraine as a key reason for its continued exclusion. Media coverage emphasised the rift between G7 members, with debates about the future of Russia’s relationship with the West and the implications of its continued isolation.

The upcoming Trump-Putin summit, scheduled for mid-July, sparked intense media debate, with many questioning whether it would result in any shift in US policy on Russia, particularly regarding the conflict in Ukraine.

Prisoner exchange negotiations between Russia and Ukraine garnered significant media attention, particularly the case of Ukrainian political prisoner Alexander Kolchenko. After being arrested and sentenced to 10 years in a Russian penal colony, Kolchenko was forced to accept Russian citizenship under duress, a move that highlighted Russia’s tactics in exerting pressure on detained Ukrainians. Media reports focused on the human rights violations surrounding Kolchenko’s case, symbolising the broader concerns tied to the conflict and prisoner exchanges between the two nations.

The Nord Stream 2 gas pipeline remained a point of contention, with European leaders, including Polish President Duda, vocally opposing the project. This dispute underscored concerns over Ukraine losing crucial gas transit fees and political leverage, further complicating the already fraught energy relations between Russia and Ukraine.

**July**

The Trump-Putin summit, held on 16 July in Helsinki, drew global attention for its controversial discussions, including a proposal by Russian President Vladimir Putin to hold a referendum in occupied territories of eastern Ukraine on their status. The idea was met with widespread condemnation from Ukraine and its allies, who viewed it as an attempt to legitimise Russia’s control over these regions under the guise of local self-determination. The fallout from the summit heightened concerns about the potential erosion of international support for Ukraine’s sovereignty, with media narratives focusing on the geopolitical risks posed by the US-Russia dialogue.

Sanctions against Russia remained a key focus, particularly the EU’s decision to target companies involved in constructing the Crimean Bridge. Media coverage highlighted the sanctions’ significance in reinforcing the EU’s stance against Russia’s actions in Crimea, while emphasising the broader economic and political implications of the ongoing measures.

The humanitarian toll of Russia’s war against Ukraine was exemplified by the plight of Ukrainian political prisoners held in Russia, particularly filmmaker Oleg Sentsov, who went on a hunger strike to demand the release of fellow Ukrainian prisoners. The move captured international attention and drew sharp criticism of Russia’s human rights practices. Despite mounting pressure for a prisoner exchange, negotiations showed little progress. Media reports underlined the symbolic importance of Sentsov’s stance as part of Ukraine’s broader effort to galvanise global support in its resistance against Russia.

**August**

The Merkel-Putin summit, held on 18 August, drew significant media attention, particularly regarding discussions on the conflict in eastern Ukraine. The summit highlighted the lack of progress in peace negotiations, with both leaders facing pressure over their handling of the crisis. The media’s focus was on Russia’s continuing influence in the region and the limited results of the Normandy Format talks.

The issue of sanctions against Russia remained prominent. While the EU continued to extend its sanctions, the Trump administration signalled openness to lifting them in exchange for Russian cooperation on Syria and Ukraine, raising concerns that this could weaken international resolve. Media coverage framed this divergence as a potential shift in the West’s unified stance against Russia.

The case of Ukrainian filmmaker Oleg Sentsov, who continued his hunger strike in a Russian prison, kept capturing global attention. Reports focused on his deteriorating health and growing international calls for his release, with some speculating about a possible prisoner exchange. Media coverage also emphasised the broader issue of political prisoners in Russia and the ongoing negotiations for their exchange.

The Nord Stream 2 gas pipeline project remained a contentious issue, with the media focusing on Ukraine’s opposition. Ukraine feared that the pipeline, bypassing its gas transit system, would significantly undermine its strategic position in Europe and further diminish its leverage over Russia. Reports highlighted the geopolitical implications, with concerns about increased European dependence on Russian energy.

**September**

Confrontations between Russia and Ukraine escalated, particularly in the Sea of Azov. The conflict intensified as Russia’s military presence in the region grew, drawing international concern over the stability of the area, vital for both countries’ access to critical ports. Media coverage focused on Ukraine’s efforts to safeguard its maritime routes and the increasing difficulties in de-escalating the situation.

A significant development was Ukraine’s formal termination of the Treaty on Friendship, Cooperation, and Partnership with Russia, a move aimed at demonstrating Ukraine’s commitment to distancing itself from Russian influence. The decision came amidst growing frustration over Russia’s actions in Crimea and eastern Ukraine, reinforcing Ukraine’s pivot toward the West. The media spotlighted the symbolic and practical implications of the treaty’s termination, framing it as a decisive step in Ukraine’s struggle to assert its sovereignty.

The assassination of Donetsk People’s Republic (DPR) leader Alexander Zakharchenko further complicated the conflict in eastern Ukraine. His death, amid ongoing fighting in the region, prompted various international reactions, with media coverage focusing on the impact of his assassination on peace prospects.

The issue of sanctions against Russia remained high on the international agenda. The EU extended sanctions and continued to emphasise its support for Ukraine’s territorial integrity, with German Chancellor Angela Merkel playing a key role in reinforcing the EU’s position. This was juxtaposed with growing divisions within the international community, as the Trump administration’s willingness to lift sanctions in exchange for Russian cooperation on Syria and Ukraine raised concerns about the weakening of the Western bloc’s stance.

**October**

Tensions between the Russian Orthodox Church and the Ecumenical Patriarchate escalated over the issue of Ukrainian autocephaly. The decision of the Patriarchate of Constantinople to grant independence to the Ukrainian Orthodox Church sparked a major schism, with Moscow severing ties in protest. Media coverage highlighted the religious and political implications of this move, framing it as a symbolic break from Russia’s historical influence over Ukraine and a significant step in Ukraine’s assertion of its sovereignty.

The Kerch Polytechnic College attack in Crimea shocked the region and drew widespread international attention. An explosion and mass shooting left 20 people dead and dozens injured, with the Russian authorities classifying the event as a terrorist attack before later attributing it to a lone gunman. Coverage focused on the tragedy’s impact on the local community, while some reports underscored the challenges faced in the region under Russian occupation.

Sanctions against Russia remained a focal point in international diplomacy. German Chancellor Angela Merkel’s visit to Ukraine underscored the EU’s commitment to supporting Ukraine, with discussions centring on the conflict in eastern Ukraine and the continuation of sanctions.

The Nord Stream 2 gas pipeline project remained contentious, with Ukraine pursuing a legal challenge to mitigate its potential economic and strategic losses. Media reports framed the project as deepening European energy dependence on Russia, while Ukraine sought alternative measures to secure its energy transit revenues and geopolitical leverage.

**November**

Confrontations between Russia and Ukraine reached a new high with the Kerch Strait naval incident. Russian forces seized three Ukrainian vessels and detained their crews, accusing them of illegal entry into Russian waters. Ukraine decried this act as a violation of international law, prompting widespread international condemnation. Media coverage underscored the incident’s implications for maritime rights, regional security, and the already hostile Russia–Ukraine relations.

In response to escalating tensions, Ukraine imposed martial law in select regions, a move framed as a defensive measure against potential Russian aggression. Reports highlighted debates surrounding the political and security implications of martial law, with former Ukrainian presidents expressing differing views on its timing and potential impact on upcoming elections.

Sanctions remained a key theme, with the Kremlin announcing sanctions targeting prominent Ukrainian figures, including former Prime Minister Yulia Tymoshenko and former Interior Minister Arsen Avakov, as well as companies. Simultaneously, the EU and other Western nations explored further measures to address Russia’s aggression.

The MH17 tragedy re-emerged as families of victims filed a lawsuit against Russia at the European Court of Human Rights. Media coverage focused on the pursuit of accountability and the broader implications for justice in the face of Russian denial and disinformation campaigns.

Religious tensions persisted, with attacks on churches in Kyiv linked to the ongoing Orthodox Church schism. Reports framed these incidents within the larger context of Ukraine’s efforts to assert independence from Russian influence, both politically and spiritually.

The G20 summit spotlighted global responses to Russia’s aggression against Ukraine. US President Donald Trump cancelled a planned meeting with Vladimir Putin, citing the Kerch Strait incident as a key reason. This diplomatic snub underscored the growing international unease over Russia’s actions, as the media framed the incident as a test of the West’s resolve in confronting Russian aggression.

**December**

The conflict between Russia and Ukraine were marked by the fallout from the Kerch Strait incident, where Russian forces detained Ukrainian sailors, leading to calls for international pressure on Russia to release the detainees, respect international maritime law, and de-escalate tensions in the Black Sea region. Religious tensions also escalated with the formal establishment of Ukraine’s independent Orthodox Church. Media coverage highlighted the struggles within Ukraine’s religious community, with reports focusing on the pressure placed on clergy and accusations of collaboration with pro-Russian forces.

The Nord Stream 2 pipeline remained a highly contentious issue, with continued concerns over its geopolitical and economic implications. Discussions focused on the increasing opposition from Ukraine and other European nations, who warned about the risks of further energy dependency on Russia. The EU and the US continued to voice their opposition, with Washington pushing for sanctions on entities involved in the project, aiming to disrupt its progress.

**2019**

**January**

The Kerch Strait incident remained a focal point, with renewed debate over potential military responses, sanctions, and diplomatic measures. International actors weighed in, while media attention centred on Russia’s extension of the detention of Ukrainian sailors and the possibility of a prisoner exchange.

Coverage also focused on the autocephaly of the Ukrainian Orthodox Church. Reports addressed the relocation of church institutions, the transfer of religious property and broader geopolitical implications.

The upcoming Ukrainian presidential and parliamentary elections also featured prominently, with concerns over potential Russian interference and the role of OSCE observers. Media narratives scrutinised political candidates, including Poroshenko and Volodymyr Zelenskyy, with particular attention to allegations regarding Zelenskyy’s business ties to Russia.

**February**

Media coverage of Ukraine’s upcoming presidential and parliamentary elections remained prominent, with particular focus on Kyiv’s decision to ban Russian observers from monitoring the vote.

Reporting on the broader Russia–Ukraine conflict continued, highlighting military tensions over Crimea, the Minsk Agreements, sanctions against Russia, and disputes over gas and coal exports. The visit of Kurt Volker, the US Special Representative for Ukraine Negotiations, also attracted attention, as he remained active in diplomatic efforts to address the conflict.

The Nord Stream 2 pipeline featured in discussions on EU-Russia relations, with coverage exploring Germany’s role, European energy security, and regulatory debates over dependence on Russian gas. The Kerch Strait incident remained in the spotlight, with reports focusing on the detention of Ukrainian sailors, potential sanctions against Russia, and legal proceedings at the European Court of Human Rights.

**March**

The conflict between Russia and Ukraine remained a central focus, with particular attention on NATO’s response to Russian actions and broader European security concerns. Media coverage highlighted the defection of Vasyl Prozorov, a former officer of Ukraine’s Security Service (SBU), whose claims about the Ukrainian government and its role in the eastern Ukraine conflict stirred controversy. Meanwhile, Putin’s visit to Crimea, marking five years since its annexation from Ukraine, attracted widespread coverage.

Concerns over external interference in Ukraine’s presidential election were also widely discussed, with reports detailing cyberattacks aimed at disrupting the electoral process or undermining public confidence. The role of Russian media and journalists in the election was another point of contention, with Ukraine restricting access to Russian outlets and reporters.

The annexation of Crimea remained a key issue, particularly in relation to human rights violations against Crimean Tatars under Russian rule. Reports highlighted arbitrary arrests, disappearances, and harassment of activists and community leaders, drawing international criticism of Russia’s administration of the peninsula.

**April**

Ukraine’s presidential and parliamentary elections dominated media coverage, with Volodymyr Zelenskyy’s landslide victory over Petro Poroshenko marking a major political shift. Reporting focused on Zelenskyy’s approach to Russia–Ukraine relations, particularly his expressed willingness to engage in dialogue with Russian President Vladimir Putin to resolve the conflict in eastern Ukraine.

The conflict in eastern Ukraine remained a central issue, with significant attention on Putin’s decree introducing a simplified procedure for residents in the region to obtain Russian citizenship.

Ukraine’s parliament passed the so-called “language law,” reinforcing the use of Ukrainian in public life. The law sparked debate over its impact, particularly on the country’s Russian-speaking population.

Confrontations in the Black Sea were also widely discussed, with NATO’s increased presence framed as both a reassurance to Eastern European allies and a deterrent against Russian aggression. Ukraine’s continued military reforms, increased defence spending, and modernisation efforts were covered, highlighting NATO’s role in supporting Kyiv’s security and defence capabilities.

**May**

Russia–Ukraine confrontations remained a prominent issue, with significant focus on newly elected President Volodymyr Zelenskyy. Viewed as a potential catalyst for change, Zelenskyy’s presidency brought a shift in Ukraine’s approach to Russia. He emphasised the importance of achieving peace in eastern Ukraine and signalled a readiness to engage in dialogue with Russia. Coverage centred on the challenge of negotiating a resolution to the conflict in eastern Ukraine, focusing on the terms of potential talks and the involvement of international partners to ensure a fair and balanced approach.

Discussions also highlighted economic issues, particularly those related to the transit of Russian oil and gas. The contamination of oil from the Druzhba pipeline and the continued transit of Russian gas through Ukraine were key topics, with rising fuel prices in Europe framed as a direct consequence.

The conflict in eastern Ukraine remained a point of concern, with frequent ceasefire violations from both Ukrainian and Russian-backed forces. Accusations were exchanged between both sides, further complicating the path to peace.

The Nord Stream 2 pipeline continued to feature prominently, with ongoing debates about its geopolitical implications for energy security, economic interests, and regional politics. Its construction sparked concern in Europe, particularly regarding Russia’s influence and the potential impact on transatlantic relations.

**June**

Developments in the investigation into the MH17 crash renewed significant media interest. Malaysian Prime Minister Mahathir Mohamad’s comments questioning the impartiality of the investigation were widely reported. Mohamad expressed scepticism regarding the findings that implicated Russia, suggesting that the investigation might have been politically motivated. The Joint Investigation Team (JIT) charged four individuals — three Russians and one Ukrainian — with murder in connection to the downing of MH17.

The G20 summit in Osaka also drew considerable attention, with the event serving as a backdrop for indirect discussions related to Russia’s aggression against Ukraine. Of particular interest was the meeting between US President Donald Trump and Russian President Vladimir Putin, which attracted significant media coverage. This marked the first meeting between the two leaders following the conclusion of Special Counsel Robert Mueller’s investigation into Russian interference in the 2016 US presidential election.

Ukrainian President Zelenskyy was actively engaged with international leaders and institutions, seeking support for Ukraine and pushing for peace in the region. The EU remained involved in discussions about the Minsk Agreements, which aimed to establish a ceasefire and political resolution in eastern Ukraine.

**July**

The conflict in eastern Ukraine continued to make headlines, with media reports detailing fresh clashes and escalating hostilities. Coverage underscored the humanitarian consequences of the conflict, particularly concerning civilian casualties and displacement.

Russian disinformation targeting Ukraine was a recurring theme, with outlets highlighting Kremlin-backed narratives that sought to portray Ukraine as the aggressor. Coverage pointed to claims circulated by Russian state media and affiliated platforms alleging that Ukrainian companies were financing terrorism and illicit activities in occupied Donbas. Analysts and independent media sources countered these accusations, framing them as part of a broader disinformation strategy aimed at undermining Ukraine’s international reputation.

Ukraine’s political landscape was in the spotlight following Volodymyr Zelenskyy’s election victory, with the media analysing the implications of the presidential and parliamentary elections for domestic and foreign policy.

Vladimir Putin’s visit to Italy received significant attention, with coverage examining Russia-Italy relations in the context of EU sanctions and diplomatic manoeuvring. Germany-Russia relations were also widely discussed, particularly regarding the impact of sanctions on Germany and ongoing diplomatic engagement between Berlin and Moscow.

**August**

The conflict in eastern Ukraine remained at the forefront of media coverage, as continued violence and military engagements exacerbated the humanitarian crisis in the region. Reports detailed ceasefire violations and examined the broader implications of ongoing hostilities, particularly in relation to diplomatic efforts.

The MH17 investigation resurfaced in media discussions, with some reports drawing connections to the downing of a Ukrainian Boeing in Donbas.

Russian disinformation targeting Ukraine remained a prominent theme. Reports highlighted Kremlin-backed narratives that sought to portray Ukraine as the aggressor, with Russian state media amplifying claims of Ukrainian military crimes in Donbas. Other narratives included allegations of Ukrainian cultural institutions funding projects that trivialised the war, accusations of child recruitment into military-patriotic clubs, and claims that Russia’s Investigative Committee was uncovering new evidence of Ukrainian attacks in Donbas. Independent analysts and Western media outlets countered these narratives, framing them as part of a broader disinformation campaign aimed at shaping international perceptions of the conflict.

**September**

One major point of focus was the ongoing discussions around a potential Russia–Ukraine prisoner exchange, which generated significant attention as both sides worked toward agreements.

The media also focused on growing concerns over the stability of Naftogaz, Ukraine’s state-owned oil and gas company, particularly in relation to the termination of Russian gas transit and the approaching winter season. Coverage examined the risks to Ukraine’s energy security and the broader economic implications of potential disruptions.

**October**

Developments in Russia’s war against Ukraine continued to dominate coverage, with fresh reports on military engagements and ongoing geopolitical tensions.

The US-Ukraine impeachment inquiry — focused on President Donald Trump’s dealings with Ukraine, particularly allegations that he pressured Ukraine to investigate his political rival — gained momentum, as new details emerged linking Ukrainian officials to key events in the investigation.

Ukraine’s legal and economic efforts were a focal point, particularly regarding the establishment of an independent gas transmission system operator and amendments to transit contracts with Russia. These moves were seen as crucial steps in securing Ukraine’s energy independence and reducing reliance on Russian gas transit.

Russian narratives aimed at portraying Ukraine as a criminal state persisted in October, with Russian authorities launching investigations into alleged criminal activities in Donbas. These narratives were amplified by state-controlled media and were part of a broader disinformation campaign to discredit Ukraine on the international stage.

**November**

The Russia–Ukraine confrontation remained a focal point in media coverage, with reports highlighting new diplomatic tensions and geopolitical manoeuvres.

The Normandy Format summit, aimed at resolving the conflict in eastern Ukraine, gained significant attention as world leaders engaged in negotiations focused on de-escalation and potential pathways to peace.

The MH17 investigation made further progress, with new evidence emerging that linked Russian officials to separatists in Ukraine. This development intensified international calls for accountability, with many urging Russia to take responsibility for its role in the tragedy.

**December**

Gas transit agreements between Russia and Ukraine dominated discussions, with negotiations leading to significant developments that impacted European energy security.

Despite the ongoing conflict in eastern Ukraine, peace talks remained a priority, with diplomatic efforts continuing despite frequent setbacks.

The prisoner exchange process garnered attention, with the media focusing on its humanitarian and diplomatic significance for both nations.

The Trump impeachment inquiry continued to feature Ukraine-related developments, as new revelations further linked the country to US political controversies.

**2020**

**January**

One of the key stories was Ukraine’s participation in the Council of Europe Parliamentary Assembly, where its delegation sat alongside representatives from Russia. This move sparked considerable controversy, as critics pointed to ongoing tensions over Russia’s actions in Ukraine and its role in the international community, raising questions about Ukraine’s stance in engaging with Russia amid continued hostilities.

The MH17 investigation continued to progress, with new evidence shedding light on the role of Russian-backed separatists in the downing of the flight. The investigation remained a focal point of international attention, with legal proceedings unfolding in various courts as countries and victims’ families pressed for accountability. The media analysed how this case was shaping global views of Russia’s involvement in the conflict.

Political and security concerns in Ukraine escalated as discussions emerged surrounding Russian extradition demands. These demands were linked to several high-profile cases involving Ukrainian nationals in Russia and abroad.

**February**

Media coverage highlighted the sentencing of Ukrainian political figures in Russian courts, raising concerns about Russia’s judicial practices and the political implications for those caught in the legal system.

Reports emerged on the issue of forced “passportisation” in the occupied territories of eastern Ukraine, with Russia continuing its policy of distributing Russian passports to residents.

The MH17 crash investigation remained a central point of international discourse, with new developments continuing to shed light on the role of Russian-backed separatists in the tragedy.

As discussions around energy security and the role of Russian influence in Europe gained momentum, the geopolitical implications of the Nord Stream 2 gas pipeline came under closer scrutiny. Coverage explored the potential consequences of the pipeline on Ukraine’s energy security and its broader effects on European relations, particularly with Germany and the EU.

Finally, a notable media narrative that emerged in February centred on the portrayal of Russia and President Putin as victims in the US impeachment trial. Coverage suggested that Russian officials, particularly Putin, were being unjustly vilified within the broader political discourse surrounding the trial. Russian media outlets capitalised on this narrative, emphasising the notion that Russia was unfairly targeted by international criticism, especially in relation to its actions in Ukraine and its perceived influence on US domestic politics. This portrayal served to deflect attention from Russia’s role in these contentious issues, positioning the country as a victim of geopolitical and ideological attacks rather than as an active participant in the conflicts.

**March**

As the MH17 trial advanced, international attention remained fixed on Russia’s involvement in the tragedy, with media outlets scrutinising the role of Russian-backed separatists and the broader implications for international law and accountability.

Ukraine’s stance on the Donbas settlement, particularly regarding the Minsk Agreements, was a point of contention. Reports emphasised Ukraine’s disagreements with Russia over the terms of the accords and the challenges of reaching a resolution to the conflict. Media outlets continued to examine the complexities of the conflict and the diplomatic impasse.

NATO-Ukraine relations remained in the spotlight, with coverage detailing both the opportunities and tensions arising from Ukraine’s increasing ties with the Western alliance. Russian countermeasures to NATO’s expansion and Ukraine’s aspirations for greater integration with the West were also widely reported.

A significant media cluster centred on President Volodymyr Zelenskyy**,** drawing attention to the various challenges his administration was grappling with, particularly in the areas of the economy, corruption, and the influence of oligarchs. This narrative reflected both domestic frustrations and external criticisms of Ukraine’s governance under Zelenskyy’s leadership, with many commentators questioning his ability to enact meaningful reform.

**April**

In April, the conflict in eastern Ukraine remained a focal point, with reports continuing to highlight ceasefire violations and armed clashes.

Meanwhile, Ukraine continued to grapple with ongoing political challenges, including mounting concerns over corruption and the influence of oligarchs, which persisted as significant issues for the country’s governance.

The COVID-19 pandemic added a new layer of complexity, as Ukraine accused Russia of engaging in disinformation campaigns aimed at exploiting the crisis. These efforts were seen as an attempt to destabilise Ukraine’s domestic situation.

The Normandy Format negotiations showed limited progress. Diplomatic engagements continued, but there were few signs of tangible advancement toward peace.

**May**

Diplomatic efforts over the conflict in eastern Ukraine remained at the forefront, with new discussions taking place within the Tripartite Contact Group, a diplomatic body established to facilitate dialogue between Ukraine, Russia, and the Organisation for Security and Cooperation in Europe (OSCE). These negotiations aimed to de-escalate tensions and find a long-term resolution, though little tangible progress was made in the face of continuing hostilities.

The MH17 investigation generated international attention, with new legal actions and evidence contributing to a broader debate about Russia’s involvement in the downing of the flight.

Confrontations between Russia and Ukraine continued to intensify, with disputes over NATO military exercises in the Black Sea. Ukraine saw these exercises as a vital display of international support, while Russia viewed them as a provocative show of force near its borders.

The gas transit negotiations remained a point of contention, with discussions focused on Ukraine’s role as a transit country for Russian gas to Europe and the implications for Ukraine’s energy security.

**June**

The conflict in eastern Ukraine remained a focal point, with ongoing hostilities between Ukrainian and Russian-backed forces. Despite efforts for a peaceful resolution, the conflict continued to cause significant casualties and instability in eastern Ukraine, with the international community urging both sides to respect ceasefire agreements.

The EU extended its economic sanctions against Russia, maintaining pressure on Moscow in response to its actions in Ukraine. The sanctions, which targeted key sectors of the Russian economy, underscored the EU’s continued stance on holding Russia accountable for its role in the crisis.

Russian disinformation campaigns remained a central issue, with Ukraine actively working to expose and disrupt efforts aimed at destabilising the country. These campaigns targeted both domestic and international audiences, seeking to undermine Ukraine’s sovereignty and portray the government in a negative light.

The ongoing debates around energy security were dominated by the Nord Stream 2 gas pipeline, which became a significant geopolitical issue. As Ukraine sought to secure its energy independence, the pipeline’s potential to bypass Ukrainian transit routes raised concerns about Russia’s growing influence in Europe’s energy market.

Politically, Ukraine faced internal challenges, with widespread protests and increasing calls for the resignation of Minister of Internal Affairs Arsen Avakov. The country’s political instability was further exacerbated by criminal investigations into former President Petro Poroshenko, which highlighted ongoing concerns over corruption and governance in the country.

**July**

The conflict in eastern Ukraine remained a significant issue, with ongoing clashes between Ukrainian and Russian-backed forces. Despite calls for peace, the situation continued to deteriorate, causing further casualties and deepening the humanitarian crisis in eastern Ukraine.

Leaked audio recordings brought Ukraine’s stance on Russia into the spotlight. The recordings allegedly captured conversations between Ukrainian officials and foreign leaders discussing sensitive matters, including the handling of the conflict in eastern Ukraine and Ukraine’s foreign policy. In particular, the tapes seemed to reveal discussions on the potential for negotiating with Russia on certain terms, which critics interpreted as a sign of Ukraine’s willingness to make concessions to Moscow. The leak sparked political controversy within Ukraine, as it appeared to suggest a shift in the government’s stance on Russia and raised suspicions about the possible influence of foreign actors on domestic policy.

NATO’s monitoring of tensions in the Black Sea remained a key theme. Ukraine, alongside NATO allies, expressed concerns over Russia’s growing military presence in the region.

Energy independence from Russia continued to be a topic of debate within Ukraine. Discussions focused on how to reduce Ukraine’s reliance on Russian gas, with challenges related to gas transit routes and the future of energy security in the region. The threat posed by the Nord Stream 2 pipeline continued to be a point of contention in both domestic and international discussions.

**August**

Discussions emerged about Ukraine’s ability to counter Russian aggression, especially in occupied territories like Crimea and Donbas. Some political and expert debates questioned whether Ukraine needed stronger security guarantees or even to reconsider its non-nuclear status, given Russia’s actions. These discussions underscored broader concerns about the effectiveness of international agreements meant to protect Ukraine.

Russian media promoted a narrative depicting Ukrainian actions as sabotage operations, adopting an increasingly accusatory tone. These reports framed such incidents as proof of Ukraine’s growing hostility and disregard for diplomatic solutions, portraying them as deliberate provocations. This perspective contributed to escalating tensions between the two countries, reinforcing the idea that Ukraine was acting aggressively while justifying Russia’s calls for stronger responses.

August saw a shift in focus toward Belarus, particularly in the wake of the contested presidential election and subsequent protests. Ukraine’s relationship with Belarus became increasingly scrutinised, with some reports framing Belarus as a "second Ukraine," implying that the political unrest there mirrored the 2014 Euromaidan protests. Coverage centred on the opposition, prisoner detentions, and the significant Russian influence over Belarusian politics.

The MH17 case remained a point of contention, with ongoing diplomatic fallout between Russia and Ukraine. The investigation into the downing of the flight continued to draw international attention, particularly as new evidence was presented.

**September**

Diplomatic tensions escalated, mainly in response to the illegal elections held in Crimea. Ukrainian officials condemned the vote as illegitimate, while Russian media framed it as a democratic exercise, dismissing international criticism. Coverage reflected the broader geopolitical divide, with Ukraine seeking to rally Western opposition to Russia’s actions, while Moscow insisted on its sovereignty over the peninsula.

The MH17 trial remained a focal point in media coverage, as proceedings continued against the suspects accused of downing the passenger jet. Ukrainian and Western outlets highlighted the evidence presented against Russian-linked individuals, reinforcing calls for accountability.

Ukraine expanded its military cooperation with Western partners, mainly through joint exercises with NATO forces. Coverage emphasised the significance of these drills in strengthening Ukraine’s defence capabilities.

**October**

Military tensions escalated as Ukraine strengthened its naval capabilities and dealt with border incidents. The media framed these developments as necessary responses to Russian aggression, highlighting efforts to modernise the navy and enhance coastal defences.

Ukraine also intensified its calls for stronger sanctions against Russia, arguing that continued economic pressure was essential to counter Russia’s aggression against Ukraine.

The US presidential election became a focal point, particularly concerning foreign interference and future security policies. Media coverage examined how the election outcome could influence Washington’s stance on Russia and its support for Ukraine.

**November**

Ukraine continued its legal battle against Russia over the 2018 Kerch Strait incident — when Russian forces seized three Ukrainian naval vessels along with 24 sailors. The UN arbitration tribunal took steps in the case, with the media portraying this as a critical move toward holding Russia accountable for its actions.

Moldova’s presidential elections carried implications for Ukraine, particularly concerning the presence of Russian peacekeeping forces in Transnistria. The victory of Maia Sandu, who advocated for the withdrawal of Russian troops, was welcomed in Ukraine as a positive development for regional security.

Propaganda and information operations by Russia remained a key concern, with media outlets highlighting continued disinformation campaigns aimed at undermining Ukraine’s sovereignty and Western support. Reports focused on Russian narratives seeking to discredit Ukraine’s government, exaggerate divisions within the country, and question the legitimacy of international rulings against Russia.

**December**

Ukraine continued to stress the need for strong sanctions against Russia. The media framed sanctions as essential leverage to counter Russian aggression, particularly in Crimea and Donbas.

The Nord Stream 2 pipeline remained a major geopolitical flashpoint, as new US sanctions threatened to derail its completion. Media coverage portrayed the pipeline as a strategic risk, warning that it would increase Europe’s reliance on Russian energy while weakening Ukraine’s role as a key transit country. The conflict in eastern Ukraine showed no signs of resolution, despite diplomatic efforts. Ukrainian officials expressed growing frustration with the Minsk Agreements, questioning their effectiveness in the face of continued ceasefire violations.

The COVID-19 pandemic added new layers of complexity, particularly regarding vaccines and public health responses. The media framed Russia’s promotion of its Sputnik V vaccine as a political tool, with officials rejecting its use in Ukraine and instead seeking Western alternatives.

**2021**

**January**

The conflict in eastern Ukraine remained a key focus. Reports highlighted military clashes and ceasefire violations, with both sides accusing each other of failing to uphold agreements.

Nord Stream 2 remained a contentious issue, with discussions centred on its impact on European energy security and warnings that the pipeline would diminish Ukraine’s role as a key transit country for Russian gas.

The Council of Europe Parliamentary Assembly debated Ukraine-related issues, including human rights concerns in occupied Crimea. Ukrainian representatives called for stronger action against Russian abuses.

Crimean Tatars faced legal challenges in Crimea, with long prison sentences issued by Russian courts, prompting international condemnation.

**February**

Tensions surrounding the conflict in eastern Ukraine remained high. Clashes between Ukrainian and Russian-backed forces, along with continued diplomatic friction, kept the situation volatile.

Nord Stream 2 was a key topic. Critics, including Ukraine and certain European states, expressed concerns that the pipeline would increase Europe’s reliance on Russia for energy, potentially giving Moscow more leverage over European countries, while Russia defended it as a commercial project.

The EU implemented new sanctions against Russia. These sanctions were imposed amid international criticism of Russia’s actions in Ukraine and elsewhere, with particular emphasis on Russia’s annexation of Crimea in 2014 and its continuing role in destabilising the region.

Ukraine’s involvement in the MH17 investigation drew international attention. As legal proceedings continued, with the Dutch government taking a leading role, Ukraine was often in the spotlight due to its control of airspace over eastern Ukraine at the time of the tragedy.

Former Russian President and Prime Minister Dmitry Medvedev publicly criticised Volodymyr Zelenskyy for failing to address the conflict in eastern Ukraine, accusing him of not fulfilling his pre-election promises. However, these remarks reflected a broader disinformation strategy aimed at deflecting blame from Russia’s own role in fuelling the conflict through its invasion and occupation of parts of eastern Ukraine.

**March**

Military activity in Donbas escalated significantly, with both Ukrainian and Russian forces exchanging fire. Reports highlighted numerous ceasefire violations, which exacerbated the humanitarian impact on civilians living in the conflict zone.

Nord Stream 2 remained a focal point. Growing concerns centred on the pipeline’s strategic consequences, especially its potential to undermine Ukraine’s role as a key energy transit hub. The project continued to face opposition from Ukraine, the US, and some European countries, fearing it would increase Europe’s dependency on Russian energy supplies.

NATO-Russia tensions intensified, particularly as discussions on defence spending and Georgia’s NATO aspirations gained prominence. Russia viewed NATO’s growing influence in Eastern Europe, especially regarding Georgia, as a direct threat to its sphere of influence.

The Normandy Format negotiations, aimed at resolving the conflict in Donbas, included discussions on the COVID-19 pandemic and potential vaccine cooperation. While these talks offered a brief opportunity for diplomatic engagement, they were overshadowed by the ongoing conflict and the failure to make substantial progress on key political and security issues.

**April**

A significant military build-up occurred along the Russia–Ukraine border, raising concerns about a potential large-scale conflict. Reports highlighted troop movements and the responses of international actors, with many expressing alarm over the increasing tension.

Diplomatic tensions between Russia and Ukraine continued to intensify, particularly in the realm of espionage. Allegations of intelligence operations and countermeasures emerged, with both countries accusing each other of engaging in covert activities.

The European Parliament took steps to address Russia’s actions, proposing sanctions aimed at pressuring Moscow. Discussions centred around measures targeting Russia’s involvement in regional conflicts and its broader geopolitical strategies. Among the proposed actions were potential sanctions related to SWIFT, which could disrupt Russia’s international financial transactions, and the ongoing Nord Stream 2 pipeline, which was a source of continued debate.

In the investigation into the MH17 crash, new evidence emerged, further implicating Russian actors in the downing of the Malaysian Airlines flight. This development added weight to the legal case against those responsible and drew renewed attention to Russia’s role in the incident.

**May**

Border tensions between Russia and Ukraine remained a central concern, with ongoing military activities on both sides. Diplomatic efforts to prevent escalation were a priority for international actors, as the risk of further confrontation loomed large.

The Russia–Ukraine gas dispute intensified, affecting European energy markets and sparking broader geopolitical debates. As Russia continued to reduce gas supplies through Ukraine, concerns grew over the reliability of Russian energy exports to Europe. This dispute also underscored the wider geopolitical struggle, with Ukraine and its allies urging Europe to diversify energy sources to reduce dependency on Russia.

Crimea’s water crisis became an international issue, drawing attention to Russia’s control of the region and its impact on local populations. Discussions on the crisis highlighted contrasting positions from Russia and the US, with Moscow emphasising the region’s status as part of Russia, while the US and Ukraine called for accountability and international pressure to address the humanitarian situation.

The US Secretary of State visited Ukraine, reaffirming Washington’s support for Kyiv in the face of Russian aggression. During his visit, he addressed anti-corruption measures, underlining the importance of governance reforms for Ukraine’s future stability and relations with the West. The visit served to strengthen ties between the two countries and reaffirm Ukraine’s strategic alignment with Western institutions.

**June**

Ukraine-NATO relations took centre stage, with discussions focusing on Ukraine’s membership prospects and the security implications of closer ties. While Kyiv pushed for progress towards NATO integration, Russia strongly opposed any expansion, warning of potential consequences.

The MH17 trial saw further developments as prosecutors presented new evidence linking a Russian missile system to the 2014 disaster. The findings reinforced international accusations of Russian involvement, increasing pressure on Moscow while further straining its relations with the Netherlands and other Western countries.

Ukraine’s relations with Russia and Belarus grew more complex amid regional instability and diplomatic manoeuvres. Belarus’s increasing alignment with Moscow, particularly following international sanctions against the Lukashenko regime, influenced the dynamics between the three countries. Ukraine remained cautious of growing Russian influence in Belarus, viewing it as a potential threat to regional stability.

Russian passport distribution in Donbas continued to fuel tensions, with Moscow granting citizenship to more residents in the occupied territories. Ukraine and its Western allies condemned the move as a violation of sovereignty and a tool for further entrenching Russian influence in the region. The issue raised concerns over security risks and potential justifications for future Russian intervention.

**July**

Tensions in the Black Sea escalated as both NATO and Russia conducted military exercises in the region. These manoeuvres underscored the strategic importance of the Black Sea, with NATO demonstrating support for Ukraine while Russia framed its actions as necessary for regional security. The military activities heightened concerns about potential clashes and further militarisation of the area.

Nord Stream 2 remained a key geopolitical issue, with discussions intensifying after the US-Germany agreement addressing concerns over Russian energy influence in Europe. While the deal included provisions to support Ukraine’s energy security, critics argued that it failed to sufficiently counterbalance Russia’s leverage over European gas supplies. Ukrainian officials voiced strong opposition, fearing long-term economic and security consequences.

Russia’s legal complaint against Ukraine at the European Court of Human Rights attracted widespread media coverage. Moscow accused Kyiv of human rights violations, while Ukraine and its allies dismissed the case as an attempt to deflect from Russia’s crimes in Crimea and Donbas.

Putin’s rhetoric denying Ukraine’s nationhood sparked controversy. His claims were widely criticised as an attempt to justify Russian influence over Ukraine. Some analysts compared his arguments to Hitler’s rhetoric on the Sudetenland, suggesting a dangerous precedent for territorial ambitions.

**August**

Diplomatic and military frictions between Russia and Ukraine continued. Ukrainian officials voiced concerns over Russia’s aggression in Donbas and Crimea, while Russia accused Ukraine of escalating hostilities. Media coverage highlighted the lack of progress in diplomatic efforts to ease tensions.

The imminent completion of Nord Stream 2 prompted further discussions in the US and Europe on potential countermeasures. While Germany and the US had reached an agreement aimed at mitigating Ukraine’s economic losses, Kyiv continued to warn that the pipeline would increase European dependence on Russian gas and weaken Ukraine’s strategic position.

Ukraine sought additional military aid from the US as a response to Russian aggression. Ukrainian officials emphasised the need for defensive capabilities, citing ongoing threats from Russian forces. Reports emerged that Donbas residents holding Russian citizenship had participated in Russia’s parliamentary elections. This development raised legal and political concerns, with Kyiv condemning it as a violation of Ukrainian sovereignty.

**September**

US-Ukraine relations remained a key topic, with Washington reaffirming its support amid ongoing tensions with Russia. High-level meetings and diplomatic statements underscored continued military assistance, as Ukraine sought further security guarantees against Russian aggression.

The MH17 investigation advanced, with new testimonies and evidence emerging. Reports focused on witness accounts and forensic findings that further implicated Russian-backed forces.

Ukraine imposed a ban on Transnistrian-registered vehicles, citing concerns over legal status and border security. The move disrupted regional trade and heightened tensions with Moldova and Russia, as Moscow condemned the decision as an economic blockade.

Ceasefire violations in Donbas persisted, with reports of skirmishes and civilian casualties.

**October**

The Russia–Ukraine gas transit dispute took centre stage, contributing to volatility in European energy markets. Ukraine accused Russia of using energy as a geopolitical weapon by reducing gas transit through Ukrainian pipelines, while Russian officials insisted on market-driven decisions. European leaders expressed concerns about energy security and debated potential countermeasures.

The Normandy Format negotiations saw renewed diplomatic efforts to resolve the conflict in eastern Ukraine, but progress remained limited. Talks between Ukraine, Russia, France, and Germany failed to yield significant breakthroughs, with each side blaming the other for the stalemate.

Russian military exercises in Crimea heightened concerns about regional security and Ukraine’s territorial integrity. Ukrainian officials condemned the exercises as provocative, while Moscow presented them as routine defensive manoeuvres.

NATO updated its defence strategy with a new Master Plan, aimed at responding to potential Russian threats. The plan emphasised deterrence and defence, with measures to strengthen NATO’s military posture and cyber defences, particularly in response to Russian activities in the Baltic and Black Sea regions. Russian officials criticised the plan as an unnecessary escalation and an attempt to justify NATO expansion.

Ukrainian Telegram channels promoting pro-Russian narratives gained attention, raising concerns about FIMI. Analysts highlighted how certain channels amplified Kremlin-aligned messaging, influencing public opinion in Ukraine.

**November**

Tensions escalated along the Russia–Ukraine border, with reports of Russian military build-ups raising international alarm over a potential invasion. Satellite images and intelligence assessments indicated significant troop deployments near Ukraine, prompting warnings from Western governments. Ukrainian officials described the build-up as an act of intimidation, while Moscow dismissed concerns, framing the movement as routine exercises.

US President Joe Biden and Russian President Vladimir Putin engaged in diplomatic discussions as US-Russia relations deteriorated over Ukraine. The talks aimed to prevent further escalation, with the US warning of severe economic consequences if Russia launched an invasion.

Espionage and cyberattacks linked to Russia targeted Ukrainian institutions. Ukrainian officials reported an increase in cyber intrusions against government agencies, financial institutions, and critical infrastructure. Western intelligence agencies attributed these actions to Russian state-backed groups, viewing them as part of a broader hybrid warfare strategy.

Attacks by Russian-backed forces in eastern Ukraine intensified, increasingly affecting civilian areas and raising serious humanitarian concerns. Reports documented heavy shelling in Donetsk and Luhansk, resulting in casualties among both military personnel and civilians.

**December**

US President Joe Biden and Russian President Vladimir Putin held high-level talks to address rising tensions over Ukraine. The discussions were centred on the growing military presence along the Ukrainian border and the potential for further escalation. Biden warned of severe consequences if Russia invaded Ukraine, while Putin emphasised NATO’s eastward expansion as a primary concern.

The gas crisis intensified as Russia reduced gas flows through Ukraine’s pipelines, exacerbating energy shortages in Europe during winter. European countries, particularly those reliant on Russian gas, sought alternatives, and debates around Nord Stream 2 and energy diversification took centre stage.

The MH17 trial made significant progress, with prosecutors seeking life sentences for those implicated in the downing of the flight. The trial continued to unravel evidence linking the missile used to Russian-backed separatists in eastern Ukraine.

Black Sea tensions escalated, with increased naval incidents involving Ukraine, Russia, Georgia, and NATO forces. Reports emerged of close encounters between Russian warships and NATO vessels, heightening fears of an inadvertent conflict. Both sides blamed each other for the provocations, with Russia accusing NATO of military encroachment and Ukraine stressing its right to defend its waters.

Diplomatic efforts failed to ease mounting hostilities between Russia and Ukraine. Despite ongoing talks and international mediation, the situation in Donbas and Russia’s growing military presence near Ukraine’s border continued to fuel tensions. Ukrainian officials warned of a looming threat of invasion, while Russia maintained its stance on NATO’s role in the conflict. The international community remained divided on how to address the crisis, with some calling for stronger sanctions and others advocating for renewed diplomatic engagement.

**2022**

**January**

Reports frequently highlighted escalating tensions between Russia and the EU, as well as the deepening crisis in Ukraine and the increasingly fraught NATO-Russia relations.

A key focus was Russia’s growing military presence near Ukraine, which led to significant coverage of debates within Russia’s State Duma regarding the potential recognition of separatist regions in eastern Ukraine. This raised alarm over the territorial integrity of Ukraine and the risk of further territorial encroachment by Russia.

Disinformation campaigns played a central role, particularly surrounding false reports about Ukrainian mines. These stories highlighted Russia’s use of hybrid warfare tactics, with Russian officials pushing a narrative framing the West as an aggressive force, while downplaying Russia’s actions and justifying its military build-up as a defensive move.

The media also covered the broader geopolitical ripple effects, such as the increasing tensions between Kosovo and Serbia. The unrest in Kazakhstan was also frequently reported, underscoring the instability Russia’s actions were contributing to across former Soviet states. Moldova and the Transnistria region were also drawn into the discourse, as media coverage noted growing concerns about Russia’s influence and the potential for further regional instability.

Russia’s control over European energy supplies remained a significant topic, with reports drawing attention to the gas crisis and highlighting how Russia’s energy leverage was a powerful tool in the broader geopolitical struggle.

**February**

Media coverage escalated dramatically as Russia launched a full-scale invasion of Ukraine on 24 February. Reports focused on the advance of Russian troops around Kyiv and across Mariupol, triggering widespread global condemnation. The invasion became the dominant narrative, highlighting the human toll and the resilience of Ukrainian forces as millions were displaced.

Cyberwarfare received significant attention, with reports detailing Russian cyberattacks on Ukrainian infrastructure. In response, hacker group Anonymous and other cyber-activists launched retaliatory strikes against Russian targets.

The media drew comparisons between Russia’s invasion of Ukraine and the potential Taiwan-China crisis, particularly with the visit of a US delegation to Taiwan, showcasing rising tensions in the Asia-Pacific region.

As the invasion intensified, Sweden and Finland grew increasingly concerned about their security, leading to renewed discussions about NATO membership.

**March**

Media coverage focused on the devastating humanitarian crisis, particularly in Mariupol, which became a symbol of the war’s toll on civilians. Reports detailed severe casualties and widespread suffering as Russian forces besieged the city, capturing global attention.

Millions of Ukrainian refugees fled to neighbouring countries, placing immense pressure on regional resources. Media discussions highlighted the varying responses of Western nations to the refugee crisis, often drawing comparisons between refugees from Ukraine and those from other conflict zones, such as the Middle East and Africa.

Tensions in the wider region also garnered attention. Kosovo’s stability became a recurring topic, especially in relation to the EU and NATO’s involvement in Ukraine. Media also noted the growing concern in Georgia regarding its geopolitical alignment with Ukraine and the possibility of increased Russian pressure. Belarus’s military mobilisation further intensified these concerns, with experts warning of potential regional escalation.

Geopolitical developments continued to unfold, as countries navigated their positions on Russia’s war against Ukraine. In Israel, media coverage highlighted the country’s dilemma in balancing Western sanctions with its complex relationship with Russia, particularly amid growing pressure to offer refuge to Russian Jewish oligarchs. Meanwhile, analysts turned their focus to China, exploring its potential role in mediating the conflict due to Beijing’s close ties with both Russia and Ukraine. At the same time, Taiwan reconsidered its military service policies, reflecting rising concerns about the implications of Russia’s invasion of Ukraine for its own security, especially in the context of increasing tensions with China.

**April**

Media coverage increasingly reflected Russia’s attempt to control the narrative of its invasion, with the term “special military operation” gaining prominence in official discourse. Reports highlighted how this language was used by Russian officials and state-controlled media to downplay the scale of the conflict and frame it as a justified intervention rather than an outright war.

Georgia’s position on the war received significant attention, with extensive media discussion on the government’s response to Russian aggression. Coverage focused on the political tensions within Georgia over its stance, as well as broader implications for its relationship with NATO and the West.

The role of the alliance remained a key theme, with reports also exploring the significance of NATO membership for North Macedonia in the context of growing security concerns in the region.

Disinformation and propaganda continued to be central to the media narrative, with widespread reports on Russia’s efforts to manipulate public perception of the war. Coverage detailed fabricated stories about planned false flag attacks, the circulation of fake surrender videos, and the involvement of Russian diplomats in spreading disinformation on social media. Reports also examined how Russian propaganda channels were replacing international news outlets such as Euronews in certain regions, ensuring that Kremlin-backed narratives remained dominant. Another major focus was the targeting of young audiences, with analysts warning of efforts to instil pro-Russian sentiment among youth through carefully crafted messaging.

**May**

Media coverage continued to revisit familiar narratives, including Georgia-Ukraine relations, with the Georgian government facing scrutiny over its cautious stance on the invasion; Moldova’s ties with Russia, with concerns growing over Russian influence in Transnistria and potential destabilisation; the impact of sanctions, as debates persisted over their effectiveness in curbing Russia’s aggression; and Belarus’s role in Russia’s war against Ukraine, with reports highlighting its support for Moscow and speculation about deeper military involvement.

A key focus was Russia’s response to accusations of war crimes in Bucha, with the Kremlin demanding an independent inquiry while dismissing Ukraine’s claims as staged propaganda. This was widely interpreted as an attempt to deflect attention from allegations against Russian forces and cast doubt on the credibility of the evidence presented by Ukraine and its allies.

**June**

Media discussions on Georgia’s NATO and EU aspirations gained prominence, highlighting ongoing debates about the country’s Western integration and its broader geopolitical significance. At the same time, Georgia’s refusal to impose sanctions on Russia sparked controversy, with the government defending its decision as a necessary measure to protect economic stability. While Western nations criticised this stance for undermining collective pressure on Moscow, some narratives framed it as a pragmatic approach to safeguarding national interests. In contrast, Moldova was portrayed as more closely aligned with Western policies, reinforcing perceptions of a growing divide among post-Soviet states in their responses to the war.

Reports highlighted growing concerns over Russia’s military support to Belarus, particularly regarding the potential transfer of nuclear-capable missiles.

**July**

Russian disinformation narratives continued to circulate, with claims that Western nations and Turkey were transferring terrorists from Syria to Ukraine. These allegations, reportedly made by Syria’s ambassador, aligned with broader Kremlin efforts to discredit Western support for Ukraine. At the same time, Russian authorities intensified their crackdown on independent media, blocking access to outlets such as *Novaya Gazeta* and *Die Welt*, underscoring Moscow’s drive to monopolise domestic information channels and control war-related narratives.

Russian propaganda also sought to shift blame for the global food crisis onto the West, particularly in Africa, where narratives framed Western sanctions as responsible for supply chain disruptions. This reflected Moscow’s ongoing efforts to expand its influence beyond the war zone.

Finally, media attention turned to allegations against Andriy Yermak, President Zelenskyy’s chief of staff. Some narratives suggested that Yermak was involved in misconduct or was undermining Ukraine’s interests, with speculation ranging from accusations of corruption to claims that he was secretly working in Russia’s favour. While no concrete evidence emerged, the allegations fed into broader disinformation efforts aimed at sowing distrust within Ukraine’s leadership and casting doubt on the government’s wartime decision-making.

**August**

Media coverage focused on the growing concerns surrounding the Zaporizhzhia nuclear power plant, where military operations near the facility raised fears of a nuclear disaster.

Another significant topic was the attack on Daria Dugina, which underscored how the war was no longer confined to Ukraine’s territory but was increasingly shaping geopolitical dynamics well beyond the immediate battlefield.

Russia worked to bolster national pride through narratives celebrating its military as "heroes," framing the "special military operation” in a positive light to maintain domestic support. This was part of a larger effort to promote the war as a heroic national struggle. Russian propaganda efforts were expanding into the EU, with reports highlighting the Kremlin’s attempts to infiltrate European media and influence public opinion. This was accompanied by growing concerns about Russian espionage activities, particularly in Germany, as the war continued to ripple through European politics.

Energy politics also came into focus, with protests in Bulgaria highlighting the country’s dependence on Russian natural gas and the challenges the EU faced in balancing energy needs with its broader stance on Russia.

**September**

A key topic of discussion was Russia’s effort to strengthen its control over Ukraine’s eastern territories, illegally occupied by Russia since 2014, which it attempted to solidify through referenda and reintegration. This strategy complicated diplomatic efforts aimed at resolving the war.

Another prominent narrative emerged around the claim that a significant portion of the world supported Russia, highlighted during the Shanghai Cooperation Organisation (SCO) summit. This framing reflected Russia’s attempts to strengthen ties with countries in the SCO as part of its broader strategy to counter Western influence.

Ukraine’s efforts to combat disinformation gained attention, particularly in the case of a Hungarian geography textbook that was accused of containing false information about Ukrainian territory. This highlighted Ukraine’s proactive stance in challenging misrepresentations of its borders and asserting its sovereignty on the international stage.

Finally, media coverage pointed to the shifting energy dynamics in Europe. The term “profiting” was used to describe Norway’s position, but this raised ethical concerns, as it oversimplified the complex geopolitical and economic factors involved in the energy crisis exacerbated by the war.

**October**

Media coverage focused on the adoption of Ukrainian children by Russian families, highlighting forced transfers, lack of parental consent, and violations of international law, and portraying it as a potential war crime.

The explosion on the Kerch Strait Bridge in Crimea drew significant media attention. The incident was widely reported as a symbolic blow to Russian infrastructure and a potential escalation point in the war.

Disinformation remained a key tool. Russian media campaigns amplified manipulated videos and reports, reinforcing Kremlin-controlled messaging while undermining alternative sources.

Religious influence emerged as a geopolitical tool, as reports surfaced about the Russian Orthodox Church acquiring property near Norwegian military bases. While framed as cultural expansion, this raised security concerns among NATO allies, who viewed it as a potential intelligence-gathering effort.

Russian state-controlled media promoted referenda in occupied territories as legitimate expressions of self-determination. While widely condemned as coerced and illegal, these votes were framed as proof of popular support for integration with Russia.

**November**

Media coverage was dominated by the liberation of Kherson, a major turning point in the war. The retreat of Russian forces marked a significant Ukrainian victory, widely covered in Western media as a blow to Moscow’s military ambitions. Russian official discourse, however, sought to downplay the event, portraying it as a strategic withdrawal.

Another key topic was North Korea’s denial of arms deals with Russia following US accusations of military cooperation. While Western reports framed this as evidence of Russia seeking external support for its war effort, Russian and North Korean sources dismissed the claims as baseless.

The EU’s decision to reject Russian passports issued in occupied Ukrainian and Georgian regions underscored its commitment to upholding international law. Western media framed this as a firm stance against Russia’s attempts to legitimise territorial control.

The role of Chechen fighters in Ukraine’s war highlighted the complex motivations driving individuals into combat. Coverage explored their struggle for independence and opposition to Russian rule, with Western media often framing them as symbols of resistance.

Russian media promoted its offer of medical study opportunities for Indian students displaced by the war. This was framed as a humanitarian gesture, positioning Russia as a benefactor amid the conflict, while international coverage viewed it as an attempt to maintain influence and deflect criticism.

**December**

Media coverage highlighted growing military and strategic ties between Russia and its key allies. Reports on Iran-Russia military cooperation highlighted Iran’s supply of weapons used by Russian forces in Ukraine, with Western sources framing this as further evidence of Tehran’s deepening involvement in the war. Russian official discourse, however, downplayed these claims, emphasising mutual security interests.

At the same time, coverage of Russia-China relations depicted strengthening cooperation amid the ongoing crisis. Western media interpreted this as Beijing offering Moscow diplomatic and economic backing without full military support, while Russian outlets portrayed the partnership as a stabilising force against Western pressure.

**2023**

**January**

The war continued to shape European energy security, with the gas crisis emerging as a central issue, highlighting Europe’s struggle to diversify energy sources amid the war.

The tense situation at the Zaporizhzhia nuclear power plant remained a significant concern, with escalating risks as both sides presented conflicting narratives about control and safety.

Finland and Sweden formally applied for NATO membership, a move seen as a direct response to Russia’s aggression in Ukraine, with both countries seeking to bolster their security in the face of growing Russian threats. Russia, however, strongly opposed the Scandinavian countries’ accession, framing the expansion as a provocative move, accusing NATO of exacerbating tensions and undermining stability in the region.

An emerging narrative was Ukraine’s corruption crisis amid the war, with government shakeups and anti-graft measures drawing attention. The focus on Ukraine’s internal struggles highlighted how corruption intertwined with the broader conflict, with Western media examining the impact on international support and Russia exploiting these weaknesses for propaganda.

**February**

Media coverage focused on the Bakhmut massacre, a grim episode in the ongoing war that symbolised the intensity of the fighting and its devastating human toll.

The one-year mark since the start of Russia’s full-scale invasion of Ukraine drew significant media attention. Coverage focused on the ongoing impact of the war, the resilience of the Ukrainian resistance, and the international community’s response. Media outlets reflected on the war’s humanitarian and geopolitical consequences, offering analyses of its progression over the past year and speculating on future developments in the conflict.

Western media analysed Russian disinformation campaigns, with particular attention on Twitter’s incomplete disinformation report, which prompted a rebuke from the EU due to concerns about the platform’s role in spreading misinformation. Pro-Russian propaganda accounts exploited Twitter’s verification system to amplify war-related falsehoods and anti-Western sentiment. This was part of a broader strategy that included revisiting the timeline of the Russia–Ukraine war and the role of influential figures, such as television personality Vladimir Solovyov, in spreading Russian political messaging.

**March**

A significant development was Russia’s decision to declare Melitopol as the new capital of the occupied Zaporizhzhia region. This move was seen as an attempt to solidify Russia’s control over the area and further its annexation efforts.

Tensions surrounding the Kyiv-Pechersk Lavra, a key religious site in Ukraine, escalated due to the conflict between the Ukrainian Orthodox Church of the Moscow Patriarchate (UOC-MP) and that of Ukraine (UOC-UA). The Ukrainian government seeking to regain control of religious institutions tied to Moscow led to clashes over the Lavra’s control, symbolising the broader struggle for Ukraine’s independence and cultural identity amid the war.

Propaganda and disinformation continued to shape the narrative, with European leaders issuing a joint call for tech companies to intensify efforts against the spread of false narratives online. One particularly bizarre claim from Russian sources suggested that Britons were eating squirrels due to economic hardship linked to their support for Ukraine, aiming to undermine Western solidarity.

**April**

Incidents along the Russo-Ukrainian border increased, with accidental military actions raising the risk of further escalation.

Tensions within Ukraine grew, particularly within the Ukrainian Orthodox Church, which faced accusations of ties to Moscow.

Russia’s internal propaganda took a striking turn with the removal of references to Kyiv in Russian school textbooks about Kyivan Rus.

Social media became another battleground as a former US Navy officer was exposed for running the pro-Russian account “Donbass Devushka,” which spread disinformation and leaked sensitive documents.

**May**

Media coverage centred on Russia’s announcement of plans to deploy tactical nuclear weapons to Belarus, a move that President Putin justified as a response to NATO’s presence in Eastern Europe and Western military support for Ukraine. The decision raised alarm in Western capitals, with widespread condemnation from NATO allies and concerns about the destabilising impact on regional security.

Georgia’s decision to resume direct flights with Russia sparked protests from opposition groups and prompted criticism from the EU. The move was seen as undermining Georgia’s pro-Western stance, which had been solidified after Russia’s invasion of Ukraine.

The breakaway region of Transnistria appealed to Russia for more “peacekeepers.” The appeal for more peacekeepers was framed as necessary to ensure stability and protect the region’s security amid fears of escalation, especially as Moldova expressed increasing concerns over the conflict’s impact on its sovereignty.

On the internal front in Russia, partisans and the Legion of Freedom, a group that opposes Putin’s regime, reportedly intensified recruitment for attacks on Moscow, signalling growing resistance to Putin’s regime.

Reports emerged that Ukrainian troops were facing significant challenges on the front lines, mainly due to lack of training and adequate equipment. While some reports framed these issues as part of Ukraine’s heroic resistance against a far larger and better-equipped Russian military, other outlets raised concerns about how these shortcomings might affect Ukraine’s ability to sustain its defence in the long term.

**June**

Media coverage highlighted the catastrophic destruction of the Kakhovka Dam, which resulted in a severe environmental and humanitarian crisis.

Yevgeny Prigozhin, the head of the Wagner Group, a Russian paramilitary organisation, launched an armed mutiny against the Russian military leadership, which culminated in Wagner forces marching toward Moscow. The uprising underscored growing internal tensions within Russia, calling into question the stability of President Putin’s regime.

Bulgaria faced scrutiny over its links to the Wagner Group due to concerns about possible ties between Bulgarian nationals and the group. In response, Bulgaria’s government advocated for diplomatic engagement with Russia and considered withdrawing from any direct involvement with Wagner.

In Moldova, the government banned the pro-Russian Shor Party following protests and a Constitutional Court ruling, signalling the country’s commitment to safeguarding its sovereignty amidst external pressures.

**July**

Tensions between the Wagner Group and the Russian military continued to escalate. After the mutiny, Wagner forces were allowed to operate in Belarus, but there were ongoing reports of distrust between the two sides. The Russian government attempted to downplay these tensions.

Belarus prepared for potential conflict, as indicated by its emergency ministry’s training and armament efforts, signalling regional escalation risks.

Romania called for a permanent German troop presence as part of NATO’s regional security reinforcement against Russian aggression.

Disinformation and propaganda continued to spread, with Russian actors dismissing environmental concerns about Black Sea pollution in Bulgaria as unfounded. Russian propaganda infiltrated video games, with certain games’ storylines spreading pro-Russian views or distorting facts about the ongoing conflict.

**August**

The media reported on heated debates about Ukraine’s potential NATO membership. The discussions focused on the implications of Ukraine joining NATO and the territorial concessions that might be required in return. Russian official discourse depicted the potential inclusion of Ukraine in NATO as a direct challenge to Russia’s geopolitical interests and security. At the same time, Finland and Sweden’s official NATO membership further strengthened the alliance’s northern security posture.

Russia’s forced citizenship policy in occupied Ukrainian territories deepened tensions and escalated the conflicts by further undermining Ukraine’s sovereignty and deepening ethnic and political divides in the affected regions.

Concerns over the safety of the Zaporizhzhia nuclear power plant and Nord Stream pipeline remained prevalent, as the international community continued to monitor these critical assets amid the ongoing war. In Belarus, the Wagner Group’s registration as an educational organisation added a new dimension to Russian propaganda.

Russian propagandists promoted territorial ambitions beyond Ukraine.

**September**

Russian disinformation campaigns, particularly in Germany, were noted as intensifying, with a focus on influencing public opinion about the war. German counterintelligence experts highlighted these efforts, marking a significant push to manipulate perceptions in Western Europe. Reporting on the fighting in the Donbas region, Russian media pushed the terminology of a “conflict” rather than an “invasion,” a strategy designed to downplay the severity of the situation and undermine Ukraine’s sovereignty. By using this language, Russian outlets sought to present the war as a more ambiguous, less aggressive event. This framing also helped portray the war as a domestic issue or regional dispute, rather than an outright invasion of a sovereign nation.

Criticism of Ukraine’s leadership surfaced, particularly concerning the loss of Kupyansk and Russian advancements in the Kharkiv region. These setbacks were weaponised by Russian media to attack President Zelenskyy’s political career, portraying his position as increasingly precarious and highlighting Russia’s apparent military gains.

**October**

Media discussions focused on growing tensions in Kosovo, with the country’s Foreign Minister warning that continued international support for Serbia without addressing Kosovo’s concerns could lead to war. This reflected the increasingly fragile peace in the region, with concerns that unresolved issues between Kosovo and Serbia could escalate into conflict if international diplomacy failed to intervene effectively.

At the same time, there was growing discourse within the EU regarding Ukraine’s potential membership. Discussions highlighted Ukraine’s significant financial challenges, particularly its mounting debts, and raised questions about how these financial strains might impact Ukraine’s ability to join the EU. This narrative framed the EU’s support for Ukraine, particularly for President Zelenskyy during the war, in a way that cast doubt on Ukraine’s economic stability and its readiness for integration into the European bloc.

**November**

Media coverage centred on heightened tensions between Finland and Russia, driven by a growing migration crisis. As the war in Ukraine continued, many Russian citizens sought to flee the country, fleeing repression and the impact of sanctions. This mass movement of people was compounded by the increasing fears of a new “Iron Curtain” effect, as Finland began to tighten its border controls and Russia imposed stricter travel restrictions.

The FIMI landscape saw new efforts to counter Russian propaganda. Reporters Without Borders launched a Russian-language satellite news package called “Freedom,” aimed at providing independent news and information to Russian-speaking audiences, counteracting the narratives put forward by the Kremlin.

**December**

Disinformation and propaganda remained key themes, with particular focus on Russian efforts to shape global narratives about the war in Ukraine. The EU’s response to these disinformation campaigns included fining Google for failing to adequately tackle fake accounts and misleading content related to the war. Concerns grew over the influence of both Russian and Chinese propaganda in Eastern Europe, especially in Poland and the Czech Republic. These disinformation campaigns were aimed at influencing political attitudes and decision-making, ultimately seeking to erode EU unity and undermine the West’s commitment to Ukraine’s sovereignty and its support for the war effort against Russia.

**2024**

**January**

Discussions on Western military support to Ukraine remained prominent, with particular attention given to President Zelenskyy’s evening addresses to the Ukrainian population and allied nations. The EU and NATO reaffirmed their commitment to Ukraine’s sovereignty and territorial integrity, reinforcing their continued backing amid the war.

Reports detailed the largest prisoner exchange since the full-scale invasion in February 2022. Over 200 individuals from each side were released in a swap facilitated by the United Arab Emirates.

Russia’s claim of targeting a group of French mercenaries in Kharkiv sparked debate. The Russian Ministry of Defence reported a precision strike on a building, alleging that over 60 individuals had been killed.

Missile and drone attacks also featured heavily in coverage, with reports highlighting the state of Ukraine’s air defences, Russia’s use of North Korean missiles, and civilian casualties in targeted Ukrainian cities.

Another significant event was the crash of a military plane in Belgorod, which had been carrying 65 Ukrainian prisoners of war (POWs) for an exchange. The incident led to widespread speculation and debate over responsibility for the downing.

**February**

The interview between Russian President Vladimir Putin and US journalist Tucker Carlson attracted widespread international attention. The discussion covered sensitive topics, including the war in Ukraine, Russia’s diplomatic relations with the West and NATO, and prisoner exchanges with Ukraine, prompting extensive analysis and reactions globally.

Sanctions against Russia remained a key issue. The US, the EU, and the UK announced a new package of coordinated sanctions targeting Russia’s financial sector, energy production, and the military-industrial complex. Discussions intensified around the G7’s consideration of using frozen Russian assets to support Ukraine’s aid and reconstruction efforts. The imposition of sanctions in response to the death of opposition leader Alexei Navalny also featured prominently in coverage.

Debates among European nations over their level of involvement in Ukraine’s defence gained momentum, particularly regarding the potential deployment of NATO troops. Former US President Donald Trump’s comments on NATO also drew attention, as he urged Member States to increase their defence spending to five per cent of their GDP, up from the current two per cent target.

The war itself remained at the centre of coverage, with a specific focus on the battle for the Ukrainian city of Avdiivka.

**March**

Russia’s internal affairs received extensive coverage, particularly following the terrorist attack on Moscow’s Crocus City Hall. The attack sparked debates over Russia’s security vulnerabilities and the potential resurgence of international terrorism. The death of Alexei Navalny was a focal point, with his funeral drawing thousands of mourners across Russia. The gatherings, which also served as acts of protest, underscored the ongoing domestic opposition to President Vladimir Putin’s regime. The re-election of Vladimir Putin garnered significant media attention, as it raised questions about the future direction of Russian domestic policies and its stance on international issues amid ongoing tensions and internal dissent.

The debate over NATO countries’ support for Ukraine intensified amid escalating tensions between Russia and Western nations. Discussions focused on the West’s increased military assistance to Ukraine, including considerations about the possible deployment of troops to reinforce Ukraine’s defence and sovereignty. The provision of F-16 fighter jets was also widely discussed. Meanwhile, concerns grew over Russia’s heightened nuclear rhetoric and its broader implications for global security.

New sanctions against Russia were introduced, particularly in response to its deepening military ties with Iran and North Korea. Reports highlighted Iran’s supply of ballistic missiles and North Korea’s provision of artillery shells to Russia, further fuelling tensions between Moscow and the West.

**April**

Russia’s war against Ukraine remained at the forefront of international attention. Coverage focused on drone and missile attacks targeting Ukrainian cities and civilians, military developments on the battlefield, the growing toll of military losses, and concerns over a potential accident at the Zaporizhzhia nuclear power plant.

Western military aid to Ukraine, particularly from NATO countries, remained a central topic. The provision of ATACMS missiles and other weaponry by the US was repeatedly highlighted, alongside strong commitments from the EU, the UK, and the US to continue diplomatic and military support.

Related discussions on NATO-Russia relations also featured prominently, especially in the context of NATO’s 75th anniversary and the risks of nuclear escalation. The candidacy of Mark Rutte for the position of NATO Secretary-General was also a subject of debate.

The terrorist attack on Moscow’s Crocus City Hall continued to be a focus, with further details emerging about the Russian authorities’ investigation, potential suspects, and possible geopolitical ramifications.

**May**

Ukraine’s use of Western-supplied weapons to strike targets within Russian territory led to notable shifts in international policy and military strategy. Tensions between NATO and Russia in the Baltic region were also covered, with a focus on regional defence initiatives and an enhanced military presence aimed at strengthening security along NATO’s eastern flank. Meanwhile, Russia’s renewed offensive near Kharkiv was widely reported, marking an intensified effort to gain control over the region, which resulted in significant civilian casualties and infrastructure damage.

Peace talks and diplomatic efforts were also a topic of discussion, particularly the organisation of a peace summit in Switzerland, which aimed to foster dialogue and explore potential pathways to resolving the conflict. Additionally, Donald Trump’s proposed peace plan was covered, outlining a strategy that combined diplomatic initiatives with the strategic use of military assets to pressure both Russia and Ukraine into negotiations.

Sanctions against Russia remained a key focus, as international measures continued to intensify. Notably, the EU approved the use of revenues from frozen Russian assets to support Ukraine, signalling a commitment to leveraging economic tools for geopolitical objectives.

**June**

A debate emerged over Viktor Orbán’s diplomatic engagements, which aimed to address Russia’s war against Ukraine and influence EU-Russia relations. His unilateral actions and meetings with Russian leadership, conducted without EU coordination, drew significant criticism.

The first Global Peace summit for Ukraine, held in Switzerland on 15–16 June, was widely covered. The summit brought together representatives from 92 nations and eight international organisations, though Russia was not invited to participate.

Another major development was the EU’s launch of accession negotiations with Ukraine and Moldova, marking a pivotal moment in European geopolitics and reinforcing the EU’s commitment to supporting these nations amid ongoing challenges.

Military aid to Ukraine remained a key topic. The US, alongside its allies, announced a $400 million aid package to strengthen Ukraine’s defence capabilities.

Russian territorial gains continued to be a focal point. Advances in the Donetsk and Kharkiv regions highlighted the sustained intensity of the conflict and underscored the strategic importance of these areas in the broader war effort.

**July**

Hungarian Prime Minister Viktor Orbán’s “peace mission” drew significant attention. Orbán’s diplomatic visits aimed to restore communication channels between Russia, Ukraine, and Western powers, sparking controversy within the EU. Media outlets highlighted the friction between Hungary’s independent stance and EU unity, with Orbán’s efforts portrayed as either undermining collective European diplomacy or as a pragmatic attempt to mediate peace.

The NATO summit in Washington was another focal point, with discussions on Ukraine’s potential membership and defence spending dominating the agenda. Western media framed the summit as a milestone for alliance cohesion, emphasising commitments to bolster Ukraine’s defence capabilities. In contrast, Russian official discourse criticised NATO’s expansion and arms support as provocative, exacerbating the conflict.

Russia’s war against Ukraine saw further escalation, marked by drone attacks on infrastructure and a tragic strike on a Ukrainian children’s hospital. International condemnation of the hospital attack highlighted growing civilian casualties.

Economic and energy disputes resurfaced as a point of contention. Central European nations, including Ukraine, faced challenges related to Russian oil transit and sanctions. The media framed these disputes as reflective of broader economic pressures stemming from the protracted war, with Ukraine seeking alternative solutions to reduce dependence on Russian energy supplies.

**August**

Russia’s war against Ukraine saw continued escalation, with military operations and significant developments along the Kursk, Sumy, and Zaporizhzhia regions. Ukrainian drone and missile attacks targeted Russian cities and military sites, with reports of increasing civilian casualties. The Kursk nuclear power plant, specifically, became a flashpoint, with incidents raising concerns about radiation safety and international investigations into the potential risks of the war spreading to nuclear facilities.

Western military support for Ukraine remained a prominent issue, with the delivery of F-16 fighter jets seen as a major boost to Ukraine’s air defence capabilities. This development sparked concerns in Russia about the possibility of a broader regional conflict, with many media outlets framing the support as a potential catalyst for World War III.

The economic side of the conflict also remained in the spotlight, with tensions surrounding the Nord Stream pipeline sabotage investigation and allegations of complicity involving the EU. The energy crisis also affected Ukraine, Slovakia, and Hungary, leading to political rifts and debates over EU involvement in energy matters.

**September**

Media coverage highlighted the intensification of the conflict, particularly in Russia’s Kursk region. Ukrainian forces launched cross-border raids, while Russian forces responded with intensified strikes on Ukrainian territory. The escalation was accompanied by widespread drone warfare, with both sides accusing each other of cross-border drone attacks, targeting military and civilian infrastructure alike.

Russia’s nuclear doctrine came under scrutiny as Moscow issued new warnings linked to Western military support for Ukraine. Russian state broadcasters amplified the rhetoric, portraying nuclear escalation as a necessary deterrent against NATO intervention. In contrast, Western media often framed these statements as intimidation tactics aimed at discouraging further arms deliveries to Ukraine.

Diplomatic discussions remained a focal point. Ukrainian President Volodymyr Zelenskyy pushed for renewed international backing, meeting with Western leaders and advocating a “victory plan.” Meanwhile, alternative peace proposals surfaced from countries such as China and Brazil, as well as Hungarian Prime Minister Viktor Orbán, who attempted to position himself as a mediator. The extent to which these efforts could influence the war’s trajectory remained a subject of debate.

Another major theme was the claim that Iran had supplied Russia with advanced ballistic missiles. The allegations, denied by Tehran, triggered international responses, with Western nations threatening new sanctions.

**October**

Media coverage focused on allegations of North Korean military involvement in Russia’s war against Ukraine. Reports suggested that North Korean troops were aiding Russia by training Russian forces in advanced artillery techniques and providing military supplies. This was portrayed as part of a growing alliance between Moscow and Pyongyang, with Western analysts expressing concerns about the potential for a multi-front war.

There was significant media attention on developments in drone warfare. Ukrainian forces reportedly increased their use of long-range drones to target Russian military installations, particularly in Crimea and Donbas. In response, Russia reinforced its air defence systems, resulting in a series of intercepted drone strikes. Russian state media framed Ukrainian drone strikes as acts of terrorism.

Sanctions against Russia remained a central theme. Western nations imposed additional measures targeting Russian military supply chains and key political figures. These sanctions were presented in the media as a strategic effort to weaken Russia’s military capabilities. Meanwhile, Russian official discourse downplayed the sanctions’ effectiveness, promoting a narrative of resilience and defiance.

Another significant narrative involved the upcoming US presidential election and its potential impact on Russia’s war against Ukraine. Media outlets speculated on how a change in US leadership could affect American foreign policy. Some suggested that a victory by Donald Trump could lead to a shift in US support for Ukraine, influencing the overall trajectory of the war.

**November**

One of the most significant stories was the US government’s decision to authorise the provision of long-range missiles to Ukraine. Western media largely framed the decision as essential for Ukraine’s defence, highlighting its potential to change the dynamics on the ground. In contrast, the Kremlin condemned the decision, portraying it as an act of aggression that could further escalate tensions between the two countries.

Another prominent theme was the reported involvement of North Korean forces in the war, especially following allegations that North Korean troops were deployed in Russia’s Kursk region. This raised alarms about the potential for the conflict to broaden beyond Russia and Ukraine, particularly as the involvement of another regional power such as North Korea suggested that the stakes were growing higher.

Diplomatic efforts continued to play a significant role in the narrative, with a key moment being a phone call between German Chancellor Olaf Scholz and Russian President Vladimir Putin. The call was portrayed in the media as a pivotal attempt at diplomacy, but it also attracted criticism, with some questioning whether it would lead to any meaningful change in the trajectory of the war. While some outlets suggested that the conversation might open doors for peace talks, others were sceptical, arguing that the call was more symbolic than substantive, given the entrenched positions of both sides.

The escalation of sanctions against Russia was another critical theme. Western nations continued to ramp up their economic measures to weaken Russia’s war effort. While Western media portrayed the sanctions as a necessary and effective tool to pressure Russia, Russian state-controlled outlets downplayed their impact, asserting that the country’s resilience in the face of economic hardship would allow it to continue its military campaign.

**December**

Media coverage was dominated by an intensification of hostilities between Russia and Ukraine, with widespread Russian missile and drone attacks targeting Ukraine’s energy infrastructure. Ukrainian forces responded with precision strikes using ATACMS missiles, hitting Russian fuel depots and logistical hubs.

Diplomatic efforts continued. Hungarian Prime Minister Viktor Orbán positioned himself as a mediator, attempting to broker dialogue between Russia and Ukraine. His efforts were met with scepticism within the EU, as Member States questioned Hungary’s alignment with Moscow. Separately, President-elect Donald Trump’s comments on potential peace negotiations sparked debate over Washington’s future approach, with concerns that a second Trump administration might push for a settlement favouring Russian interests.

Energy tensions were a significant theme, with the Russo-Ukrainian gas transit agreement set to expire on 31 December and negotiations remaining deadlocked. Ukrainian President Volodymyr Zelenskyy suggested continuing the transit under the condition that payments bypass Moscow until the war ends, a demand Russia swiftly rejected.

Finally, media reports highlighted growing concerns over critical infrastructure security. Investigations into the sabotage of undersea cables between Finland and Estonia pointed to suspected Russian involvement. In a separate incident, an Azerbaijan Airlines cargo plane crashed under mysterious circumstances, with speculation mounting that Russian air defence systems may have played a role.
